# Global, regional, and national burden of sudden infant death syndrome, 1990–2021: a comprehensive analysis of GBD 2021 data with insights into the impact during the COVID-19 pandemic

**DOI:** 10.3389/fped.2025.1606910

**Published:** 2025-06-24

**Authors:** Yuhan Sun, Haoran Peng, Qiao Chen, Lijie Qin, Ying Ren, Yanwei Cheng

**Affiliations:** ^1^ Henan Key Laboratory for Critical Care Medicine, Zhengzhou Key Laboratory for Critical Care Medicine, Department of Critical Care Medicine, Henan Provincial People’s Hospital, People’s Hospital of Zhengzhou University, People’s Hospital of Henan University, Zhengzhou, Henan, China; ^2^ Department of Emergency, Henan Provincial People’s Hospital, People’s Hospital of Zhengzhou University, People’s Hospital of Henan University, Zhengzhou, China; ^3^ Nursing Department, Air Force Medical Center, PLA, Beijing, China; ^4^ Henan Provincial Key Medicine Laboratory of Nursing, Henan Provincial People’s Hospital, Zhengzhou, China

**Keywords:** sudden infant death syndrome, global burden of disease, disability-adjusted life years, mortality, COVID-19, socio-demographic index

## Abstract

**Background:**

Sudden infant death syndrome (SIDS) remains a leading cause of infant mortality globally. Although the global burden has generally declined over recent decades, the COVID-19 pandemic may have influenced these trends. This study investigates whether the global SIDS burden has changed, particularly during the COVID-19 pandemic.

**Methods:**

Data from the Global Burden of Disease (GBD) 2021 study were analyzed to estimate SIDS mortality and disability-adjusted life years (DALYs) globally, regionally, and nationally. Rates were stratified by sex, age group, socio-demographic index (SDI), and health system level. Projections were made using the Bayesian Age-Period-Cohort model and the the autoregressive integrated moving average (ARIMA) model.

**Results:**

In 2021, global SIDS deaths totaled 30,608, with a mortality rate of 24.16 per 100,000 infants (95% UI, 14.06–32.44). Global DALYs were 2,746,174, at a rate of 2,167.56 per 100,000 infants (95% UI, 1,261.44–2,909.59). Mortality and DALYs rates decreased by 59% from 1990 to 2021, with marked regional differences. Regions with Low SDI and Minimal health systems, particularly Sub-Saharan Africa, had the highest burden, while higher SDI and advanced health system regions reported significant declines. Male infants aged 1–5 months showed higher rates than females. Despite a global decline during the pandemic, temporary increases occurred in countries including China, the Russian Federation, and Monaco. Projections suggest continued declines, predicting a mortality rate of 16.86 per 100,000 infants and DALYs rate of 1,400.41 per 100,000 infants by 2035.

**Conclusions:**

The global SIDS burden has consistently declined since 1990, including during COVID-19, yet significant regional disparities remain. Enhanced healthcare interventions and targeted public health initiatives are crucial, particularly in regions with Low SDI and Minimal health system resources.

## Introduction

Sudden infant death syndrome (SIDS) refers to the sudden and unexplained death of an infant under one year of age after a thorough investigation excludes all other causes (1). Over the past three decades, the global SIDS mortality rate has significantly declined, with a 51% reduction from 1990 to 2019, mainly attributed to public health initiatives such as the ‘Back to Sleep’ campaign (2, 3). However, SIDS continues to be a major cause of infant mortality, particularly in regions like Sub-Saharan Africa, Eastern Europe, and South Asia, where the burden remains disproportionately high (3, 4).

Despite this overall global decline, country-specific deviations have emerged, highlighting vulnerabilities under certain conditions. For example, in the United States, a representative case illustrating such vulnerability, a deviation from the long-term decline in SIDS mortality rate was observed in 2020, with the SIDS rate increasing compared to the previous year, elevating it from the fourth to the third leading cause of infant mortality (5). A subsequent study reported a rise in the SIDS mortality rate per 100,000 live births from 35.2 in 2018 and 33.3 in 2019 to 38.4 in 2020 and 39.8 in 2021, suggesting a significant increase coinciding with the COVID-19 pandemic (6). While changes in death certification practices may partly explain these fluctuations, pandemic-related disruptions in healthcare access and caregiving practices likely contributed as well (7, 8). This case underscores the importance of systematically monitoring SIDS trends globally, particularly during periods of major societal disruption.

Currently, only one global study has assessed the burden of SIDS using data from the Global Burden of Disease (GBD) 2019 study, which predates the COVID-19 pandemic. The GBD approach, previously detailed elsewhere, employs a robust and systematic methodology to quantify health losses globally (9–11). Specifically, it provides standardized metrics, including deaths and disability-adjusted life-years (DALYs), and systematically adjusts for variations and biases inherent in different data sources, ensuring comparability across regions, time periods, age groups, and sexes. This methodological rigor and standardization make the GBD framework particularly suited for estimating and comparing the global burden of diseases with uncertain or inconsistent diagnostic criteria, such as SIDS. However, because previous global analyses rely on data collected before the pandemic, they cannot reflect potential COVID-19-related changes in SIDS burden. Additionally, limited country-level reporting during this period further highlights significant gaps in understanding the global impact of the pandemic on SIDS mortality.

To address this gap, our study utilizes the most recent GBD 2021 data to examine changes in SIDS mortality and DALYs from 1990 to 2021 at the global, regional, and national levels, with a particular focus on the COVID-19 period (2020–2021). Moreover, we explore variations in the SIDS burden across age groups, sexes, socio-demographic index (SDI), and health system levels, providing a comprehensive understanding of disparities in SIDS burden across diverse population groups.

## Methods

### Data source

This study utilized data from the GBD 2021, conducted by the Institute for Health Metrics and Evaluation (IHME) at the University of Washington. The dataset, publicly available through the GBD Collaborative Network website (http://ghdx.healthdata.org), provides a comprehensive analysis of health loss due to 371 diseases, injuries, impairments, and 88 risk factors across 204 countries and territories. The GBD estimation framework addresses missing or incomplete data through a rigorous statistical modeling process. Specifically, mortality and DALY estimates are generated using DisMod-MR 2.1, a Bayesian meta-regression tool that integrates information from multiple data sources, applies model-based imputation, and accounts for uncertainty. Therefore, the estimates we used in this study were already preprocessed and internally consistent, and no additional imputation or dataset integrity assessment was performed during our analysis (11).

For our analysis, data extraction was performed by filtering the GBD Results Tool using the following criteria: cause = “Sudden Infant Death Syndrome”, measure = “Deaths” and “DALYs”, metric = “Rate” and “Number”, age group = “<1 year” (and “<28 days”, “1–5 months”, “6–11 months”), sex = “Both” (and “Male”, “Female”), and years = “1990”-“2021”. No additional imputation or interpolation was applied, as the GBD data already incorporated standardized modeling and smoothing procedures to address missing values and sampling uncertainty.

### Disease burden calculation

In this study, the burden of SIDS was quantified using two key indicators: the mortality rate and DALYs. The mortality rate refers to SIDS-related deaths per 100,000 infants, reflecting the direct fatal impact of the condition. DALYs represent the sum of years of life lost (YLLs) due to premature mortality and years lived with disability (YLDs). Given that SIDS is a strictly fatal condition, DALYs are equivalent to YLLs, as there are no survivors with disability. Consequently, the mortality rate and DALYs rate are directly proportional to each other.

While age-standardized rates (ASRs) are commonly used to adjust for population age structure, this study primarily focused on age-specific crude mortality and DALYs rates for infants under one year of age. Since SIDS predominantly affects this age group, our analysis focused on calculating and comparing these rates across regions, sexes, and health system grouping levels.

### Socioeconomic development and SDI

To explore regional variations in SIDS burden, we used the SDI, a composite measure that combines income per capita, education attainment, and fertility rates to assess the level of socioeconomic development within a region (11). The SDI ranges from 0 to 1, with higher values indicating greater levels of socioeconomic development. Based on the GBD 2021 quintile-based classification, regions were grouped into five categories: High (≥0.81), High-middle (0.71–0.81), Middle (0.61–0.71), Low-middle (0.46–0.61), and Low (≤0.45). These categories are not evenly spaced, as they are defined by quintile distributions across all GBD regions, which may result in varying interval widths depending on the data distribution.

### Health system grouping levels

To further investigate the role of healthcare access and infrastructure in SIDS burden, countries were categorized into four health system grouping levels: Advanced, Basic, Limited, and Minimal health systems. This classification was developed based on the health system capacity profiles provided within the GBD framework, primarily derived by the IHME. While not directly adopted from the World Bank, this grouping draws upon global health indicators, including healthcare resources, service quality, and access to essential medical care, that are informed by data from multiple international sources such as the World Bank, WHO, and other national surveys. This stratification enables a contextualized understanding of how variations in healthcare system strength influence SIDS outcomes (12).

### Statistical analysis

Statistical analyses were performed to examine trends in SIDS mortality and DALYs at global, regional, and national levels, as well as across different SDI and health system grouping levels. To analyze mortality and DALYs rates of SIDS by SDI, smoothing splines models were used to predict expected rates, focusing on understanding the dose-response relationships between SIDS rates and SDI across 21 GBD regions, rather than primarily focusing on model fit. Stratified analyses were performed to examine demographic differences in SIDS burden by age groups (<28 days, 1–5 months, and 6–11 months) and sex (male and female). Uncertainty intervals (UIs) were calculated as the 25th and 975th ordered values from 1,000 posterior draws, corresponding to a 95% uncertainty interval under the Bayesian framework used in the GBD study. These intervals reflect both data-driven variability and model-based uncertainty, and represent the range within which the true values are likely to lie with 95% probability.

To project future global trends in SIDS burden from 2021 to 2035, we utilized a Bayesian Age-Period-Cohort (BAPC) model to capture long-term trends and cohort-specific effects. This model was selected for its ability to decompose the burden into structured components, providing smoothed and robust projections suitable for long-range epidemiological forecasting. Additionally, we applied the autoregressive integrated moving average (ARIMA) model to forecast SIDS burden by the specific GBD region, SDI, gender, age group, and health system grouping level from 2021 to 2030. The ARIMA model was chosen for its strength in capturing short-term temporal fluctuations, which may reflect the influence of recent policy interventions or sudden socio-environmental events.

## Results

### Global burden of SIDS

In 2021, the global estimated number of SIDS deaths was 30,608 (95% UI, 17,810–41,094), corresponding to a mortality rate of 24.16 per 100,000 infants (95% UI, 14.06–32.44). Global DALYs for SIDS amounted to 2,746,174 (95% UI, 1,598,180–3,686,290), resulting in a DALYs rate of 2,167.56 per 100,000 infants (95% UI, 1,261.44–2,909.59) (Table 1). Both mortality and DALYs rates showed a consistent annual decline from 1990 to 2021, with a reduction of 59% (95% UI, −84% to −10%) (Table 1; Supplementary Figure S1). Between 2019 and 2021, the global decline in rates was 10% (95% UI, −61% to 105%). Notably, the largest reduction occurred from 2019 to 2020 [−8%, (95% UI, −60% to 112%)], while the decline from 2020 to 2021 was smaller [−2% (95% UI, −58% to 126%)].

**Table 1:** Global trends in SIDS deaths and DALYs from 1990 to 2021, with percentage change in rates.

Year/Change (%)	Deaths (95% UI)		DALYs (95% UI)	
	Number	Rate (per 100,000)	Number	Rate (per 100,000)
1990	75,718 (45,928–114,652)	59.27 (35.95–89.75)	6,794,660 (4,121,026–10,289,656)	5318.82 (3,225.91–8,054.68)
2019	35,521 (20,892–47,432)	26.88 (15.81–35.89)	3,186,992 (1,874,798–4,254,987)	2,411.72 (1,418.73–3,219.91)
2020	31,874 (18,557–43,319)	24.69 (14.37–33.55)	2,859,783 (1,665,146–3,886,254)	2,215.01 (1,289.72–3,010.05)
2021	30,608 (17,810–41,094)	24.16 (14.06–32.44)	2,746,174 (1,598,180–3,686,290)	2,167.56 (1,261.44–2,909.59)
Percentage change in rates between 1990 and 2021 (%)	−60 (−84 to −11)	−59 (−84 to −10)	−60 (−84 to −11)	−59 (−84 to −10)
Percentage change in rates between 2019 and 2021 (%)	−14 (−62 to 97)	−10 (−61 to 105)	−14 (−62 to 97)	−10 (−61 to 105)
Percentage change in rates between 2019 and 2020 (%)	−10 (−61 to 107)	−8 (−60 to 112)	−10 (−61 to 107)	−8 (−60 to 112)
Percentage change in rates between 2020 and 2021 (%)	−4 (−59 to 121)	−2 (−58 to 126)	−4 (−59 to 121)	−2 (−58 to 126)

DALYs, disability-adjusted life years; SIDS, sudden infant death syndrome; UI, uncertainty interval.

### Regional burden of SIDS

**Figure 1:**
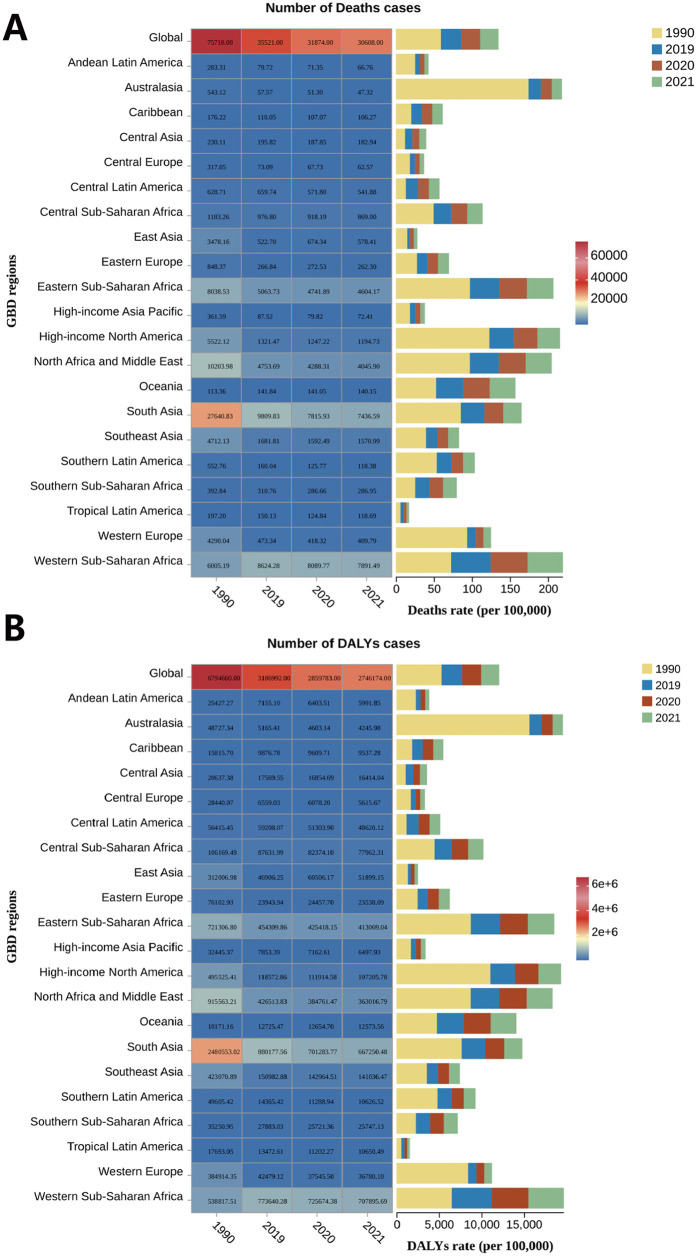
Regional number and rates of **(A)** deaths and **(B)** DALYs of SIDS in 1990 and 2019–2021.

At the regional level, Western Sub-Saharan Africa had the highest SIDS burden in 2021, with a mortality rate of 46.60 per 100,000 infants (95% UI, 22.80–70.22) and a DALYs rate of 4,179.95 per 100,000 infants (95% UI, 2,046.05–6,299.52). Other regions with high burden included Eastern Sub-Saharan Africa and North Africa and Middle East. Conversely, Tropical Latin America [mortality rate 3.48 per 100,000 infants (95% UI, 2.68–4.40); DALYs rate 311.90 per 100,000 infants (95% UI, 240.57–395.07)], East Asia [mortality rate 4.84 (95% UI, 2.10–8.09); DALYs rate 434.58 (95% UI, 188.83–725.69)], and Andean Latin America [mortality rate 5.46 (95% UI, 3.12–8.91); DALYs rate 490.21 (95% UI, 280.26–799.71)] recorded the lowest burden (Supplementary Table S1; Figure 1). From 1990 to 2021, 20 out of 21 regions experienced a reduction in both mortality and DALYs rates, with Australasia showing the largest decrease [−92% (95% UI, −95% to −89%)], and Central Asia the smallest [−25% (95% UI, −68% to 201%)]. However, Central Latin America saw a slight increase in both mortality and DALYs rates [7% (95% UI, −32% to 68%)], though this increase was slower from 1990 to 2021 compared to 1990–2019, largely due to a reduction observed between 2019 and 2021 [−13% (95% UI, −48% to 48%)]. Except for Central Latin America, the other 18 regions also showed a decline in both rates between 2019 and 2021, with South Asia experiencing the most significant reduction [−22% (95% UI, −73% to 135%)] and the Caribbean the least [−2% (95% UI, −78% to 309%)]. Interestingly, East Asia [41% (95% UI, −63% to 430%)] and Eastern Europe [8% (95% UI, −27% to 58%)] saw an increase in both rates during this period. Specifically, East Asia experienced an increase in rates from 2019 to 2020, followed by a decline in 2020–2021, while Eastern Europe saw an increase in 2020, with a slowdown in the rise during 2021 (Supplementary Table S1).

### SIDS burden by country

**Figure 2:**
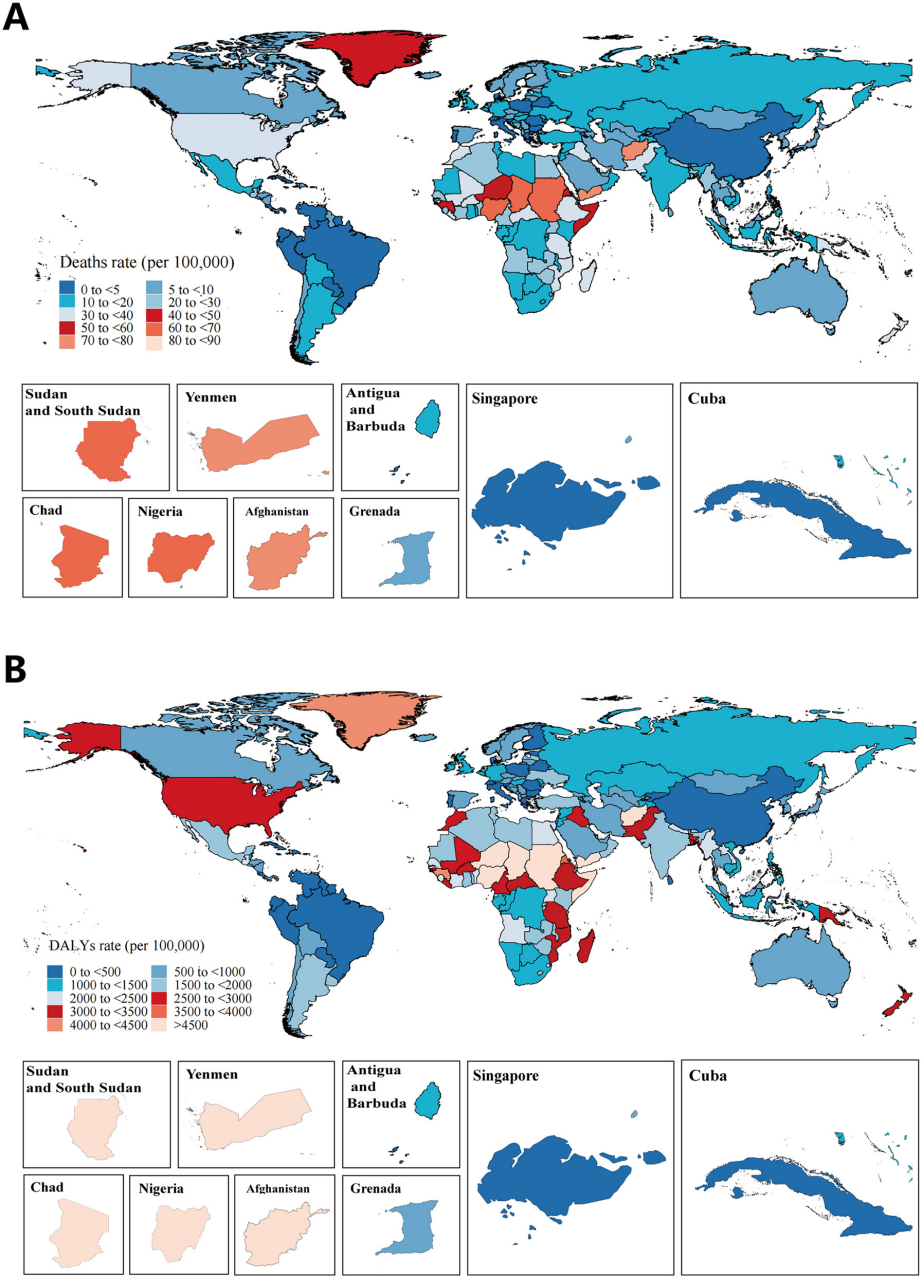
Rates of **(A)** deaths and **(B)** DALYs of SIDS in 204 countries and territories in 2021.

In 2021, countries with the highest SIDS mortality and DALYs rates included South Sudan, Yemen, Afghanistan, Chad, Nigeria, and Sudan. These countries are predominantly located in Western and Eastern Sub-Saharan Africa, as well as North Africa and Middle East, regions identified as having the highest global burden of SIDS. In contrast, countries such as Antigua and Barbuda, Grenada, Singapore, and Cuba reported the lowest rates of SIDS (Figure 2; Supplementary Table S2). From 1990 to 2021, most countries showed a decreasing trend in both mortality and DALYs rates, with the most substantial reductions observed in Grenada, Australia, Canada, Austria, and Belgium. However, some countries, such as Azerbaijan, Georgia, Tajikistan, Mauritius, Dominica, the Republic of Moldova, and several countries in Central Latin America (including Panama, Venezuela, and the United Mexican States) experienced an increase in both rates. Notably, the rate of increase in these countries was lower in the period from 1990 to 2021 than in the period from 1990 to 2019, largely due to a decline observed from 2019 to 2021. Additionally, several countries, including China [45% (95% UI, −64% to 492%)], the Russian Federation [12% (95% UI, −19% to 53%)], and Monaco [113% (95% UI, −66% to 1220%)], showed an upward trend between 2019 and 2021, specifically in 2020, which is consistent with the regional increase observed in East Asia and Eastern Europe.

### Burden of SIDS by sex and age

In 2021, SIDS mortality rate was higher in males than females globally (Supplementary Table S3). The mortality rate for males was 25.31 per 100,000 infants (95% UI, 12.28–38.16), compared to 22.93 per 100,000 infants (95% UI, 10.66–31.90) for females. Similarly, the DALYs rate for males was 2,270.69 per 100,000 infants (95% UI, 1,102.33–3,424.11), higher than that for females at 2,057.31 per 100,000 infants (95% UI, 956.74–2,861.75). This gender disparity in SIDS burden has persisted since 2009, with males consistently exhibiting higher rates than females (Supplementary Figure S2). From 1990 to 2021, both male and female mortality and DALYs rates showed a decline. However, males experienced a slightly smaller reduction [−56% (95% UI, −86% to 26%)] compared to females [−63% (95% UI, −90% to 10%)]. This pattern continued through the 2019–2021 period, with a sharper decline between 2019 and 2020 [−8%, (95% UI, −9% to −8%)] and a more modest reduction from 2020 to 2021 [−2%, (95% UI, −3% to −2%)].

**Figure 3:**
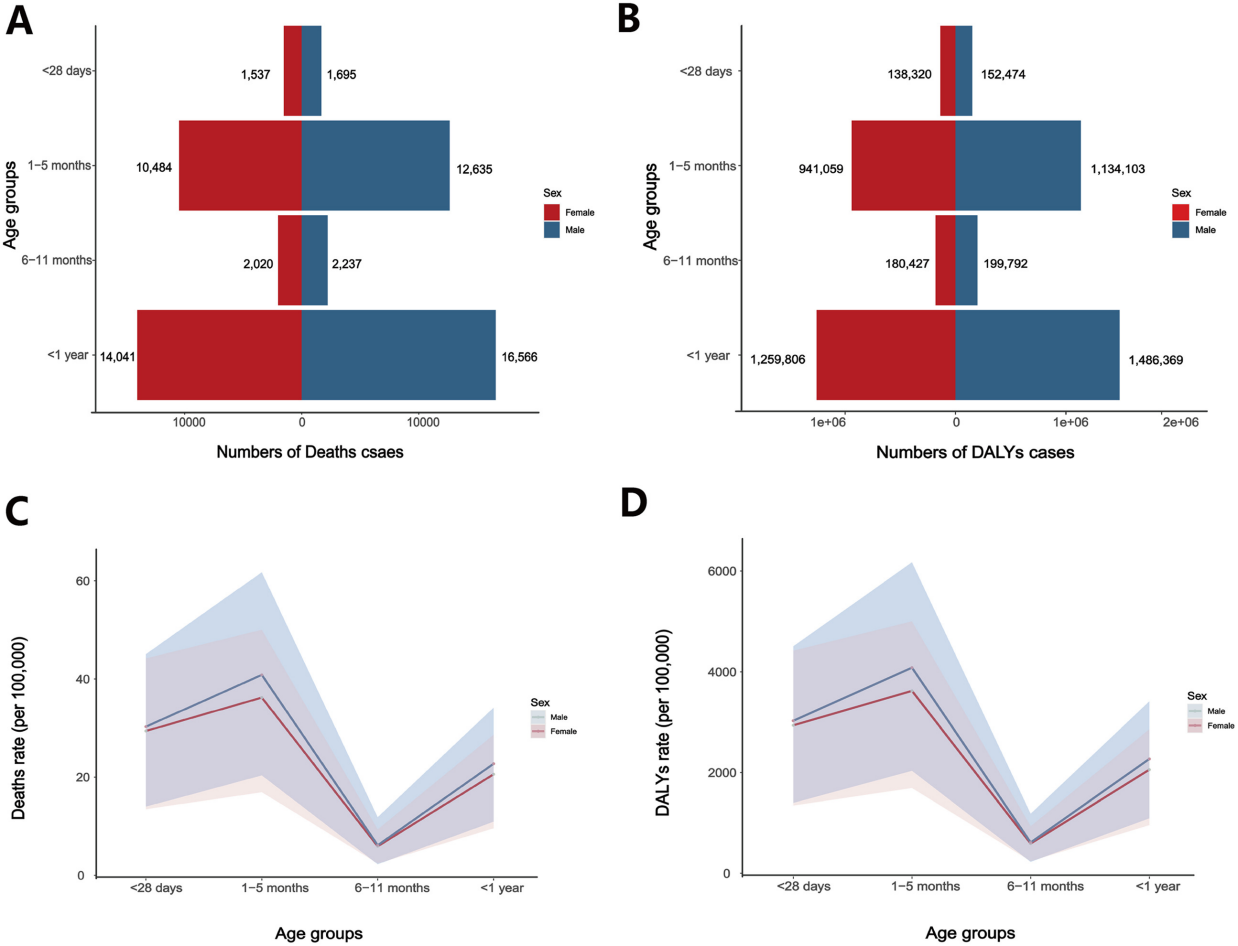
Global number and rates of SIDS deaths and DALYs by sex and age in 2021. **(A)** Number of deaths and **(B)** number of DALYs by age group and sex; **(C)** SIDS death rates and **(D)** DALYs rates per 100,000 population across age groups, stratified by sex.

Regarding age groups, the highest SIDS mortality and DALYs rates in 2021 were observed in the 1–5 months group, followed by the <28 days group, and finally the 6–11 months group (Figure 3; Supplementary Table S4). Males had higher rates than females in each age group, with the largest gender disparity in the 1–5 months group (mortality rate 45.49 per 100,000 infants [95% UI, 22.78–68.90] vs. 40.35 per 100,000 infants [95% UI, 18.87–55.74]; DALYs rate 4,083.49 per 100,000 infants [95% UI, 2,044.73–6,184.42] vs. 3,621.67 per 100,000 infants [95% UI, 1,693.54–5,003.09]). The gender gap was smaller in the <28 days and 6–11 months groups. From 1990 to 2019, as well as during 2019–2021, SIDS rates declined in all three age groups, with females experiencing slightly greater reductions than males (Supplementary Figure S3; Supplementary Table S4). The largest reduction from 1990 to 2019 occurred in the <28 days group [−60% (95% UI, −86% to 15%)], while the most significant decrease from 2019 to 2021 was observed in the 1–5 months group [−11% (95% UI, −61% to 99%)].

### Burden of SIDS by SDI

In 2021, the SIDS mortality and DALYs rates followed a consistent ranking from highest to lowest: Low SDI, Low-middle SDI, High SDI, Middle SDI, and High-middle SDI regions (Supplementary Figure S4; Supplementary Table S5). Although this order broadly reflects the expected SDI gradient, the slightly higher burden observed in High SDI compared to Middle SDI likely reflects regional variations and does not contradict the overall inverse relationship between SDI and SIDS burden. This ranking has remained stable since 1992, with High SDI regions surpassing Low-middle SDI regions between 1990 and 1992. Both rates across all SDI regions have continuously declined from 1990 to 2021. The most significant reduction was observed in High SDI regions [−79% (95% UI, −81% to −76%)], while the smallest reduction occurred in Low SDI regions [−50% (95% UI, −83% to 49%)]. Between 2019 and 2021, SIDS burden decreased in all SDI regions except for High-middle SDI. Notably, the decline was more pronounced from 2019 to 2020 compared to the period between 2020 and 2021. The greatest reduction was seen in Low-middle SDI regions [−17% (95% UI, −69% to 123%)], while High-middle SDI regions experienced a slight increase in SIDS burden during the 2019–2021 period [4% (95% UI, −42% to 79%)]. This increase was driven by a 5% rise from 2019 to 2020, followed by a minor decrease of 1% from 2020 to 2021. Additionally, when SIDS rates and SDI values were compared across regions, a consistent negative correlation was found, indicating that higher SDI levels were associated with a lower burden of SIDS in the respective GBD regions (Supplementary Figure S5).

### Burden of SIDS by health system grouping levels

From 1990 to 2021, SIDS rates decreased across all four health system grouping levels, with substantial reductions observed (Supplementary Figure S6; Supplementary Table S6). The most significant decline occurred in the Advanced health system grouping, with a reduction of 78% (95% UI, −79% to −77%). Although the Minimal health system grouping achieved a 52% reduction (95% UI, −53% to −48%), it still recorded the highest SIDS rates in 2021, with a mortality rate of 41.19 per 100,000 infants (95% UI, 20.47–62.03) and a DALYs rate of 3,695.79 per 100,000 infants (95% UI, 1,837.12–5,563.35). In contrast, the Basic health system grouping, rather than the Advanced grouping, exhibited the lowest SIDS rates, with a mortality rate of 10.73 per 100,000 infants (95% UI, 6.69–14.91) and a DALYs rate of 963.19 per 100,000 infants (95% UI, 600.41–1,338.41). Between 2019 and 2021, SIDS rates declined across all health system grouping levels, with the Limited health system grouping showing the largest reduction of 16% (95% UI, −18% to −14%). The most notable reduction occurred between 2019 and 2020, with a decline of 12% (95% UI, −14% to −11%).

### Future forecasts of burden of SIDS

**Figure 4:**
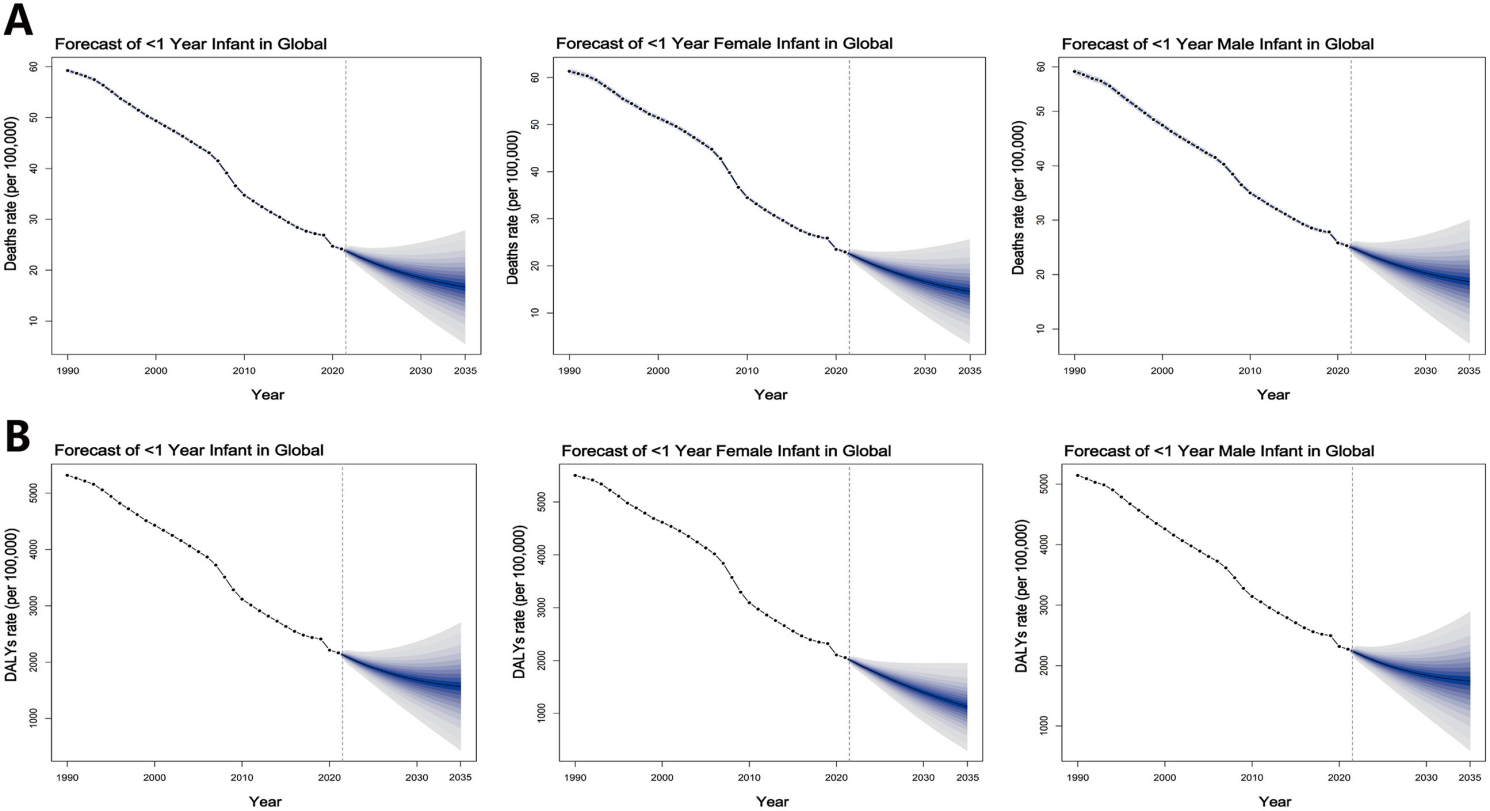
Future forecasts of global **(A)** deaths and **(B)** DALYs rates of SIDS from 2021 to 2035 by BAPC model.

Based on the BAPC model predictions, the global burden of SIDS is projected to continue decreasing from 2021 to 2035 (Figure 4; Supplementary Table S7). The mortality rate is expected to decline from 24.16 per 100,000 infants in 2021 to 16.86 per 100,000 infants by 2035. Similarly, the DALYs rate is predicted to decrease from 2,167.56 per 100,000 infants in 2021 to 1,400.41 per 100,000 infants in 2035. By 2035, the SIDS burden for males are projected to remain higher than those for females, with a projected male mortality rate of 18.84 per 100,000 infants compared to 14.69 per 100,000 infants for females. The DALYs rate for males is predicted to be 1,718.05 per 100,000 infants, while for females, it is expected to be 1,124.80 per 100,000 infants. Additionally, the ARIMA model predicts that between 2021 and 2030, SIDS mortality and DALYs rates for males in the 1–5 month age group will continue to decline in Low SDI regions, Western Sub-Saharan Africa, and within the Minimal health system grouping (Supplementary Figure S7; Supplementary Table S8).

## Discussion

To our knowledge, this study represents the second global analysis of the burden of SIDS and the first to incorporate data from the GBD 2021 study. It builds upon the foundational work by Park et al., who conducted the initial global assessment using GBD 2019 data to evaluate SIDS mortality and DALYs across 204 countries from 1990 to 2019 (3). Their study revealed a global decline in SIDS burden, substantial regional disparities, and a strong inverse association between SDI and SIDS rates. However, their analysis did not include the COVID-19 pandemic period and focused primarily on retrospective trends. In contrast, our study extends the timeline through 2021 to assess potential pandemic-related impacts and further broadens the scope by incorporating projections to 2035, stratified analyses by age group and sex, and comparisons across health system classifications. These additions offer a more comprehensive and up-to-date understanding of global, regional, and national trends in SIDS burden.

The overall 59% decline in the global SIDS burden highlights the positive impact of evidence-based public health initiatives, such as the “Back to Sleep” campaign, which promoted supine sleeping and safer sleep environments. These interventions contributed to reductions in SIDS rates ranging from 42% to 92% across different regions (13–15). However, progress has been uneven, with significant regional disparities. For instance, Western Sub-Saharan Africa continues to experience alarmingly high SIDS mortality rates, with a rate of 46.6 per 100,000 infants in 2021, while Tropical Latin America reports considerably lower rates, consistent with findings from the GBD 2019 study (3). Notably, Western Sub-Saharan Africa, categorized as a Low SDI region, faces challenges such as limited healthcare access, overcrowded living conditions, and unsafe sleep practices like bed-sharing, which exacerbate the risks of SIDS. In contrast, Tropical Latin America, classified within the Middle SDI region, benefits from better healthcare infrastructure and safer sleep environments, leading to lower mortality rate. These regional disparities highlight the important role that socioeconomic development plays in influencing the burden of SIDS, with lower SDI regions generally experiencing higher mortality rate. Additionally, while Australasia (High SDI) experienced the largest reduction in SIDS mortality and DALYs rates, Central Asia (High-middle SDI) saw the smallest reduction. Similarly, the paradoxical rise in SIDS burden in Central Latin America (Middle SDI), where the mortality rate has increased by 7% since 1990, further underscores the complexity of SIDS epidemiology. All these observations suggest that the decline in SIDS burden is not solely determined by economic status or SDI level. Other factors, such as the availability and effectiveness of public health interventions, play a crucial role in influencing SIDS outcomes. For example, regions with widespread prenatal education, robust tobacco control, and extensive safe sleep initiatives have seen greater reductions in SIDS mortality, even if their SDI is not among the highest (16–21). Another important and somewhat paradoxical finding in this study is that regions with Basic health systems outperformed those with Advanced health systems in SIDS mortality. This could reflect the success of low-cost, high-impact interventions in resource-limited settings, such as promoting safe sleep practices and encouraging tobacco control. Overall, the disparities observed across regions emphasize the need for tailored, multifactorial interventions that address both structural healthcare issues and social determinants of health. Ensuring that interventions are adapted to the specific needs of each region, whether through improving maternal health, expanding access to prenatal care, or promoting safe sleep practices, will be critical to further reducing the global SIDS burden.

Our study reaffirms the well-established gender disparity in SIDS mortality, with male infants exhibiting higher rates than females (22, 23). This persistent gap suggests that male infants may be particularly vulnerable to sleep-related risks, potentially due to sex-specific genetic and biological factors (24). However, the precise causes of the higher SIDS mortality in males remain unclear. Additionally, our analysis of age-specific mortality rate reveals the highest SIDS rates in the 1–5 month age group, aligning with previous research that shows 95% of SIDS deaths occur between 1 and 6 months of age (25, 26). This period is marked by significant developmental changes, including respiratory, autonomic, and cardiac maturation, which may heighten infants’ vulnerability to sleep-related risks (25, 27). Given the importance of safe sleep practices, it is concerning that many parents worldwide still lack sufficient awareness of SIDS. Public health campaigns and healthcare professionals should play a key role in educating parents about safe sleep guidelines and the higher risks for infants aged 1–6 months, as well as for male infants. By emphasizing these issues through education, parents will be encouraged to be more vigilant and take necessary precautions, such as following safe sleep guidelines and closely monitoring their infants during this critical period.

The COVID-19 pandemic likely impacted SIDS by increasing pressures on families and caregivers, which limited access to clinical follow-up and support services such as well-baby visits and programs like the Women, Infants, and Children Supplemental Nutrition Program. This lack of access may have raised the risk of SIDS (28). Clinical visits are critical for promoting safe sleep practices, smoking cessation, immunizations, and breastfeeding (8, 29). Additionally, changes in childcare arrangements during the pandemic may have led to unfamiliar sleep positions and unsafe practices, further increasing SIDS risk (30). While the global SIDS burden continued to decline during 2020–2021, significant regional and national heterogeneities emerged. For instance, China in East Asia, the Russian Federation in Eastern Europe, and Monaco in Western Europe experienced temporary increases in SIDS rates during the pandemic, which may have been linked to delayed healthcare-seeking behavior or overwhelmed health systems (31, 32). It is worth noting that both China and the Russian Federation are high-middle SDI countries, and our findings also show that high-middle SDI regions experienced a slight increase in the SIDS burden during the pandemic. In contrast to the rise in SIDS rates observed in the United States during the COVID-19 pandemic, where the mortality rate increased from 33.3 per 100,000 live births in 2019 to 39.8 in 2021, our study did not find a general increase in SIDS mortality during this period (6, 33). This discrepancy is likely reflective of diagnostic reclassification rather than an actual increase in biological risk (7). Overburdened forensic systems increasingly defaulted to coding SIDS for deaths previously classified as “accidental suffocation” or “ill-defined causes.” In fact, the global trend from 2019 to 2021 remained largely consistent with the decline observed from 1990 to 2019. This suggests that, although the COVID-19 pandemic disrupted healthcare systems and caregiving practices in certain regions, the global impact on SIDS rates may have been less pronounced than initially anticipated.

This study is based on estimates from the GBD 2021 dataset, which integrates multiple data sources across countries and time periods. In some regions, particularly low-resource settings, the availability and completeness of primary data remain limited, increasing the likelihood of underreporting or misclassification. While this analysis incorporates macro-level indicators such as SDI and health system capacity, it does not account for individual behavioral or environmental risk factors that may influence SIDS outcomes, such as maternal smoking, unsafe sleep environments, or parental education. The influence of the COVID-19 pandemic is also difficult to isolate, as it may have affected healthcare access, care-seeking behavior, and death certification practices in complex and heterogeneous ways. Additionally, the use of standardized modeling approaches across vastly different settings can introduce estimation bias where local inputs are sparse or uncertain. In such cases, some estimates, especially for small populations or low-surveillance countries, exhibited relatively wide uncertainty intervals. These wide ranges reflect statistical imprecision rather than errors and should prompt caution in country-level interpretations. More robust conclusions are best drawn from aggregated patterns at the regional or global scale, where data density and model reliability are stronger.

## Conclusion

In conclusion, although the global burden of SIDS has substantially declined since 1990, pronounced disparities persist, especially in Low SDI regions and countries with Minimal health system capacity. Male infants aged 1–5 months continue to face elevated risk. While the overall burden decreased during the COVID-19 pandemic, some countries experienced temporary increases, likely linked to healthcare disruptions. To accelerate progress, efforts should prioritize strengthening prenatal and neonatal care services, expanding community-based education on safe sleep practices, and improving the accuracy of SIDS surveillance and death reporting systems. By integrating updated GBD 2021 data, age- and sex-specific analyses, and future projections, this study provides timely evidence to guide targeted, equity-focused public health interventions.

## Data Availability

The original contributions presented in the study are included in the article/Supplementary Material, further inquiries can be directed to the corresponding authors.
