# Supplementary material for: Global, regional, and national burden of sudden infant death syndrome, 1990–2021: a comprehensive analysis of GBD 2021 data with insights into the impact during the COVID-19 pandemic
Source: Front Pediatr. 2025 Jun 24;13:1606910. doi: 10.3389/fped.2025.1606910 (PMC12234550; doi:10.3389/fped.2025.1606910)
Supplement: Supplementary file 1 [file Datasheet1.docx]

**Supplementary Table 1. GBD region-specific trends in SIDS deaths and DALYs from 1990 to 2021, with percentage change in rates**

| **GBD regions** | **Deaths (95% UI)** | | | | | | | | | | | | | **DALYs (95% UI)** | | | | | | | | | | | | | | |
| --- | --- | --- | --- | --- | --- | --- | --- | --- | --- | --- | --- | --- | --- | --- | --- | --- | --- | --- | --- | --- | --- | --- | --- | --- | --- | --- | --- | --- |
|  | **Number** | | | | **Rate (per 100,000)** | | | | **Percentage change in rates (%)** | | | | | **Number** | | | | **Rate (per 100,000)** | | | | **Percentage change in rates (%)** | | | | | | |
|  | **1990** | **2019** | **2020** | **2021** | **1990** | **2019** | **2020** | **2021** | **From 1990 to 2021** | **From 1990 to 2019** | **From 2019 to 2021** | **From 2019 to 2020** | **From 2020 to 2021** | **1990** | **2019** | **2020** | **2021** | **1990** | **2019** | **2020** | **2021** | **From 1990 to 2021** | **From 1990 to 2019** | **From 2019 to 2021** | | **From 2019 to 2020** | **From 2020 to 2021** | |
| **Global** | 75,718 ( 45,928 to 114,652) | 35,521 (20,892 to 47,432) | 31,874 (18,557 to 43,319) | 30,608 (17,810 to 41,094) | 59.27 (35.95 to 89.75) | 26.88 (15.81 to 35.89) | 24.69 (14.37 to 33.55) | 24.16 (14.06 to 32.44) | -59 (-84 to -10) | -55 (-82 to 0) | -10 (-61 to 105) | -8 (-60 to 112) | -2 (-58 to 126) | 6,794,660 (4,121,026 to 10,289,656) | 3,186,992 (1,874,798 to 4,254,987) | 2,859,783 (1,665,146 to 3,886,254) | 2,746,174 (1,598,180 to 3,686,290) | 5318.82 (3225.91 to 8054.68) | 2411.72 (1418.73 to 3219.91) | 2215.01 (1289.72 to 3010.05) | 2167.56 (1261.44 to 2909.59) | -59 (-84 to -10) | -55 (-82 to 0) | -10 (-61 to 105) | -8 (-60 to 112) | | | -2 (-58 to 126) |
| **Andean Latin America** | 283 (135 to 497) | 80 (45 to 128) | 71 (41 to 117) | 67 (38 to 109) | 25.32 (12.09 to 44.39) | 6.45 (3.68 to 10.38) | 5.78 (3.35 to 9.52) | 5.46 (3.12 to 8.91) | -78 (-93 to -26) | -75 (-92 to -14) | -15 (-70 to 142) | -10 (-68 to 159) | -6 (-67 to 166) | 25,427 (12,138 to 44,572) | 7,155 (4,084 to 11,503) | 6,404 (3,706 to 10,542) | 5,992 (3,426 to 9,775) | 2,272.80 (1,084.94 to 3,984.04) | 579.24 (330.58 to 931.21) | 519.09 (300.38 to 854.59) | 490.21 (280.26 to 799.71) | -78 (-93 to -26) | -75 (-92 to -14) | -15 (-70 to 142) | | -10 (-68 to 159) | -6 (-67 to 166) | |
| **Australasia** | 543 (509 to 578) | 58 (45 to 71) | 51 (40 to 65) | 47 (35 to 61) | 174.44 (163.51 to 185.75) | 16.11 (12.73 to 19.86) | 14.52 (11.21 to 18.49) | 13.37 (9.97 to 17.35) | -92 (-95 to -89) | -91 (-93 to -88) | -17 (-50 to 36) | -10 (-44 to 45) | -8 (-46 to 55) | 48,727 (45,674 to 51,887) | 5,165 (4,082 to 6,367) | 4,603 (3,554 to 5,863) | 4,246 (3,165 to 5,510) | 15,650.52 (14,669.77 to 16,665.38) | 1,445.54 (1,142.46 to 1,781.76) | 1,302.49 (1,005.58 to 1,659.02) | 1,200.04 (894.49 to 1,557.25) | -92 (-95 to -89) | -91 (-93 to -88) | -17 (-50 to 36) | | -10 (-44 to 45) | -8 (-46 to 55) | |
| **Caribbean** | 176 (94 to 345) | 110 (50 to 219) | 107 (48 to 203) | 106 (47 to 203) | 20.29 (10.82 to 39.66) | 13.98 (6.39 to 27.79) | 13.71 (6.18 to 26.02) | 13.71 (6.05 to 26.14) | -32 (-85 to 142) | -31 (-84 to 157) | -2 (-78 to 309) | -2 (-78 to 307) | 0 (-77 to 323) | 15,816 (8,440 to 30,930) | 9,877 (4,519 to 19,632) | 9,610 (4,335 to 18,237) | 9,537 (4,205 to 18,185) | 1,820.59 (971.52 to 3,560.47) | 1,254.49 (573.97 to 2,493.61) | 1,230.36 (555.01 to 2,334.94) | 1,230.63 (542.55 to 2,346.42) | -32 (-85 to 142) | -31 (-84 to 157) | -2 (-78 to 309) | | -2 (-78 to 307) | 0 (-77 to 323) | |
| **Central Asia** | 230 (90 to 344) | 196 (127 to 303) | 188 (121 to 290) | 183 (117 to 285) | 12.01 (4.68 to 17.95) | 9.66 (6.25 to 14.96) | 9.25 (5.95 to 14.30) | 9.05 (5.79 to 14.09) | -25 (-68 to 201) | -20 (-65 to 220) | -6 (-61 to 125) | -4 (-60 to 129) | -2 (-60 to 136) | 20,637 (8,036 to 30,843) | 17,570 (11,361 to 27,196) | 16,855 (10,855 to 26,051) | 16,414 (10,492 to 25,539) | 1,076.92 (419.36 to 1,609.50) | 866.85 (560.54 to 1,341.79) | 829.76 (534.38 to 1,282.49) | 812.19 (519.17 to 1,263.72) | -25 (-68 to 201) | -20 (-65 to 220) | -6 (-61 to 125) | | -4 (-60 to 129) | -2 (-60 to 136) | |
| **Central Europe** | 317 (255 to 388) | 73 (55 to 96) | 68 (48 to 92) | 63 (43 to 88) | 18.44 (14.84 to 22.54) | 6.55 (4.94 to 8.58) | 6.24 (4.46 to 8.49) | 5.94 (4.12 to 8.39) | -68 (-82 to -43) | -64 (-78 to -42) | -9 (-52 to 70) | -5 (-48 to 72) | -5 (-51 to 88) | 28,440 (22,894 to 34,771) | 6,559 (4,939 to 8,582) | 6,078 (4,343 to 8,269) | 5,616 (3,900 to 7,933) | 1,653.74 (1,331.27 to 2,021.89) | 588.22 (442.91 to 769.61) | 560.44 (400.41 to 762.40) | 533.00 (370.14 to 752.98) | -68 (-82 to -43) | -64 (-78 to -42) | -9 (-52 to 70) | | -5 (-48 to 72) | -5 (-51 to 88) | |
| **Central Latin America** | 629 (547 to 732) | 660 (530 to 821) | 572 (432 to 746) | 542 (398 to 738) | 13.06 (11.36 to 15.21) | 16.04 (12.87 to 19.95) | 14.39 (10.87 to 18.76) | 13.99 (10.29 to 19.06) | 7 (-32 to 68) | 23 (-15 to 76) | -13 (-48 to 48) | -10 (-46 to 46) | -3 (-45 to 75) | 56,415 (49,087 to 65,707) | 59,208 (47,518 to 73,640) | 51,304 (38,765 to 66,894) | 48,620 (35,751 to 66,228) | 1,171.62 (1,019.42 to 1,364.59) | 1,439.39 (1,155.20 to 1,790.24) | 1,290.68 (975.24 to 1,682.90) | 1,255.30 (923.05 to 1,709.90) | 7 (-32 to 68) | 23 (-15 to 76) | -13 (-48 to 48) | | -10 (-46 to 46) | -3 (-45 to 75) | |
| **Central Sub-Saharan Africa** | 1,183 (411 to 2,551) | 977 (415 to 1,869) | 918 (392 to 1,797) | 869 (371 to 1,661) | 49.72 (17.28 to 107.20) | 22.72 (9.66 to 43.46) | 21.38 (9.12 to 41.85) | 20.23 (8.63 to 38.66) | -59 (-92 to 123) | -54 (-91 to 151) | -11 (-80 to 300) | -6 (-79 to 333) | -5 (-79 to 324) | 106,169 (36,928 to 228,841) | 87,632 (37,283 to 167,588) | 82,374 (35,153 to 161,166) | 77,962 (33,278 to 148,928) | 4,460.83 (1,551.58 to 9,615.01) | 2,038.29 (867.19 to 3,898.03) | 1,918.31 (818.63 to 3,753.21) | 1,815.08 (774.75 to 3,467.28) | -59 (-92 to 123) | -54 (-91 to 151) | -11 (-80 to 300) | | -6 (-79 to 333) | -5 (-79 to 324) | |
| **East Asia** | 3,478 (1,922 to 5,464) | 523 (231 to 855) | 674 (294 to 1,113) | 578 (251 to 966) | 14.97 (8.28 to 23.53) | 3.45 (1.53 to 5.64) | 5.04 (2.19 to 8.31) | 4.84 (2.10 to 8.09) | -68 (-91 to -2) | -77 (-94 to -32) | 41 (-63 to 430) | 46 (-61 to 445) | -4 (-75 to 269) | 312,007 (172,446 to 490,146) | 46,906 (20,765 to 76,734) | 60,506 (26,352 to 99,876) | 51,899 (22,550 to 86,664) | 1,343.26 (742.42 to 2,110.19) | 309.19 (136.87 to 505.80) | 451.84 (196.78 to 745.84) | 434.58 (188.83 to 725.69) | -68 (-91 to -2) | -77 (-94 to -32) | 41 (-63 to 430) | | 46 (-61 to 445) | -4 (-75 to 269) | |
| **Eastern Europe** | 848 (725 to 1,013) | 267 (232 to 330) | 273 (233 to 339) | 262 (219 to 334) | 27.75 (23.71 to 33.12) | 13.36 (11.63 to 16.55) | 14.35 (12.28 to 17.83) | 14.44 (12.04 to 18.40) | -48 (-64 to -22) | -52 (-65 to -30) | 8 (-27 to 58) | 7 (-26 to 53) | 1 (-33 to 50) | 76,103 (65,036 to 90,831) | 23,944 (20,837 to 29,653) | 24,458 (20,925 to 30,388) | 23,538 (19,622 to 30,007) | 2,489.26 (2,127.28 to 2,971.01) | 1,199.16 (1,043.54 to 1,485.07) | 1,288.22 (1,102.14 to 1,600.59) | 1,295.62 (1,080.08 to 1,651.70) | -48 (-64 to -22) | -52 (-65 to -30) | 8 (-27 to 58) | | 7 (-26 to 53) | 1 (-33 to 50) | |
| **Eastern Sub-Saharan Africa** | 8,039 (3,905 to 13,083) | 5,064 (2,727 to 7,527) | 4,742 (2,543 to 7,118) | 4,604 (2,478 to 6,812) | 97.36 (47.30 to 158.46) | 38.79 (20.89 to 57.66) | 36.15 (19.39 to 54.27) | 34.91 (18.79 to 51.65) | -64 (-88 to 9) | -60 (-87 to 22) | -10 (-67 to 147) | -7 (-66 to 160) | -3 (-65 to 166) | 721,307 (350,457 to 1,174,021) | 454,310 (244,678 to 675,054) | 425,418 (228,208 to 638,340) | 413,069 (222,353 to 611,013) | 8,736.05 (4,244.54 to 14,219.06) | 3,480.13 (1,874.29 to 5,171.09) | 3,243.55 (1,739.94 to 4,866.94) | 3,132.39 (1,686.15 to 4,633.44) | -64 (-88 to 9) | -60 (-87 to 22) | -10 (-67 to 147) | | -7 (-66 to 160) | -3 (-65 to 166) | |
| **High-income Asia Pacific** | 362 (280 to 476) | 88 (63 to 118) | 80 (58 to 107) | 72 (51 to 95) | 18.55 (14.34 to 24.42) | 6.93 (4.96 to 9.33) | 6.56 (4.75 to 8.79) | 6.08 (4.31 to 8.01) | -67 (-82 to -44) | -63 (-80 to -35) | -12 (-54 to 62) | -5 (-49 to 77) | -7 (-51 to 69) | 32,445 (25,092 to 42,723) | 7,853 (5,615 to 10,576) | 7,163 (5,189 to 9,605) | 6,498 (4,602 to 8,557) | 1,664.18 (1,287.03 to 2,191.36) | 621.96 (444.66 to 837.55) | 588.38 (426.25 to 789.02) | 545.90 (386.58 to 718.91) | -67 (-82 to -44) | -63 (-80 to -35) | -12 (-54 to 62) | | -5 (-49 to 77) | -7 (-51 to 69) | |
| **High-income North America** | 5,522 (5,293 to 5,739) | 1,321 (1,214 to 1,432) | 1,247 (1,110 to 1,392) | 1,195 (1,013 to 1,376) | 122.92 (117.81 to 127.73) | 32.32 (29.68 to 35.03) | 30.94 (27.53 to 34.55) | 29.72 (25.19 to 34.22) | -76 (-80 to -71) | -74 (-77 to -70) | -8 (-28 to 15) | -4 (-21 to 16) | -4 (-27 to 24) | 495,525 (474,931 to 514,944) | 118,573 (108,907 to 128,526) | 111,915 (99,556 to 124,942) | 107,206 (90,869 to 123,472) | 11,029.97 (10,571.56 to 11,462.21) | 2,899.74 (2,663.36 to 3,143.15) | 2,776.59 (2,469.97 to 3,099.81) | 2,666.52 (2,260.16 to 3,071.11) | -76 (-80 to -71) | -74 (-77 to -70) | -8 (-28 to 15) | | -4 (-21 to 16) | -4 (-27 to 24) | |
| **North Africa and Middle East** | 10,204 (5,979 to 15,411) | 4,754 (2,811 to 7,026) | 4,288 (2,479 to 6,390) | 4,046 (2,319 to 6,084) | 97.22 (56.96 to 146.82) | 38.15 (22.56 to 56.39) | 35.38 (20.45 to 52.72) | 34.22 (19.61 to 51.45) | -65 (-87 to -10) | -61 (-85 to -1) | -10 (-65 to 128) | -7 (-64 to 134) | -3 (-63 to 152) | 915,563 (536,521 to 1,382,918) | 426,514 (252,219 to 630,347) | 384,761 (222,349 to 573,373) | 363,017 (208,093 to 545,949) | 8,723.08 (5,111.74 to 13,175.83) | 3,423.20 (2,024.31 to 5,059.17) | 3,174.53 (1,834.52 to 4,730.69) | 3,070.17 (1,759.92 to 4,617.29) | -65 (-87 to -10) | -61 (-85 to -1) | -10 (-65 to 128) | | -7 (-64 to 134) | -3 (-63 to 152) | |
| **Oceania** | 113 (40 to 215) | 142 (66 to 237) | 141 (68 to 236) | 140 (65 to 242) | 52.87 (18.51 to 100.44) | 35.67 (16.57 to 59.62) | 34.82 (16.81 to 58.21) | 33.99 (15.68 to 58.62) | -36 (-84 to 217) | -33 (-83 to 222) | -5 (-74 to 254) | -2 (-72 to 251) | -2 (-73 to 249) | 10,171 (3,561 to 19,317) | 12,725 (5,914 to 21,270) | 12,655 (6,110 to 21,155) | 12,574 (5,804 to 21,681) | 4,744.15 (1,661.01 to 9,010.22) | 3,200.15 (1,487.19 to 5,348.79) | 3,124.06 (1,508.39 to 5,222.47) | 3,049.25 (1,407.65 to 5,257.83) | -36 (-84 to 217) | -33 (-83 to 222) | -5 (-74 to 254) | | -2 (-72 to 251) | -2 (-73 to 249) | |
| **South Asia** | 27,641 (11,937 to 53,175) | 9,810 (5,131 to 14,984) | 7,816 (4,101 to 12,320) | 7,437 (3,957 to 11,679) | 85.42 (36.89 to 164.34) | 30.87 (16.15 to 47.16) | 24.98 (13.11 to 39.38) | 24.11 (12.83 to 37.87) | -72 (-92 to 3) | -64 (-90 to 28) | -22 (-73 to 135) | -19 (-72 to 144) | -3 (-67 to 189) | 2,480,553 (1,071,093 to 4,772,142) | 880,178 (460,329 to 1,344,287) | 701,284 (367,975 to 1,105,407) | 667,250 (355,049 to 1,047,731) | 7,666.10 (3,310.19 to 14,748.20) | 2,769.91 (1,448.65 to 4,230.46) | 2,241.46 (1,176.13 to 3,533.13) | 2,163.50 (1,151.21 to 3,397.17) | -72 (-92 to 3) | -64 (-90 to 28) | -22 (-73 to 135) | | -19 (-72 to 144) | -3 (-67 to 189) | |
| **Southeast Asia** | 4,712 (2,024 to 8,006) | 1,682 (753 to 2,531) | 1,592 (710 to 2,372) | 1,571 (683 to 2,349) | 39.69 (17.04 to 67.43) | 14.89 (6.66 to 22.41) | 14.25 (6.35 to 21.22) | 14.19 (6.16 to 21.21) | -64 (-91 to 25) | -62 (-90 to 32) | -5 (-73 to 218) | -4 (-72 to 219) | 0 (-71 to 234) | 423,071 (181,606 to 718,709) | 150,983 (67,567 to 227,221) | 142,965 (63,730 to 212,967) | 141,036 (61,256 to 210,894) | 3,563.34 (1,529.58 to 6,053.36) | 1,336.65 (598.17 to 2,011.58) | 1,278.95 (570.12 to 1,905.18) | 1,273.62 (553.17 to 1,904.46) | -64 (-91 to 25) | -62 (-90 to 32) | -5 (-73 to 218) | | -4 (-72 to 219) | 0 (-71 to 234) | |
| **Southern Latin America** | 553 (410 to 735) | 160 (128 to 202) | 126 (97 to 163) | 118 (87 to 160) | 53.76 (39.83 to 71.50) | 18.78 (14.97 to 23.75) | 15.80 (12.24 to 20.45) | 15.41 (11.28 to 20.84) | -71 (-84 to -48) | -65 (-79 to -40) | -18 (-53 to 39) | -16 (-48 to 37) | -2 (-45 to 70) | 49,605 (36,754 to 65,979) | 14,365 (11,448 to 18,164) | 11,289 (8,741 to 14,606) | 10,627 (7,777 to 14,370) | 4,824.15 (3,574.37 to 6,416.47) | 1,686.05 (1,343.60 to 2,131.87) | 1,418.52 (1,098.38 to 1,835.37) | 1,383.49 (1,012.55 to 1,870.88) | -71 (-84 to -48) | -65 (-79 to -40) | -18 (-53 to 39) | | -16 (-48 to 37) | -2 (-45 to 70) | |
| **Southern Sub-Saharan Africa** | 393 (151 to 873) | 311 (114 to 758) | 287 (108 to 693) | 287 (108 to 683) | 25.36 (9.77 to 56.35) | 18.97 (6.94 to 46.29) | 17.75 (6.70 to 42.91) | 18.03 (6.76 to 42.94) | -29 (-88 to 339) | -25 (-88 to 373) | -5 (-85 to 519) | -6 (-86 to 518) | 2 (-84 to 541) | 35,251 (13,588 to 78,307) | 27,883 (10,195 to 68,029) | 25,721 (9,702 to 62,151) | 25,747 (9,660 to 61,299) | 2,275.74 (877.22 to 5,055.39) | 1,702.30 (622.40 to 4,153.29) | 1,592.91 (600.86 to 3,848.99) | 1,617.99 (607.07 to 3,852.10) | -29 (-88 to 339) | -25 (-88 to 373) | -5 (-85 to 519) | | -6 (-86 to 518) | 2 (-84 to 541) | |
| **Tropical Latin America** | 197 (166 to 232) | 150 (119 to 190) | 125 (97 to 160) | 119 (92 to 150) | 6.04 (5.08 to 7.10) | 4.28 (3.38 to 5.43) | 3.60 (2.80 to 4.61) | 3.48 (2.68 to 4.40) | -42 (-62 to -13) | -29 (-52 to 7) | -19 (-51 to 30) | -16 (-48 to 36) | -4 (-42 to 57) | 17,693 (14,872 to 20,789) | 13,473 (10,643 to 17,091) | 11,202 (8,707 to 14,332) | 10,650 (8,215 to 13,490) | 542.24 (455.78 to 637.12) | 384.07 (303.41 to 487.22) | 323.24 (251.24 to 413.54) | 311.90 (240.57 to 395.07) | -42 (-62 to -13) | -29 (-52 to 7) | -19 (-51 to 30) | | -16 (-48 to 36) | -4 (-42 to 57) | |
| **Western Europe** | 4,290 (4,102 to 4,483) | 473 (424 to 526) | 418 (359 to 485) | 410 (343 to 489) | 93.66 (89.56 to 97.88) | 11.33 (10.16 to 12.59) | 10.17 (8.73 to 11.80) | 10.04 (8.41 to 11.97) | -89 (-91 to -87) | -88 (-90 to -86) | -11 (-33 to 18) | -10 (-31 to 16) | -1 (-29 to 37) | 384,914 (368,055 to 402,252) | 42,479 (38,092 to 47,196) | 37,545 (32,244 to 43,561) | 36,780 (30,829 to 43,856) | 8,403.49 (8,035.42 to 8,782.00) | 1,016.90 (911.88 to 1,129.81) | 912.82 (783.93 to 1,059.07) | 900.90 (755.15 to 1,074.23) | -89 (-91 to -87) | -88 (-90 to -86) | -11 (-33 to 18) | | -10 (-31 to 16) | -1 (-29 to 37) | |
| **Western Sub-Saharan Africa** | 6,005 (3,002 to 9,380) | 8,624 (4,289 to 13,247) | 8,090 (3,924 to 12,278) | 7,891 (3,862 to 11,893) | 72.59 (36.29 to 113.38) | 52.16 (25.94 to 80.12) | 48.38 (23.47 to 73.42) | 46.60 (22.80 to 70.22) | -36 (-80 to 93) | -28 (-77 to 121) | -11 (-72 to 171) | -7 (-71 to 183) | -4 (-69 to 199) | 538,818 (269,377 to 841,467) | 773,640 (384,793 to 1,188,459) | 725,674 (352,023 to 1,101,400) | 707,896 (346,509 to 1,066,856) | 6,513.22 (3,256.23 to 10,171.65) | 4,679.12 (2,327.30 to 7,188.02) | 4,339.40 (2,105.04 to 6,586.17) | 4,179.95 (2,046.05 to 6,299.52) | -36 (-80 to 93) | -28 (-77 to 121) | -11 (-72 to 171) | | -7 (-71 to 183) | -4 (-69 to 199) | |

*DALYs* Disability-Adjusted Life Years, *SIDS* sudden infant death syndrome, *UI* uncertainty interval.

**Supplementary Table 2. GBD country or territory-specific trends in SIDS deaths and DALYs from 1990 to 2021, with percentage change in rates**

| **Location** | **Deaths (95% UI)** | | | | | | | | | | | | | **DALYs (95% UI)** | | | | | | | | | | | | |
| --- | --- | --- | --- | --- | --- | --- | --- | --- | --- | --- | --- | --- | --- | --- | --- | --- | --- | --- | --- | --- | --- | --- | --- | --- | --- | --- |
|  | **Number** | | | | **Rate (per 100,000)** | | | | **Percentage change in rates (%)** | | | | | **Number** | | | | **Rate (per 100,000)** | | | | **Percentage change in rates (%)** | | | | |
|  | **1990** | **2019** | **2020** | **2021** | **1990** | **2019** | **2020** | **2021** | **From 1990 to 2021** | **From 1990 to 2019** | **From 2019 to 2021** | **From 2019 to 2020** | **From 2020 to 2021** | **1990** | **2019** | **2020** | **2021** | **1990** | **2019** | **2020** | **2021** | **From 1990 to 2021** | **From 1990 to 2019** | **From 2019 to 2021** | **From 2019 to 2020** | **From 2020 to 2021** |
| **High-income Asis Pacific** | | | | | | | | | | | | | | | | | | | | | | | | | | |
| **Brunei Darussalam** | 0.82 (0.07 to 1.79) | 0.51 (0.05 to 1.33) | 0.49 (0.05 to 1.27) | 0.47 (0.05 to 1.23) | 11.94 (1.01 to 25.93) | 8.37 (0.85 to 21.93) | 8.06 (0.82 to 20.84) | 7.81 (0.76 to 20.28) | -35 (-97 to 1,909) | -30 (-97 to 2,073) | -7 (-97 to 2,287) | -4 (-96 to 2,353) | -3 (-96 to 2,359) | 73.79 (6.24 to 160.28) | 45.65 (4.64 to 119.67) | 44.00 (4.50 to 113.71) | 42.60 (4.15 to 110.57) | 1,071.18 (90.55 to 2,326.69) | 750.73 (76.23 to 1,968.05) | 723.68 (73.99 to 1,870.18) | 701.10 (68.27 to 1,819.70) | -35 (-97 to 1,909) | -30 (-97 to 2,073) | -7 (-97 to 2,287) | -4 (-96 to 2,353) | -3 (-96 to 2,359) |
| **Japan** | 285.50 (261.53 to 312.02) | 63.77 (55.80 to 72.81) | 59.70 (49.85 to 70.31) | 55.59 (45.39 to 67.90) | 23.01 (21.08 to 25.15) | 7.09 (6.21 to 8.10) | 6.83 (5.70 to 8.04) | 6.46 (5.27 to 7.89) | -72 (-79 to -63) | -69 (-75 to -62) | -9 (-35 to 27) | -4 (-30 to 30) | -5 (-34 to 38) | 25,618.22 (23,466.67 to 27,997.75) | 5,722.47 (5,006.93 to 6,533.48) | 5,357.35 (4,473.42 to 6,309.16) | 4,988.10 (4,072.81 to 6,093.26) | 2,064.64 (1,891.24 to 2,256.41) | 636.44 (556.86 to 726.63) | 612.51 (511.45 to 721.34) | 579.45 (473.12 to 707.83) | -72 (-79 to -63) | -69 (-75 to -62) | -9 (-35 to 27) | -4 (-30 to 30) | -5 (-34 to 38) |
| **Republic of Korea** | 74.67 (5.99 to 186.71) | 23.12 (3.06 to 51.55) | 19.53 (2.60 to 44.54) | 16.24 (2.23 to 36.24) | 11.44 (0.92 to 28.60) | 7.71 (1.02 to 17.19) | 6.97 (0.93 to 15.89) | 6.05 (0.83 to 13.51) | -47 (-97 to 1,372) | -33 (-96 to 1,773) | -21 (-95 to 1,225) | -10 (-95 to 1,458) | -13 (-95 to 1,355) | 6,699.96 (537.56 to 16,753.95) | 2,074.67 (274.44 to 4,624.77) | 1,752.26 (233.56 to 3,996.81) | 1,457.41 (199.86 to 3,252.44) | 1,026.42 (82.35 to 2,566.67) | 691.73 (91.50 to 1,541.98) | 625.22 (83.34 to 1,426.10) | 543.24 (74.49 to 1,212.32) | -47 (-97 to 1,372) | -33 (-96 to 1,773) | -21 (-95 to 1,225) | -10 (-95 to 1,458) | -13 (-95 to 1,355) |
| **Singapore** | 0.60 (0.20 to 1.07) | 0.12 (0.08 to 0.18) | 0.10 (0.07 to 0.15) | 0.11 (0.07 to 0.17) | 1.21 (0.41 to 2.18) | 0.21 (0.14 to 0.31) | 0.18 (0.12 to 0.26) | 0.20 (0.12 to 0.30) | -84 (-94 to -27) | -83 (-94 to -26) | -3 (-60 to 124) | -13 (-62 to 95) | 11 (-53 to 159) | 53.41 (18.30 to 96.16) | 10.60 (6.98 to 15.88) | 9.01 (5.91 to 13.36) | 9.82 (6.13 to 14.97) | 108.58 (37.21 to 195.50) | 18.42 (12.14 to 27.60) | 15.99 (10.49 to 23.71) | 17.82 (11.12 to 27.15) | -84 (-94 to -27) | -83 (-94 to -26) | -3 (-60 to 124) | -13 (-62 to 95) | 11 (-53 to 159) |
| **Central Asia** | | | | | | | | | | | | | | | | | | | | | | | | | | |
| **Armenia** | 1.31 (0.59 to 2.14) | 0.55 (0.36 to 0.84) | 0.51 (0.32 to 0.78) | 0.47 (0.29 to 0.74) | 1.75 (0.79 to 2.85) | 1.46 (0.95 to 2.21) | 1.41 (0.88 to 2.14) | 1.35 (0.82 to 2.13) | -23 (-71 to 168) | -16 (-67 to 179) | -8 (-63 to 125) | -3 (-60 to 126) | -5 (-62 to 141) | 117.32 (53.30 to 191.50) | 49.58 (32.13 to 74.99) | 46.10 (28.75 to 69.89) | 42.13 (25.66 to 66.51) | 156.59 (71.13 to 255.59) | 131.13 (84.98 to 198.33) | 126.79 (79.07 to 192.22) | 120.90 (73.62 to 190.85) | -23 (-71 to 168) | -16 (-67 to 179) | -8 (-63 to 125) | -3 (-60 to 126) | -5 (-62 to 141) |
| **Azerbaijan** | 14.02 (4.16 to 37.23) | 12.27 (5.21 to 24.81) | 11.55 (4.96 to 24.46) | 11.01 (4.71 to 23.19) | 7.86 (2.33 to 20.89) | 8.58 (3.65 to 17.36) | 8.37 (3.59 to 17.73) | 8.25 (3.53 to 17.39) | 5 (-83 to 645) | 9 (-83 to 644) | -4 (-80 to 377) | -2 (-79 to 386) | -1 (-80 to 384) | 1,257.00 (373.37 to 3,339.02) | 1,100.83 (467.95 to 2,225.80) | 1,036.25 (444.83 to 2,194.10) | 987.67 (422.59 to 2,080.83) | 705.14 (209.45 to 1,873.08) | 770.18 (327.39 to 1,557.25) | 751.30 (322.51 to 1,590.77) | 740.47 (316.82 to 1,560.03) | 5 (-83 to 645) | 9 (-83 to 644) | -4 (-80 to 377) | -2 (-79 to 386) | -1 (-80 to 384) |
| **Georgia** | 0.01 (0.01 to 0.02) | 4.26 (2.89 to 5.93) | 3.94 (2.62 to 5.67) | 3.66 (2.35 to 5.43) | 0.01 (0.01 to 0.02) | 8.76 (5.94 to 12.19) | 8.42 (5.60 to 12.10) | 8.12 (5.21 to 12.03) | 61,704 (25,427 to 149,008) | 66,631 (28,987 to 150,894) | -7 (-57 to 103) | -4 (-54 to 104) | -4 (-57 to 115) | 1.02 (0.62 to 1.58) | 382.56 (259.27 to 531.89) | 354.01 (235.57 to 508.80) | 328.35 (210.83 to 486.85) | 1.18 (0.72 to 1.83) | 786.47 (533.01 to 1,093.48) | 755.69 (502.87 to 1,086.13) | 728.42 (467.71 to 1,080.03) | 61,704 (25,427 to 149,008) | 66,631 (28,987 to 150,894) | -7 (-57 to 103) | -4 (-54 to 104) | -4 (-57 to 115) |
| **Kazakhstan** | 90.03 (28.73 to 137.50) | 52.19 (34.17 to 75.70) | 50.41 (31.54 to 75.74) | 47.96 (29.33 to 76.65) | 24.50 (7.82 to 37.41) | 13.15 (8.61 to 19.08) | 12.44 (7.78 to 18.69) | 11.76 (7.19 to 18.80) | -52 (-81 to 140) | -46 (-77 to 144) | -11 (-62 to 118) | -5 (-59 to 117) | -5 (-62 to 142) | 8,074.88 (2,577.50 to 12,333.20) | 4,684.11 (3,066.23 to 6,793.64) | 4,524.43 (2,830.53 to 6,796.50) | 4,303.87 (2,632.01 to 6,878.55) | 2,197.10 (701.31 to 3,355.75) | 1,180.38 (772.68 to 1,711.97) | 1,116.57 (698.53 to 1,677.28) | 1,055.45 (645.46 to 1,686.85) | -52 (-81 to 140) | -46 (-77 to 144) | -11 (-62 to 118) | -5 (-59 to 117) | -5 (-62 to 142) |
| **Kyrgyz** | 18.44 (11.64 to 28.85) | 7.18 (3.88 to 12.93) | 6.81 (3.54 to 12.75) | 6.58 (3.25 to 12.82) | 14.31 (9.03 to 22.39) | 4.45 (2.41 to 8.02) | 4.31 (2.24 to 8.06) | 4.24 (2.10 to 8.26) | -70 (-91 to -9) | -69 (-89 to -11) | -5 (-74 to 243) | -3 (-72 to 235) | -1 (-74 to 270) | 1,653.73 (1,044.13 to 2,587.55) | 643.91 (348.57 to 1,160.47) | 611.37 (317.50 to 1,143.98) | 590.38 (291.64 to 1,150.11) | 1,283.28 (810.23 to 2,007.92) | 399.19 (216.09 to 719.43) | 386.35 (200.65 to 722.94) | 380.68 (188.05 to 741.59) | -70 (-91 to -9) | -69 (-89 to -11) | -5 (-74 to 243) | -3 (-72 to 235) | -1 (-74 to 270) |
| **Mongolia** | 5.29 (1.36 to 14.27) | 6.08 (2.25 to 13.46) | 5.24 (1.93 to 12.48) | 5.75 (2.13 to 12.94) | 7.60 (1.95 to 20.49) | 7.79 (2.88 to 17.22) | 6.81 (2.51 to 16.21) | 7.60 (2.81 to 17.09) | 0 (-86 to 775) | 2 (-86 to 781) | -2 (-84 to 493) | -13 (-85 to 463) | 12 (-83 to 581) | 474.58 (122.09 to 1,279.66) | 546.03 (202.02 to 1,207.45) | 470.54 (173.51 to 1,120.19) | 516.46 (191.13 to 1,161.54) | 681.51 (175.33 to 1,837.63) | 698.69 (258.50 to 1,545.01) | 610.99 (225.30 to 1,454.56) | 681.99 (252.39 to 1,533.83) | 0 (-86 to 775) | 2 (-86 to 781) | -2 (-84 to 493) | -13 (-85 to 463) | 12 (-83 to 581) |
| **Tajikistan** | 16.58 (5.80 to 38.51) | 34.14 (12.07 to 78.19) | 32.22 (12.11 to 75.49) | 31.97 (12.41 to 77.04) | 8.27 (2.89 to 19.21) | 12.37 (4.38 to 28.33) | 11.64 (4.37 to 27.27) | 11.54 (4.48 to 27.82) | 40 (-77 to 861) | 50 (-77 to 879) | -7 (-84 to 536) | -6 (-85 to 523) | -1 (-84 to 536) | 1,486.96 (520.62 to 3,452.53) | 3,062.30 (1,083.44 to 7,011.69) | 2,890.37 (1,086.00 to 6,770.44) | 2,867.97 (1,113.78 to 6,908.99) | 741.78 (259.71 to 1,722.31) | 1,109.70 (392.61 to 2,540.87) | 1,044.04 (392.28 to 2,445.58) | 1,035.48 (402.13 to 2,494.49) | 40 (-77 to 861) | 50 (-77 to 879) | -7 (-84 to 536) | -6 (-85 to 523) | -1 (-84 to 536) |
| **Turkmenistan** | 14.60 (3.75 to 22.68) | 11.31 (6.33 to 17.54) | 10.93 (6.10 to 17.35) | 10.58 (5.88 to 17.53) | 12.09 (3.11 to 18.78) | 10.34 (5.78 to 16.02) | 10.03 (5.60 to 15.92) | 9.77 (5.43 to 16.19) | -19 (-71 to 421) | -15 (-69 to 416) | -5 (-66 to 180) | -3 (-65 to 175) | -3 (-66 to 189) | 1,309.20 (336.58 to 2,034.23) | 1,014.99 (567.63 to 1,573.88) | 980.22 (547.27 to 1,556.02) | 949.36 (527.76 to 1,572.84) | 1,084.14 (278.72 to 1,684.54) | 927.18 (518.52 to 1,437.71) | 899.32 (502.10 to 1,427.60) | 876.49 (487.25 to 1,452.12) | -19 (-71 to 421) | -15 (-69 to 416) | -5 (-66 to 180) | -3 (-65 to 175) | -3 (-66 to 189) |
| **Uzbekistan** | 69.83 (22.26 to 106.85) | 67.83 (37.31 to 106.91) | 66.22 (35.82 to 105.94) | 64.96 (35.75 to 103.83) | 10.13 (3.23 to 15.49) | 8.74 (4.81 to 13.78) | 8.45 (4.57 to 13.52) | 8.29 (4.56 to 13.25) | -18 (-71 to 310) | -14 (-69 to 327) | -5 (-67 to 175) | -3 (-67 to 181) | -2 (-66 to 190) | 6,262.69 (1,996.85 to 9,583.29) | 6,085.24 (3,348.53 to 9,590.17) | 5,941.42 (3,214.93 to 9,503.47) | 5,827.85 (3,208.01 to 9,314.13) | 908.07 (289.54 to 1,389.55) | 784.45 (431.66 to 1,236.28) | 757.98 (410.15 to 1,212.42) | 743.56 (409.30 to 1,188.37) | -18 (-71 to 310) | -14 (-69 to 327) | -5 (-67 to 175) | -3 (-67 to 181) | -2 (-66 to 190) |
| **East Asia** | | | | | | | | | | | | | | | | | | | | | | | | | | |
| **China** | 3,298.08 (1,775.57 to 5,291.78) | 469.07 (195.24 to 780.18) | 625.26 (260.77 to 1,045.71) | 532.09 (221.08 to 904.11) | 14.73 (7.93 to 23.64) | 3.19 (1.33 to 5.31) | 4.84 (2.02 to 8.09) | 4.63 (1.92 to 7.87) | -69 (-92 to -1) | -78 (-94 to -33) | 45 (-64 to 492) | 51 (-62 to 508) | -4 (-76 to 290) | 295,849.40 (159,278.78 to 474,653.71) | 42,093.74 (17,521.55 to 70,008.86) | 56,102.21 (23,399.82 to 93,806.29) | 47,742.43 (19,837.99 to 81,110.04) | 1,321.64 (711.54 to 2,120.41) | 286.70 (119.34 to 476.82) | 434.18 (181.09 to 725.98) | 415.55 (172.67 to 705.98) | -69 (-92 to -1) | -78 (-94 to -33) | 45 (-64 to 492) | 51 (-62 to 508) | -4 (-76 to 290) |
| **Democratic People's Republic of Korea** | 65.13 (22.31 to 155.21) | 31.30 (14.42 to 60.92) | 29.24 (12.86 to 57.86) | 27.38 (12.02 to 54.55) | 12.51 (4.28 to 29.81) | 10.03 (4.62 to 19.52) | 9.65 (4.24 to 19.08) | 9.30 (4.08 to 18.54) | -26 (-86 to 333) | -20 (-85 to 355) | -7 (-79 to 301) | -4 (-78 to 313) | -4 (-79 to 337) | 5,842.82 (2,002.18 to 13,920.50) | 2,808.76 (1,294.04 to 5,467.30) | 2,624.38 (1,154.03 to 5,191.50) | 2,456.90 (1,078.67 to 4,895.53) | 1,122.17 (384.54 to 2,673.56) | 899.79 (414.55 to 1,751.46) | 865.58 (380.62 to 1,712.28) | 835.00 (366.60 to 1,663.79) | -26 (-86 to 333) | -20 (-85 to 355) | -7 (-79 to 301) | -4 (-78 to 313) | -4 (-79 to 337) |
| **Taiwan (Province of China)** | 114.96 (98.15 to 133.31) | 22.33 (16.25 to 29.07) | 19.83 (14.26 to 26.91) | 18.94 (13.36 to 25.94) | 35.72 (30.50 to 41.42) | 12.68 (9.23 to 16.51) | 11.91 (8.57 to 16.16) | 11.91 (8.40 to 16.31) | -67 (-80 to -47) | -65 (-78 to -46) | -6 (-49 to 77) | -6 (-48 to 75) | 0 (-48 to 90) | 10,314.76 (8,805.63 to 11,961.63) | 2,003.74 (1,458.49 to 2,609.32) | 1,779.59 (1,280.08 to 2,414.87) | 1,699.82 (1,198.68 to 2,327.99) | 3,204.77 (2,735.89 to 3,716.45) | 1,137.73 (828.14 to 1,481.59) | 1,068.68 (768.71 to 1,450.18) | 1,069.04 (753.86 to 1,464.10) | -67 (-80 to -47) | -65 (-78 to -46) | -6 (-49 to 77) | -6 (-48 to 75) | 0 (-48 to 90) |
| **South Asis** | | | | | | | | | | | | | | | | | | | | | | | | | | |
| **Bangladesh** | 5,129.90 (1,996.08 to 12,232.06) | 1,005.91 (381.25 to 2,067.18) | 907.62 (343.66 to 1,897.14) | 821.61 (308.46 to 1,734.15) | 129.47 (50.38 to 308.71) | 34.42 (13.04 to 70.73) | 31.92 (12.09 to 66.73) | 29.67 (11.14 to 62.62) | -77 (-96 to 24) | -73 (-96 to 40) | -14 (-84 to 380) | -7 (-83 to 411) | -7 (-83 to 418) | 460,354.06 (179,105.35 to 1,097,610.64) | 90,253.42 (34,203.82 to 185,414.52) | 81,435.86 (30,834.39 to 170,162.58) | 73,719.13 (27,676.89 to 155,588.76) | 11,618.17 (4,520.16 to 27,700.91) | 3,088.06 (1,170.30 to 6,344.04) | 2,864.45 (1,084.58 to 5,985.36) | 2,662.03 (999.43 to 5,618.39) | -77 (-96 to 24) | -73 (-96 to 40) | -14 (-84 to 380) | -7 (-83 to 411) | -7 (-83 to 418) |
| **Bhutan** | 15.57 (5.29 to 38.41) | 2.56 (1.09 to 4.84) | 2.43 (1.08 to 4.56) | 2.32 (1.03 to 4.46) | 74.71 (25.41 to 184.35) | 20.03 (8.51 to 37.92) | 19.50 (8.65 to 36.59) | 18.96 (8.46 to 36.53) | -75 (-95 to 44) | -73 (-95 to 49) | -5 (-78 to 329) | -3 (-77 to 330) | -3 (-77 to 322) | 1,396.77 (475.03 to 3,446.78) | 229.42 (97.47 to 434.51) | 218.20 (96.87 to 409.45) | 207.78 (92.75 to 400.41) | 6,704.62 (2,280.21 to 16,544.84) | 1,796.77 (763.34 to 3,402.97) | 1,749.18 (776.54 to 3,282.26) | 1,700.84 (759.25 to 3,277.60) | -75 (-95 to 44) | -73 (-95 to 49) | -5 (-78 to 329) | -3 (-77 to 330) | -3 (-77 to 322) |
| **India** | 15,416.22 (7,194.56 to 28,221.13) | 5,599.76 (2,681.21 to 9,579.75) | 4,492.10 (2,152.33 to 7,565.67) | 4,250.65 (2,083.97 to 7,002.21) | 65.06 (30.36 to 119.11) | 25.18 (12.05 to 43.07) | 20.58 (9.86 to 34.67) | 19.82 (9.72 to 32.65) | -70 (-92 to 8) | -61 (-90 to 42) | -21 (-77 to 171) | -18 (-77 to 188) | -4 (-72 to 231) | 1,383,519.07 (645,646.93 to 2,532,935.17) | 502,464.71 (240,595.77 to 859,474.21) | 403,059.18 (193,144.29 to 678,728.10) | 381,394.89 (187,003.25 to 628,218.34) | 5,839.18 (2,724.97 to 10,690.31) | 2,258.96 (1,081.66 to 3,863.99) | 1,846.90 (885.03 to 3,110.08) | 1,778.32 (871.94 to 2,929.18) | -70 (-92 to 8) | -61 (-90 to 42) | -21 (-77 to 171) | -18 (-77 to 188) | -4 (-72 to 231) |
| **Nepal** | 792.61 (297.63 to 1,814.60) | 160.73 (79.24 to 297.66) | 155.78 (77.72 to 292.75) | 151.72 (74.91 to 282.02) | 108.27 (40.66 to 247.88) | 25.59 (12.62 to 47.40) | 24.80 (12.38 to 46.61) | 24.18 (11.94 to 44.95) | -78 (-95 to 11) | -76 (-95 to 17) | -6 (-75 to 256) | -3 (-74 to 270) | -3 (-74 to 263) | 71,128.82 (26,708.79 to 162,848.91) | 14,422.65 (7,109.57 to 26,709.26) | 13,978.68 (6,974.77 to 26,271.86) | 13,613.88 (6,721.21 to 25,309.45) | 9,716.50 (3,648.54 to 22,245.86) | 2,296.49 (1,132.04 to 4,252.86) | 2,225.78 (1,110.57 to 4,183.18) | 2,169.89 (1,071.28 to 4,034.02) | -78 (-95 to 11) | -76 (-95 to 17) | -6 (-75 to 256) | -3 (-74 to 270) | -3 (-74 to 263) |
| **Pakistan** | 6,286.52 (2,180.61 to 12,894.74) | 3,040.87 (1,487.32 to 5,239.75) | 2,257.99 (1,001.78 to 4,149.52) | 2,210.30 (996.33 to 4,145.66) | 159.21 (55.23 to 326.58) | 50.94 (24.91 to 87.77) | 37.76 (16.75 to 69.39) | 36.93 (16.65 to 69.26) | -77 (-95 to 25) | -68 (-92 to 59) | -28 (-81 to 178) | -26 (-81 to 178) | -2 (-76 to 313) | 564,154.28 (195,671.27 to 1,157,154.06) | 272,807.36 (133,452.80 to 470,100.78) | 202,591.84 (89,894.37 to 372,287.62) | 198,314.80 (89,410.51 to 371,961.65) | 14,287.91 (4,955.62 to 29,306.38) | 4,569.83 (2,235.49 to 7,874.72) | 3,387.86 (1,503.27 to 6,225.61) | 3,313.24 (1,493.78 to 6,214.36) | -77 (-95 to 25) | -68 (-92 to 59) | -28 (-81 to 178) | -26 (-81 to 178) | -2 (-76 to 313) |
| **Southeast Asia** | | | | | | | | | | | | | | | | | | | | | | | | | | |
| **Cambodia** | 250.67 (96.68 to 501.80) | 57.53 (20.94 to 126.70) | 57.29 (22.05 to 122.33) | 57.21 (23.05 to 121.64) | 62.89 (24.26 to 125.90) | 16.19 (5.89 to 35.66) | 16.24 (6.25 to 34.68) | 16.37 (6.59 to 34.80) | -74 (-95 to 43) | -74 (-95 to 47) | 1 (-82 to 490) | 0 (-82 to 488) | 1 (-81 to 457) | 22,506.28 (8,680.24 to 45,042.13) | 5,164.51 (1,880.54 to 11,370.88) | 5,142.79 (1,979.53 to 10,979.57) | 5,136.27 (2,069.33 to 10,916.77) | 5,646.56 (2,177.77 to 11,300.53) | 1,453.61 (529.30 to 3,200.46) | 1,457.91 (561.17 to 3,112.56) | 1,469.37 (591.99 to 3,123.05) | -74 (-95 to 43) | -74 (-95 to 47) | 1 (-82 to 490) | 0 (-82 to 488) | 1 (-81 to 457) |
| **Indonesia** | 2,225.34 (896.46 to 4,131.72) | 652.10 (294.61 to 1,058.24) | 618.52 (267.61 to 1,013.02) | 617.38 (267.12 to 1,005.09) | 49.59 (19.98 to 92.08) | 14.84 (6.70 to 24.08) | 14.17 (6.13 to 23.21) | 14.24 (6.16 to 23.18) | -71 (-93 to 16) | -70 (-93 to 21) | -4 (-74 to 246) | -4 (-75 to 246) | 0 (-73 to 278) | 199,819.32 (80,511.21 to 370,907.12) | 58,543.86 (26,449.68 to 95,012.20) | 55,529.16 (24,026.81 to 90,964.57) | 55,428.23 (23,981.01 to 90,205.50) | 4,453.14 (1,794.26 to 8,265.98) | 1,332.17 (601.86 to 2,162.01) | 1,272.45 (550.57 to 2,084.45) | 1,278.18 (553.00 to 2,080.14) | -71 (-93 to 16) | -70 (-93 to 21) | -4 (-74 to 246) | -4 (-75 to 246) | 0 (-73 to 278) |
| **Lao People's Democratic Republic** | 118.26 (42.02 to 249.06) | 48.59 (18.39 to 95.98) | 47.59 (18.24 to 93.12) | 46.44 (17.62 to 90.07) | 75.10 (26.69 to 158.17) | 28.42 (10.76 to 56.13) | 27.85 (10.67 to 54.50) | 27.24 (10.33 to 52.83) | -64 (-93 to 98) | -62 (-93 to 110) | -4 (-82 to 391) | -2 (-81 to 406) | -2 (-81 to 395) | 10,617.53 (3,773.78 to 22,362.87) | 4,362.38 (1,651.46 to 8,615.42) | 4,272.59 (1,637.31 to 8,356.69) | 4,168.73 (1,581.72 to 8,083.44) | 6,742.88 (2,396.62 to 14,202.00) | 2,550.95 (965.71 to 5,037.95) | 2,500.49 (958.22 to 4,890.68) | 2,445.17 (927.76 to 4,741.34) | -64 (-93 to 98) | -62 (-93 to 110) | -4 (-82 to 391) | -2 (-81 to 406) | -2 (-81 to 395) |
| **Malaysia** | 50.86 (20.85 to 99.97) | 33.42 (12.51 to 68.29) | 29.20 (10.28 to 59.13) | 29.36 (10.23 to 58.56) | 10.50 (4.31 to 20.64) | 6.88 (2.57 to 14.05) | 6.11 (2.15 to 12.37) | 6.20 (2.16 to 12.36) | -41 (-90 to 187) | -35 (-88 to 226) | -10 (-85 to 380) | -11 (-85 to 381) | 1 (-83 to 475) | 4,565.98 (1,871.85 to 8,972.36) | 3,000.39 (1,122.53 to 6,130.64) | 2,621.66 (923.01 to 5,307.72) | 2,636.08 (918.53 to 5,256.49) | 942.86 (386.53 to 1,852.75) | 617.51 (231.03 to 1,261.75) | 548.51 (193.12 to 1,110.50) | 556.50 (193.91 to 1,109.69) | -41 (-90 to 187) | -35 (-88 to 226) | -10 (-85 to 380) | -11 (-85 to 381) | 1 (-83 to 475) |
| **Maldives** | 1.67 (0.74 to 3.06) | 0.50 (0.20 to 0.88) | 0.44 (0.18 to 0.80) | 0.45 (0.18 to 0.83) | 19.59 (8.68 to 35.75) | 7.85 (3.18 to 13.94) | 7.21 (2.99 to 13.06) | 7.46 (3.02 to 13.83) | -62 (-92 to 59) | -60 (-91 to 61) | -5 (-78 to 335) | -8 (-79 to 311) | 3 (-77 to 363) | 150.25 (66.55 to 274.29) | 44.48 (18.01 to 78.96) | 39.86 (16.53 to 72.20) | 40.20 (16.25 to 74.51) | 1,757.99 (778.69 to 3,209.28) | 704.80 (285.39 to 1,251.14) | 647.17 (268.31 to 1,172.26) | 669.57 (270.68 to 1,241.21) | -62 (-92 to 59) | -60 (-91 to 61) | -5 (-78 to 335) | -8 (-79 to 311) | 3 (-77 to 363) |
| **Mauritius** | 2.20 (1.61 to 2.95) | 2.38 (1.75 to 3.21) | 2.19 (1.54 to 3.12) | 1.96 (1.34 to 2.77) | 9.85 (7.19 to 13.19) | 18.52 (13.58 to 24.96) | 17.12 (12.01 to 24.44) | 15.50 (10.60 to 21.98) | 57 (-20 to 206) | 88 (3 to 247) | -16 (-58 to 62) | -8 (-52 to 80) | -9 (-57 to 83) | 197.56 (144.32 to 264.73) | 213.97 (156.95 to 288.37) | 196.38 (137.79 to 280.37) | 175.52 (120.00 to 248.85) | 883.46 (645.40 to 1,183.86) | 1,662.33 (1,219.31 to 2,240.31) | 1,536.77 (1,078.31 to 2,194.06) | 1,391.87 (951.59 to 1,973.39) | 57 (-20 to 206) | 88 (3 to 247) | -16 (-58 to 62) | -8 (-52 to 80) | -9 (-57 to 83) |
| **Myanmar** | 645.34 (248.70 to 1,214.87) | 313.01 (130.49 to 573.49) | 299.56 (123.19 to 544.51) | 289.49 (115.96 to 532.49) | 61.29 (23.62 to 115.38) | 29.81 (12.42 to 54.61) | 28.58 (11.75 to 51.95) | 27.68 (11.09 to 50.91) | -55 (-90 to 116) | -51 (-89 to 131) | -7 (-80 to 310) | -4 (-78 to 318) | -3 (-79 to 333) | 57,940.07 (22,337.64 to 109,050.94) | 28,097.47 (11,715.46 to 51,478.09) | 26,890.11 (11,060.11 to 48,866.55) | 25,986.57 (10,411.08 to 47,789.70) | 5,502.68 (2,121.45 to 10,356.78) | 2,675.46 (1,115.56 to 4,901.78) | 2,565.65 (1,055.27 to 4,662.47) | 2,484.61 (995.42 to 4,569.24) | -55 (-90 to 116) | -51 (-89 to 131) | -7 (-80 to 310) | -4 (-78 to 318) | -3 (-79 to 333) |
| **Philippines** | 654.89 (222.71 to 1,164.62) | 310.15 (137.10 to 511.49) | 283.80 (118.60 to 466.98) | 283.52 (122.99 to 473.56) | 33.55 (11.41 to 59.67) | 13.66 (6.04 to 22.53) | 12.62 (5.27 to 20.77) | 12.69 (5.51 to 21.20) | -62 (-91 to 86) | -59 (-90 to 97) | -7 (-76 to 251) | -8 (-77 to 244) | 1 (-73 to 302) | 58,779.83 (19,974.70 to 104,544.35) | 27,841.13 (12,306.12 to 45,918.10) | 25,475.84 (10,643.65 to 41,921.28) | 25,450.84 (11,039.48 to 42,513.32) | 3,011.45 (1,023.36 to 5,356.09) | 1,226.32 (542.05 to 2,022.55) | 1,132.83 (473.29 to 1,864.11) | 1,139.25 (494.16 to 1,903.01) | -62 (-91 to 86) | -59 (-90 to 97) | -7 (-76 to 251) | -8 (-77 to 244) | 1 (-73 to 302) |
| **Seychelles** | 0.13 (0.05 to 0.23) | 0.12 (0.05 to 0.22) | 0.12 (0.05 to 0.22) | 0.12 (0.05 to 0.23) | 7.90 (3.00 to 14.12) | 7.54 (3.45 to 13.85) | 7.42 (3.22 to 13.67) | 7.58 (3.27 to 14.36) | -4 (-77 to 379) | -5 (-76 to 362) | 0 (-76 to 317) | -2 (-77 to 297) | 2 (-76 to 346) | 11.52 (4.37 to 20.59) | 10.77 (4.92 to 19.77) | 10.52 (4.56 to 19.37) | 10.67 (4.60 to 20.21) | 709.49 (269.25 to 1,268.24) | 677.18 (309.29 to 1,243.07) | 666.55 (288.75 to 1,227.39) | 680.37 (293.44 to 1,289.11) | -4 (-77 to 379) | -5 (-76 to 362) | 0 (-76 to 317) | -2 (-77 to 297) | 2 (-76 to 346) |
| **Sri Lanka** | 26.02 (11.09 to 46.52) | 18.28 (6.06 to 35.48) | 17.51 (6.00 to 33.90) | 16.32 (5.25 to 31.48) | 7.39 (3.15 to 13.21) | 5.81 (1.93 to 11.28) | 5.69 (1.95 to 11.02) | 5.42 (1.74 to 10.46) | -27 (-87 to 232) | -21 (-85 to 258) | -7 (-85 to 443) | -2 (-83 to 472) | -5 (-84 to 436) | 2,336.24 (994.95 to 4,174.84) | 1,641.75 (544.52 to 3,185.14) | 1,572.11 (538.99 to 3,043.97) | 1,465.04 (471.29 to 2,827.21) | 663.41 (282.53 to 1,185.51) | 521.77 (173.05 to 1,012.28) | 511.12 (175.23 to 989.64) | 486.60 (156.54 to 939.03) | -27 (-87 to 232) | -21 (-85 to 258) | -7 (-85 to 443) | -2 (-83 to 472) | -5 (-84 to 436) |
| **Thailand** | 152.32 (66.83 to 262.36) | 42.61 (20.64 to 74.73) | 40.42 (18.80 to 70.46) | 39.03 (17.45 to 69.11) | 14.90 (6.54 to 25.67) | 7.52 (3.64 to 13.19) | 7.34 (3.41 to 12.79) | 7.27 (3.25 to 12.87) | -51 (-87 to 97) | -50 (-86 to 102) | -3 (-75 to 253) | -2 (-74 to 251) | -1 (-75 to 277) | 13,675.74 (6,002.04 to 23,560.27) | 3,825.58 (1,852.70 to 6,708.38) | 3,628.59 (1,687.38 to 6,324.21) | 3,504.47 (1,565.90 to 6,203.80) | 1,337.96 (587.21 to 2,305.00) | 675.34 (327.06 to 1,184.25) | 658.94 (306.42 to 1,148.45) | 652.89 (291.73 to 1,155.78) | -51 (-87 to 97) | -50 (-86 to 102) | -3 (-75 to 253) | -2 (-74 to 251) | -1 (-75 to 277) |
| **Timor-Leste** | 21.02 (8.26 to 52.15) | 10.41 (4.33 to 17.77) | 10.59 (4.33 to 17.99) | 10.89 (4.59 to 18.95) | 64.10 (25.18 to 159.05) | 27.86 (11.58 to 47.56) | 27.67 (11.31 to 47.03) | 27.74 (11.69 to 48.27) | -57 (-93 to 92) | -57 (-93 to 89) | 0 (-75 to 317) | -1 (-76 to 306) | 0 (-75 to 327) | 1,886.50 (741.35 to 4,681.16) | 934.47 (388.46 to 1,594.99) | 950.45 (388.43 to 1,614.85) | 977.51 (411.71 to 1,700.89) | 5,753.88 (2,261.15 to 14,277.71) | 2,500.95 (1,039.66 to 4,268.75) | 2,484.08 (1,015.19 to 4,220.55) | 2,490.36 (1,048.89 to 4,333.26) | -57 (-93 to 92) | -57 (-93 to 89) | 0 (-75 to 317) | -1 (-76 to 306) | 0 (-75 to 327) |
| **Viet Nam** | 556.59 (212.83 to 979.84) | 190.35 (74.93 to 362.41) | 183.04 (68.92 to 350.68) | 176.63 (65.35 to 351.28) | 29.55 (11.30 to 52.01) | 11.80 (4.64 to 22.46) | 11.56 (4.35 to 22.15) | 11.39 (4.21 to 22.65) | -61 (-92 to 100) | -60 (-91 to 99) | -3 (-81 to 388) | -2 (-81 to 377) | -1 (-81 to 420) | 49,972.06 (19,104.98 to 87,996.03) | 17,091.53 (6,726.80 to 32,540.34) | 16,435.14 (6,187.23 to 31,487.83) | 15,859.63 (5,866.81 to 31,540.79) | 2,652.73 (1,014.18 to 4,671.21) | 1,059.39 (416.95 to 2,016.96) | 1,038.10 (390.81 to 1,988.88) | 1,022.66 (378.30 to 2,033.81) | -61 (-92 to 100) | -60 (-91 to 99) | -3 (-81 to 388) | -2 (-81 to 377) | -1 (-81 to 420) |
| **Australasia** | | | | | | | | | | | | | | | | | | | | | | | | | | |
| **Australia** | 406.05 (374.38 to 441.06) | 33.26 (25.50 to 43.11) | 30.76 (22.28 to 41.14) | 25.32 (17.57 to 35.10) | 161.02 (148.46 to 174.90) | 11.18 (8.58 to 14.50) | 10.51 (7.62 to 14.06) | 8.64 (6.00 to 11.98) | -95 (-97 to -92) | -93 (-95 to -90) | -23 (-59 to 40) | -6 (-47 to 64) | -18 (-57 to 57) | 36,431.67 (33,590.25 to 39,574.06) | 2,984.17 (2,288.16 to 3,869.17) | 2,760.57 (1,999.20 to 3,692.10) | 2,272.50 (1,576.10 to 3,149.61) | 14,446.95 (13,320.19 to 15,693.06) | 1,003.65 (769.56 to 1,301.29) | 943.56 (683.32 to 1,261.95) | 775.78 (538.04 to 1,075.20) | -95 (-97 to -92) | -93 (-95 to -90) | -23 (-59 to 40) | -6 (-47 to 64) | -18 (-57 to 57) |
| **New Zealand** | 137.07 (125.69 to 148.45) | 24.31 (19.68 to 29.26) | 20.54 (16.56 to 24.49) | 21.99 (17.04 to 27.41) | 231.64 (212.42 to 250.88) | 40.52 (32.80 to 48.76) | 33.75 (27.21 to 40.25) | 36.12 (27.99 to 45.02) | -84 (-89 to -79) | -83 (-87 to -77) | -11 (-43 to 37) | -17 (-44 to 23) | 7 (-30 to 65) | 12,295.67 (11,274.52 to 13,316.68) | 2,181.24 (1,765.82 to 2,624.86) | 1,842.57 (1,485.43 to 2,197.32) | 1,973.48 (1,529.26 to 2,459.66) | 20,779.89 (19,054.12 to 22,505.42) | 3,635.36 (2,942.99 to 4,374.70) | 3,028.53 (2,441.52 to 3,611.61) | 3,241.22 (2,511.64 to 4,039.71) | -84 (-89 to -79) | -83 (-87 to -77) | -11 (-43 to 37) | -17 (-44 to 23) | 7 (-30 to 65) |
| **Caribbean** | | | | | | | | | | | | | | | | | | | | | | | | | | |
| **Antigua and Barbuda** | 0.00 (0.00 to 0.00) | 0.00 (0.00 to 0.00) | 0.00 (0.00 to 0.00) | 0.00 (0.00 to 0.00) | 0.00 (0.00 to 0.00) | 0.00 (0.00 to 0.00) | 0.00 (0.00 to 0.00) | 0.00 (0.00 to 0.00) | -42 (-70 to 9) | -36 (-67 to 22) | -10 (-54 to 80) | -5 (-53 to 91) | -5 (-52 to 89) | 0.00 (0.00 to 0.00) | 0.00 (0.00 to 0.00) | 0.00 (0.00 to 0.00) | 0.00 (0.00 to 0.00) | 0.03 (0.02 to 0.04) | 0.02 (0.01 to 0.02) | 0.02 (0.01 to 0.02) | 0.02 (0.01 to 0.02) | -42 (-70 to 9) | -36 (-67 to 22) | -10 (-54 to 80) | -5 (-53 to 91) | -5 (-52 to 89) |
| **Bahamas** | 1.05 (0.75 to 1.39) | 0.60 (0.40 to 0.85) | 0.57 (0.37 to 0.81) | 0.56 (0.38 to 0.80) | 19.91 (14.34 to 26.47) | 14.96 (9.95 to 21.15) | 14.28 (9.35 to 20.24) | 14.16 (9.48 to 20.21) | -29 (-64 to 41) | -25 (-62 to 47) | -5 (-55 to 103) | -5 (-56 to 103) | -1 (-53 to 116) | 93.99 (67.71 to 124.91) | 54.18 (36.05 to 76.61) | 51.19 (33.51 to 72.54) | 50.33 (33.71 to 71.86) | 1,787.18 (1,287.34 to 2,375.08) | 1,342.60 (893.19 to 1,898.42) | 1,281.32 (838.75 to 1,816.01) | 1,270.48 (850.87 to 1,814.00) | -29 (-64 to 41) | -25 (-62 to 47) | -5 (-55 to 103) | -5 (-56 to 103) | -1 (-53 to 116) |
| **Barbados** | 0.54 (0.40 to 0.74) | 0.15 (0.10 to 0.21) | 0.14 (0.09 to 0.21) | 0.14 (0.09 to 0.21) | 13.28 (9.75 to 18.35) | 5.43 (3.70 to 7.85) | 5.34 (3.56 to 7.77) | 5.31 (3.52 to 7.98) | -60 (-81 to -18) | -59 (-80 to -19) | -2 (-55 to 115) | -2 (-55 to 110) | -1 (-55 to 124) | 48.37 (35.51 to 66.84) | 13.15 (8.96 to 19.01) | 12.70 (8.46 to 18.48) | 12.43 (8.24 to 18.69) | 1,191.87 (875.07 to 1,647.12) | 487.43 (332.34 to 704.96) | 479.10 (319.30 to 697.33) | 476.51 (315.80 to 716.08) | -60 (-81 to -18) | -59 (-80 to -19) | -2 (-55 to 115) | -2 (-55 to 110) | -1 (-55 to 124) |
| **Belize** | 4.90 (3.58 to 6.84) | 1.14 (0.79 to 1.57) | 1.11 (0.78 to 1.57) | 1.08 (0.74 to 1.58) | 80.18 (58.70 to 112.05) | 15.17 (10.49 to 20.96) | 14.73 (10.36 to 20.87) | 14.36 (9.85 to 20.96) | -82 (-91 to -64) | -81 (-91 to -64) | -5 (-53 to 100) | -3 (-51 to 99) | -3 (-53 to 102) | 439.35 (321.57 to 614.03) | 102.11 (70.62 to 141.08) | 99.19 (69.77 to 140.53) | 97.00 (66.53 to 141.60) | 7,194.81 (5,266.05 to 10,055.36) | 1,361.74 (941.84 to 1,881.45) | 1,322.09 (929.96 to 1,873.13) | 1,288.65 (883.89 to 1,881.18) | -82 (-91 to -64) | -81 (-91 to -64) | -5 (-53 to 100) | -3 (-51 to 99) | -3 (-53 to 102) |
| **Bermuda** | 0.32 (0.23 to 0.43) | 0.05 (0.03 to 0.07) | 0.05 (0.03 to 0.07) | 0.05 (0.03 to 0.07) | 36.54 (26.27 to 49.13) | 9.92 (6.61 to 14.17) | 9.65 (6.28 to 13.82) | 9.77 (6.81 to 13.73) | -73 (-86 to -48) | -73 (-87 to -46) | -2 (-52 to 108) | -3 (-56 to 109) | 1 (-51 to 119) | 28.82 (20.72 to 38.74) | 4.65 (3.10 to 6.64) | 4.39 (2.86 to 6.29) | 4.31 (3.00 to 6.05) | 3,279.49 (2,357.51 to 4,409.24) | 890.74 (593.41 to 1,271.87) | 865.94 (563.72 to 1,240.45) | 876.57 (611.13 to 1,231.96) | -73 (-86 to -48) | -73 (-87 to -46) | -2 (-52 to 108) | -3 (-56 to 109) | 1 (-51 to 119) |
| **Cuba** | 4.81 (3.80 to 6.11) | 0.66 (0.45 to 0.94) | 0.58 (0.39 to 0.82) | 0.51 (0.33 to 0.75) | 2.68 (2.12 to 3.41) | 0.60 (0.41 to 0.86) | 0.55 (0.37 to 0.78) | 0.51 (0.33 to 0.75) | -81 (-90 to -65) | -77 (-88 to -60) | -16 (-62 to 81) | -9 (-57 to 90) | -7 (-58 to 101) | 431.54 (340.72 to 548.24) | 59.49 (40.59 to 84.10) | 51.76 (34.95 to 73.85) | 45.96 (29.71 to 67.29) | 240.82 (190.13 to 305.94) | 54.28 (37.04 to 76.74) | 49.35 (33.32 to 70.40) | 45.68 (29.53 to 66.88) | -81 (-90 to -65) | -77 (-88 to -60) | -16 (-62 to 81) | -9 (-57 to 90) | -7 (-58 to 101) |
| **Dominica** | 0.10 (0.05 to 0.18) | 0.04 (0.02 to 0.08) | 0.04 (0.02 to 0.07) | 0.04 (0.02 to 0.08) | 5.33 (2.52 to 10.01) | 5.60 (2.37 to 11.34) | 5.61 (2.38 to 11.42) | 5.62 (2.36 to 12.01) | 5 (-76 to 377) | 5 (-76 to 350) | 0 (-79 to 406) | 0 (-79 to 381) | 0 (-79 to 404) | 8.76 (4.14 to 16.44) | 3.40 (1.44 to 6.88) | 3.30 (1.40 to 6.72) | 3.22 (1.35 to 6.88) | 478.64 (226.06 to 898.48) | 502.49 (212.86 to 1,017.95) | 503.26 (213.66 to 1,024.92) | 504.50 (211.80 to 1,077.61) | 5 (-76 to 377) | 5 (-76 to 350) | 0 (-79 to 406) | 0 (-79 to 381) | 0 (-79 to 404) |
| **Dominican Republic** | 17.73 (6.38 to 40.10) | 11.12 (5.04 to 19.92) | 10.59 (4.85 to 19.38) | 10.70 (4.82 to 20.11) | 8.24 (2.97 to 18.63) | 5.32 (2.41 to 9.52) | 5.06 (2.32 to 9.26) | 5.12 (2.31 to 9.62) | -38 (-88 to 224) | -35 (-87 to 221) | -4 (-76 to 300) | -5 (-76 to 285) | 1 (-75 to 315) | 1,591.31 (573.13 to 3,597.70) | 998.45 (452.02 to 1,788.03) | 950.91 (435.31 to 1,739.37) | 960.01 (432.82 to 1,804.62) | 739.15 (266.22 to 1,671.11) | 477.13 (216.00 to 854.44) | 454.16 (207.91 to 830.74) | 459.19 (207.03 to 863.19) | -38 (-88 to 224) | -35 (-87 to 221) | -4 (-76 to 300) | -5 (-76 to 285) | 1 (-75 to 315) |
| **Grenada** | 0.23 (0.16 to 0.32) | 0.00 (0.00 to 0.00) | 0.00 (0.00 to 0.00) | 0.00 (0.00 to 0.00) | 9.52 (6.81 to 13.21) | 0.03 (0.02 to 0.04) | 0.03 (0.02 to 0.04) | 0.02 (0.02 to 0.04) | -100 (-100 to -99) | -100 (-100 to -99) | -5 (-57 to 114) | -3 (-55 to 121) | -3 (-58 to 121) | 20.45 (14.63 to 28.38) | 0.03 (0.02 to 0.05) | 0.03 (0.02 to 0.05) | 0.03 (0.02 to 0.04) | 854.38 (611.21 to 1,185.25) | 2.33 (1.52 to 3.29) | 2.27 (1.47 to 3.35) | 2.21 (1.41 to 3.24) | -100 (-100 to -99) | -100 (-100 to -99) | -5 (-57 to 114) | -3 (-55 to 121) | -3 (-58 to 121) |
| **Guyana** | 3.16 (1.98 to 4.56) | 0.86 (0.52 to 1.29) | 0.81 (0.48 to 1.23) | 0.75 (0.45 to 1.15) | 12.54 (7.85 to 18.13) | 5.56 (3.35 to 8.32) | 5.33 (3.18 to 8.07) | 5.11 (3.07 to 7.80) | -59 (-83 to -1) | -56 (-82 to 6) | -8 (-63 to 133) | -4 (-62 to 141) | -4 (-62 to 146) | 283.42 (177.58 to 409.70) | 77.50 (46.67 to 115.95) | 72.67 (43.32 to 110.15) | 67.68 (40.70 to 103.38) | 1,125.38 (705.12 to 1,626.80) | 498.89 (300.44 to 746.43) | 477.89 (284.89 to 724.36) | 458.16 (275.52 to 699.87) | -59 (-83 to -1) | -56 (-82 to 6) | -8 (-63 to 133) | -4 (-62 to 141) | -4 (-62 to 146) |
| **Haiti** | 117.65 (47.24 to 283.79) | 88.92 (35.48 to 193.70) | 86.97 (34.26 to 178.49) | 86.36 (34.03 to 180.18) | 50.10 (20.12 to 120.85) | 27.50 (10.97 to 59.91) | 26.72 (10.53 to 54.83) | 26.37 (10.39 to 55.02) | -47 (-91 to 173) | -45 (-91 to 198) | -4 (-83 to 401) | -3 (-82 to 400) | -1 (-81 to 423) | 10,559.44 (4,238.57 to 25,470.28) | 7,980.69 (3,183.53 to 17,382.35) | 7,805.64 (3,075.33 to 16,021.48) | 7,750.45 (3,054.30 to 16,172.05) | 4,496.57 (1,804.93 to 10,846.12) | 2,468.18 (984.57 to 5,375.82) | 2,397.83 (944.72 to 4,921.66) | 2,366.50 (932.59 to 4,937.93) | -47 (-91 to 173) | -45 (-91 to 198) | -4 (-83 to 401) | -3 (-82 to 400) | -1 (-81 to 423) |
| **Jamaica** | 4.18 (3.18 to 5.49) | 0.76 (0.50 to 1.11) | 0.73 (0.46 to 1.11) | 0.68 (0.42 to 1.08) | 7.45 (5.66 to 9.79) | 2.22 (1.47 to 3.24) | 2.18 (1.38 to 3.34) | 2.09 (1.29 to 3.29) | -72 (-87 to -42) | -70 (-85 to -43) | -6 (-60 to 123) | -2 (-57 to 126) | -4 (-61 to 138) | 375.10 (285.07 to 493.01) | 67.93 (45.12 to 99.26) | 65.44 (41.34 to 100.05) | 61.36 (37.88 to 96.66) | 668.20 (507.82 to 878.25) | 199.19 (132.31 to 291.05) | 195.95 (123.79 to 299.61) | 187.44 (115.72 to 295.24) | -72 (-87 to -42) | -70 (-85 to -43) | -6 (-60 to 123) | -2 (-57 to 126) | -4 (-61 to 138) |
| **Puerto Rico** | 5.62 (4.25 to 7.26) | 0.00 (0.00 to 0.00) | 0.00 (0.00 to 0.00) | 0.00 (0.00 to 0.00) | 8.68 (6.57 to 11.21) | 0.00 (0.00 to 0.00) | 0.00 (0.00 to 0.00) | 0.00 (0.00 to 0.00) | -100 (-100 to -100) | -100 (-100 to -100) | -8 (-55 to 83) | -3 (-51 to 92) | -5 (-53 to 86) | 504.90 (381.91 to 651.58) | 0.05 (0.04 to 0.07) | 0.05 (0.03 to 0.07) | 0.04 (0.03 to 0.06) | 779.56 (589.66 to 1,006.03) | 0.25 (0.17 to 0.35) | 0.24 (0.17 to 0.33) | 0.23 (0.16 to 0.32) | -100 (-100 to -100) | -100 (-100 to -100) | -8 (-55 to 83) | -3 (-51 to 92) | -5 (-53 to 86) |
| **Saint Kitts and Nevis** | 0.16 (0.11 to 0.21) | 0.03 (0.02 to 0.05) | 0.03 (0.02 to 0.04) | 0.03 (0.02 to 0.04) | 17.16 (12.49 to 22.95) | 5.07 (3.42 to 7.49) | 4.79 (3.15 to 7.31) | 4.82 (3.08 to 7.55) | -72 (-87 to -40) | -70 (-85 to -40) | -5 (-59 to 121) | -6 (-58 to 114) | 1 (-58 to 140) | 14.13 (10.28 to 18.89) | 2.75 (1.86 to 4.07) | 2.54 (1.67 to 3.88) | 2.50 (1.60 to 3.92) | 1,540.32 (1,121.09 to 2,059.47) | 455.06 (306.68 to 671.96) | 429.83 (282.61 to 655.72) | 432.75 (276.27 to 677.46) | -72 (-87 to -40) | -70 (-85 to -40) | -5 (-59 to 121) | -6 (-58 to 114) | 1 (-58 to 140) |
| **Saint Lucia** | 1.29 (0.98 to 1.66) | 0.24 (0.16 to 0.36) | 0.22 (0.14 to 0.34) | 0.22 (0.14 to 0.33) | 36.69 (27.88 to 47.15) | 13.67 (8.83 to 20.24) | 12.89 (8.09 to 19.65) | 12.84 (8.16 to 19.59) | -65 (-83 to -30) | -63 (-81 to -27) | -6 (-60 to 122) | -6 (-60 to 123) | 0 (-58 to 142) | 115.64 (87.87 to 148.60) | 21.59 (13.94 to 31.97) | 19.86 (12.46 to 30.28) | 19.31 (12.28 to 29.47) | 3,293.02 (2,502.27 to 4,231.58) | 1,227.11 (792.06 to 1,816.87) | 1,156.48 (725.73 to 1,763.25) | 1,152.20 (732.41 to 1,757.84) | -65 (-83 to -30) | -63 (-81 to -27) | -6 (-60 to 122) | -6 (-60 to 123) | 0 (-58 to 142) |
| **Saint Vincent and the Grenadines** | 0.46 (0.33 to 0.61) | 0.03 (0.02 to 0.05) | 0.03 (0.02 to 0.04) | 0.03 (0.02 to 0.04) | 18.15 (12.96 to 24.41) | 2.29 (1.53 to 3.25) | 2.17 (1.43 to 3.22) | 2.21 (1.41 to 3.33) | -88 (-94 to -74) | -87 (-94 to -75) | -3 (-57 to 118) | -5 (-56 to 111) | 2 (-56 to 133) | 40.91 (29.22 to 55.02) | 2.93 (1.95 to 4.17) | 2.65 (1.75 to 3.93) | 2.59 (1.65 to 3.90) | 1,628.81 (1,163.37 to 2,190.45) | 205.38 (136.96 to 292.12) | 194.73 (128.59 to 289.26) | 198.70 (126.71 to 299.11) | -88 (-94 to -74) | -87 (-94 to -75) | -3 (-57 to 118) | -5 (-56 to 111) | 2 (-56 to 133) |
| **Suriname** | 0.90 (0.33 to 1.88) | 0.45 (0.21 to 0.86) | 0.42 (0.20 to 0.80) | 0.41 (0.19 to 0.77) | 10.05 (3.71 to 21.11) | 5.01 (2.36 to 9.50) | 4.73 (2.24 to 9.03) | 4.66 (2.18 to 8.82) | -54 (-90 to 138) | -50 (-89 to 156) | -7 (-77 to 274) | -6 (-76 to 284) | -2 (-76 to 294) | 80.40 (29.68 to 168.91) | 40.65 (19.10 to 77.02) | 37.80 (17.88 to 72.13) | 36.60 (17.12 to 69.25) | 901.81 (332.91 to 1,894.65) | 449.74 (211.35 to 852.24) | 424.80 (200.92 to 810.75) | 418.08 (195.58 to 791.01) | -54 (-90 to 138) | -50 (-89 to 156) | -7 (-77 to 274) | -6 (-76 to 284) | -2 (-76 to 294) |
| **Trinidad and Tobago** | 7.04 (5.23 to 9.35) | 1.22 (0.86 to 1.70) | 1.14 (0.78 to 1.64) | 1.10 (0.75 to 1.58) | 29.31 (21.76 to 38.92) | 7.68 (5.42 to 10.64) | 7.42 (5.10 to 10.63) | 7.43 (5.06 to 10.65) | -75 (-87 to -51) | -74 (-86 to -51) | -3 (-52 to 97) | -3 (-52 to 96) | 0 (-52 to 109) | 632.19 (469.48 to 839.44) | 109.86 (77.48 to 152.17) | 102.46 (70.45 to 146.83) | 99.04 (67.56 to 142.12) | 2,630.19 (1,953.27 to 3,492.44) | 689.70 (486.41 to 955.29) | 666.05 (457.97 to 954.45) | 666.49 (454.63 to 956.38) | -75 (-87 to -51) | -74 (-86 to -51) | -3 (-52 to 97) | -3 (-52 to 96) | 0 (-52 to 109) |
| **United States Virgin Islands** | 0.13 (0.06 to 0.23) | 0.02 (0.01 to 0.05) | 0.02 (0.01 to 0.04) | 0.02 (0.01 to 0.04) | 5.59 (2.57 to 10.14) | 2.76 (0.75 to 5.82) | 2.66 (0.74 to 5.58) | 2.54 (0.69 to 5.44) | -55 (-93 to 112) | -51 (-93 to 127) | -8 (-88 to 621) | -4 (-87 to 639) | -5 (-88 to 632) | 11.45 (5.26 to 20.78) | 2.06 (0.56 to 4.34) | 1.86 (0.52 to 3.89) | 1.66 (0.46 to 3.57) | 501.60 (230.43 to 910.05) | 248.02 (67.69 to 522.07) | 238.90 (66.67 to 500.30) | 227.51 (62.35 to 487.90) | -55 (-93 to 112) | -51 (-93 to 127) | -8 (-88 to 621) | -4 (-87 to 639) | -5 (-88 to 632) |
| **Central Europe** | | | | | | | | | | | | | | | | | | | | | | | | | | |
| **Albania** | 23.40 (6.83 to 57.99) | 3.15 (1.31 to 6.08) | 2.98 (1.25 to 5.73) | 2.74 (1.17 to 5.31) | 29.50 (8.61 to 73.10) | 10.86 (4.52 to 20.97) | 10.52 (4.42 to 20.23) | 9.87 (4.23 to 19.16) | -67 (-94 to 122) | -63 (-94 to 143) | -9 (-80 to 324) | -3 (-79 to 348) | -6 (-79 to 333) | 2,098.86 (613.09 to 5,201.35) | 282.52 (117.58 to 545.63) | 267.38 (112.44 to 514.27) | 245.58 (105.33 to 476.44) | 2,645.71 (772.83 to 6,556.56) | 973.72 (405.24 to 1,880.52) | 943.24 (396.66 to 1,814.18) | 885.51 (379.81 to 1,717.94) | -67 (-94 to 122) | -63 (-94 to 143) | -9 (-80 to 324) | -3 (-79 to 348) | -6 (-79 to 333) |
| **Bosnia and Herzegovina** | 6.13 (2.03 to 15.77) | 3.14 (0.87 to 7.23) | 2.86 (0.72 to 6.38) | 2.58 (0.65 to 5.81) | 9.13 (3.02 to 23.48) | 10.65 (2.94 to 24.49) | 9.97 (2.51 to 22.23) | 9.29 (2.33 to 20.88) | 2 (-90 to 592) | 17 (-87 to 711) | -13 (-90 to 609) | -6 (-90 to 655) | -7 (-90 to 731) | 550.13 (182.00 to 1,414.67) | 282.06 (77.94 to 648.38) | 256.77 (64.70 to 572.16) | 231.93 (58.13 to 521.41) | 819.09 (270.99 to 2,106.33) | 955.86 (264.13 to 2,197.24) | 895.12 (225.56 to 1,994.60) | 833.27 (208.85 to 1,873.27) | 2 (-90 to 592) | 17 (-87 to 711) | -13 (-90 to 609) | -6 (-90 to 655) | -7 (-90 to 731) |
| **Bulgaria** | 6.07 (4.50 to 8.13) | 2.41 (1.60 to 3.67) | 2.24 (1.40 to 3.50) | 2.17 (1.34 to 3.60) | 5.98 (4.43 to 8.01) | 3.94 (2.61 to 6.00) | 3.74 (2.33 to 5.84) | 3.71 (2.29 to 6.15) | -38 (-71 to 39) | -34 (-67 to 35) | -6 (-62 to 136) | -5 (-61 to 124) | -1 (-61 to 164) | 544.23 (403.21 to 729.76) | 216.47 (143.24 to 329.53) | 200.77 (125.32 to 314.16) | 194.87 (119.99 to 322.70) | 536.09 (397.18 to 718.84) | 353.74 (234.07 to 538.49) | 335.22 (209.24 to 524.55) | 333.35 (205.26 to 552.02) | -38 (-71 to 39) | -34 (-67 to 35) | -6 (-62 to 136) | -5 (-61 to 124) | -1 (-61 to 164) |
| **Croatia** | 13.92 (10.93 to 17.21) | 4.42 (3.11 to 6.55) | 4.14 (2.80 to 6.70) | 3.86 (2.56 to 6.28) | 24.82 (19.49 to 30.69) | 12.07 (8.47 to 17.86) | 11.50 (7.78 to 18.59) | 10.94 (7.25 to 17.82) | -56 (-76 to -9) | -51 (-72 to -8) | -9 (-59 to 110) | -5 (-56 to 120) | -5 (-61 to 129) | 1,248.98 (981.14 to 1,544.24) | 397.09 (278.75 to 587.75) | 371.83 (251.57 to 601.18) | 346.12 (229.46 to 563.71) | 2,226.75 (1,749.22 to 2,753.15) | 1,083.19 (760.38 to 1,603.26) | 1,032.33 (698.45 to 1,669.09) | 982.09 (651.08 to 1,599.50) | -56 (-76 to -9) | -51 (-72 to -8) | -9 (-59 to 110) | -5 (-56 to 120) | -5 (-61 to 129) |
| **Czech Republic** | 25.26 (20.17 to 31.47) | 10.06 (7.20 to 13.85) | 8.54 (5.84 to 12.30) | 7.90 (5.12 to 11.73) | 19.79 (15.80 to 24.66) | 8.90 (6.37 to 12.25) | 7.72 (5.28 to 11.12) | 7.36 (4.77 to 10.93) | -63 (-81 to -31) | -55 (-74 to -23) | -17 (-61 to 72) | -13 (-57 to 75) | -5 (-57 to 107) | 2,266.58 (1,809.87 to 2,824.22) | 903.14 (646.45 to 1,243.45) | 766.40 (524.20 to 1,103.63) | 709.27 (459.24 to 1,052.64) | 1,775.89 (1,418.05 to 2,212.81) | 798.42 (571.50 to 1,099.27) | 693.05 (474.03 to 998.01) | 660.87 (427.90 to 980.81) | -63 (-81 to -31) | -55 (-74 to -23) | -17 (-61 to 72) | -13 (-57 to 75) | -5 (-57 to 107) |
| **Hungary** | 31.85 (25.45 to 38.26) | 13.42 (10.00 to 18.65) | 12.55 (8.76 to 18.63) | 11.54 (7.84 to 17.36) | 25.78 (20.59 to 30.96) | 14.76 (11.00 to 20.52) | 13.89 (9.70 to 20.62) | 12.97 (8.82 to 19.52) | -50 (-72 to -5) | -43 (-64 to 0) | -12 (-57 to 77) | -6 (-53 to 87) | -7 (-57 to 101) | 2,857.24 (2,282.68 to 3,431.66) | 1,204.12 (897.44 to 1,673.83) | 1,126.19 (786.20 to 1,671.73) | 1,035.33 (703.55 to 1,557.81) | 2,312.31 (1,847.33 to 2,777.18) | 1,324.54 (987.19 to 1,841.22) | 1,246.60 (870.26 to 1,850.47) | 1,164.40 (791.26 to 1,752.01) | -50 (-72 to -5) | -43 (-64 to 0) | -12 (-57 to 77) | -6 (-53 to 87) | -7 (-57 to 101) |
| **Montenegro** | 1.91 (0.78 to 4.14) | 0.54 (0.12 to 1.23) | 0.50 (0.12 to 1.19) | 0.47 (0.10 to 1.12) | 19.38 (7.94 to 41.99) | 7.36 (1.67 to 16.95) | 6.97 (1.62 to 16.64) | 6.67 (1.49 to 15.84) | -66 (-96 to 99) | -62 (-96 to 113) | -9 (-91 to 848) | -5 (-90 to 896) | -4 (-91 to 878) | 171.50 (70.27 to 371.57) | 48.07 (10.91 to 110.74) | 44.80 (10.41 to 106.99) | 42.14 (9.41 to 100.11) | 1,738.21 (712.23 to 3,765.97) | 660.22 (149.89 to 1,521.18) | 625.17 (145.30 to 1,493.00) | 598.19 (133.53 to 1,421.21) | -66 (-96 to 99) | -62 (-96 to 113) | -9 (-91 to 848) | -5 (-90 to 896) | -4 (-91 to 878) |
| **North Macedonia** | 10.34 (2.55 to 25.49) | 1.86 (0.78 to 3.87) | 1.72 (0.67 to 3.52) | 1.44 (0.57 to 2.87) | 31.03 (7.66 to 76.46) | 9.14 (3.86 to 19.07) | 8.78 (3.42 to 17.96) | 7.58 (2.99 to 15.15) | -76 (-96 to 98) | -71 (-95 to 149) | -17 (-84 to 292) | -4 (-82 to 365) | -14 (-83 to 343) | 927.80 (229.08 to 2,285.96) | 166.70 (70.41 to 347.61) | 154.31 (60.10 to 315.55) | 128.97 (50.97 to 257.71) | 2,782.78 (687.07 to 6,856.37) | 820.54 (346.57 to 1,711.07) | 788.20 (306.97 to 1,611.84) | 680.15 (268.79 to 1,359.13) | -76 (-96 to 98) | -71 (-95 to 149) | -17 (-84 to 292) | -4 (-82 to 365) | -14 (-83 to 343) |
| **Republic of Poland** | 138.23 (113.34 to 167.27) | 16.61 (12.64 to 22.33) | 15.94 (11.37 to 22.70) | 14.53 (9.59 to 21.36) | 25.42 (20.84 to 30.76) | 4.40 (3.35 to 5.92) | 4.42 (3.16 to 6.30) | 4.21 (2.78 to 6.19) | -83 (-91 to -70) | -83 (-89 to -72) | -4 (-53 to 84) | 0 (-47 to 88) | -5 (-56 to 96) | 12,399.54 (10,166.23 to 15,004.00) | 1,490.28 (1,134.70 to 2,003.95) | 1,430.30 (1,020.29 to 2,037.31) | 1,304.15 (860.71 to 1,916.94) | 2,280.04 (1,869.37 to 2,758.95) | 395.27 (300.96 to 531.51) | 396.99 (283.19 to 565.47) | 377.72 (249.29 to 555.21) | -83 (-91 to -70) | -83 (-89 to -72) | -4 (-53 to 84) | 0 (-47 to 88) | -5 (-56 to 96) |
| **Romania** | 11.43 (8.75 to 15.20) | 3.07 (2.05 to 4.48) | 2.87 (1.84 to 4.32) | 2.77 (1.74 to 4.20) | 3.66 (2.80 to 4.86) | 1.64 (1.10 to 2.39) | 1.56 (1.00 to 2.35) | 1.54 (0.97 to 2.34) | -58 (-80 to -16) | -55 (-77 to -15) | -6 (-59 to 113) | -5 (-58 to 115) | -1 (-59 to 134) | 1,025.26 (784.64 to 1,363.48) | 275.47 (184.28 to 401.68) | 257.12 (164.87 to 387.47) | 248.41 (156.54 to 376.94) | 327.91 (250.95 to 436.09) | 147.10 (98.40 to 214.49) | 140.12 (89.85 to 211.15) | 138.43 (87.23 to 210.06) | -58 (-80 to -16) | -55 (-77 to -15) | -6 (-59 to 113) | -5 (-58 to 115) | -1 (-59 to 134) |
| **Republic of Serbia** | 28.23 (11.30 to 50.98) | 5.49 (1.56 to 11.29) | 5.13 (1.33 to 10.06) | 4.83 (1.31 to 9.72) | 20.77 (8.32 to 37.51) | 7.90 (2.25 to 16.27) | 7.50 (1.95 to 14.73) | 7.19 (1.95 to 14.47) | -65 (-95 to 74) | -62 (-94 to 96) | -9 (-88 to 542) | -5 (-88 to 554) | -4 (-87 to 643) | 2,531.71 (1,013.71 to 4,573.79) | 492.40 (140.29 to 1,013.61) | 460.10 (119.40 to 903.38) | 433.64 (117.80 to 872.74) | 1,862.74 (745.86 to 3,365.23) | 709.39 (202.11 to 1,460.29) | 673.40 (174.75 to 1,322.17) | 645.16 (175.26 to 1,298.46) | -65 (-95 to 74) | -62 (-94 to 96) | -9 (-88 to 542) | -5 (-88 to 554) | -4 (-87 to 643) |
| **Slovak Republic** | 11.23 (4.67 to 19.76) | 7.00 (2.92 to 13.05) | 6.50 (2.57 to 12.21) | 6.11 (2.38 to 11.46) | 14.25 (5.93 to 25.07) | 12.17 (5.07 to 22.69) | 11.43 (4.51 to 21.47) | 10.89 (4.25 to 20.45) | -24 (-83 to 245) | -15 (-80 to 283) | -10 (-81 to 303) | -6 (-80 to 323) | -5 (-80 to 353) | 1,007.52 (419.04 to 1,772.16) | 628.08 (261.84 to 1,171.03) | 583.44 (230.35 to 1,096.00) | 548.09 (213.66 to 1,028.83) | 1,278.46 (531.73 to 2,248.73) | 1,092.03 (455.26 to 2,036.03) | 1,025.49 (404.88 to 1,926.39) | 977.66 (381.12 to 1,835.16) | -24 (-83 to 245) | -15 (-80 to 283) | -10 (-81 to 303) | -6 (-80 to 323) | -5 (-80 to 353) |
| **Slovenia** | 3.97 (2.92 to 5.28) | 0.85 (0.58 to 1.20) | 0.78 (0.53 to 1.16) | 0.73 (0.48 to 1.11) | 17.61 (12.97 to 23.44) | 4.37 (2.96 to 6.15) | 4.10 (2.75 to 6.08) | 3.89 (2.56 to 5.92) | -78 (-89 to -54) | -75 (-87 to -53) | -11 (-58 to 100) | -6 (-55 to 105) | -5 (-58 to 115) | 355.92 (262.06 to 473.64) | 76.54 (51.90 to 107.61) | 70.30 (47.21 to 104.43) | 65.43 (43.04 to 99.68) | 1,580.28 (1,163.52 to 2,102.97) | 392.42 (266.08 to 551.72) | 367.69 (246.93 to 546.16) | 348.99 (229.59 to 531.66) | -78 (-89 to -54) | -75 (-87 to -53) | -11 (-58 to 100) | -6 (-55 to 105) | -5 (-58 to 115) |
| **Eastern Europe** | | | | | | | | | | | | | | | | | | | | | | | | | | |
| **Belarus** | 3.40 (2.14 to 5.06) | 1.52 (0.93 to 2.29) | 1.32 (0.80 to 2.08) | 1.19 (0.68 to 1.91) | 2.35 (1.48 to 3.51) | 1.65 (1.02 to 2.50) | 1.50 (0.91 to 2.37) | 1.42 (0.81 to 2.28) | -40 (-77 to 53) | -30 (-71 to 68) | -14 (-68 to 124) | -9 (-64 to 133) | -6 (-66 to 150) | 304.78 (191.89 to 454.08) | 135.96 (83.68 to 205.76) | 118.65 (71.69 to 186.98) | 107.24 (61.00 to 171.87) | 211.24 (133.00 to 314.72) | 147.99 (91.08 to 223.97) | 134.95 (81.54 to 212.67) | 127.43 (72.49 to 204.23) | -40 (-77 to 53) | -30 (-71 to 68) | -14 (-68 to 124) | -9 (-64 to 133) | -6 (-66 to 150) |
| **Estonia** | 5.14 (3.62 to 7.30) | 0.74 (0.51 to 1.04) | 0.71 (0.48 to 1.04) | 0.66 (0.44 to 0.96) | 23.15 (16.29 to 32.86) | 5.34 (3.66 to 7.46) | 5.25 (3.57 to 7.68) | 5.02 (3.37 to 7.33) | -78 (-90 to -55) | -77 (-89 to -54) | -6 (-55 to 100) | -2 (-52 to 110) | -4 (-56 to 105) | 461.19 (324.50 to 654.53) | 66.71 (45.72 to 93.12) | 63.54 (43.26 to 92.96) | 58.88 (39.52 to 85.95) | 2,077.17 (1,461.52 to 2,947.92) | 479.55 (328.70 to 669.44) | 471.08 (320.75 to 689.21) | 450.47 (302.30 to 657.51) | -78 (-90 to -55) | -77 (-89 to -54) | -6 (-55 to 100) | -2 (-52 to 110) | -4 (-56 to 105) |
| **Latvia** | 20.32 (15.08 to 26.69) | 3.98 (2.98 to 5.26) | 3.66 (2.67 to 4.97) | 3.31 (2.38 to 4.47) | 54.39 (40.36 to 71.43) | 21.14 (15.80 to 27.93) | 20.44 (14.95 to 27.74) | 19.41 (13.96 to 26.20) | -64 (-80 to -35) | -61 (-78 to -31) | -8 (-50 to 66) | -3 (-46 to 76) | -5 (-50 to 75) | 1,823.11 (1,352.70 to 2,394.43) | 357.33 (266.99 to 472.04) | 328.23 (240.05 to 445.65) | 297.14 (213.69 to 401.08) | 4,879.32 (3,620.33 to 6,408.38) | 1,897.24 (1,417.60 to 2,506.33) | 1,833.98 (1,341.29 to 2,490.09) | 1,741.66 (1,252.52 to 2,350.88) | -64 (-80 to -35) | -61 (-78 to -31) | -8 (-50 to 66) | -3 (-46 to 76) | -5 (-50 to 75) |
| **Lithuania** | 6.64 (4.73 to 9.20) | 2.58 (1.91 to 3.42) | 2.20 (1.60 to 2.91) | 2.04 (1.48 to 2.76) | 11.85 (8.45 to 16.42) | 9.59 (7.13 to 12.74) | 8.66 (6.32 to 11.46) | 8.48 (6.15 to 11.49) | -28 (-63 to 36) | -19 (-57 to 51) | -12 (-52 to 61) | -10 (-50 to 61) | -2 (-46 to 82) | 595.80 (424.60 to 825.31) | 231.17 (171.77 to 307.11) | 197.14 (143.77 to 261.01) | 182.87 (132.65 to 247.65) | 1,063.53 (757.93 to 1,473.23) | 860.65 (639.49 to 1,143.37) | 777.17 (566.78 to 1,028.95) | 761.20 (552.15 to 1,030.85) | -28 (-63 to 36) | -19 (-57 to 51) | -12 (-52 to 61) | -10 (-50 to 61) | -2 (-46 to 82) |
| **Republic of Moldova** | 3.07 (2.04 to 4.38) | 4.24 (2.90 to 5.95) | 3.94 (2.62 to 5.72) | 3.65 (2.37 to 5.45) | 3.84 (2.55 to 5.47) | 13.67 (9.36 to 19.20) | 13.25 (8.82 to 19.25) | 12.81 (8.32 to 19.12) | 233 (52 to 649) | 256 (71 to 652) | -6 (-57 to 104) | -3 (-54 to 106) | -3 (-57 to 117) | 275.58 (183.01 to 392.52) | 380.27 (260.46 to 534.30) | 353.40 (235.36 to 513.40) | 327.31 (212.54 to 488.69) | 344.70 (228.91 to 490.97) | 1,226.45 (840.05 to 1,723.27) | 1,189.02 (791.85 to 1,727.35) | 1,149.44 (746.41 to 1,716.16) | 233 (52 to 649) | 256 (71 to 652) | -6 (-57 to 104) | -3 (-54 to 106) | -3 (-57 to 117) |
| **Russian Federation** | 574.28 (504.16 to 656.94) | 191.40 (169.37 to 222.34) | 200.16 (174.81 to 237.05) | 196.50 (165.24 to 236.86) | 28.15 (24.71 to 32.20) | 12.75 (11.28 to 14.81) | 14.00 (12.22 to 16.58) | 14.33 (12.05 to 17.27) | -49 (-63 to -30) | -55 (-65 to -40) | 12 (-19 to 53) | 10 (-17 to 47) | 2 (-27 to 41) | 51,514.20 (45,219.55 to 58,931.59) | 17,174.14 (15,196.94 to 19,951.45) | 17,962.32 (15,687.34 to 21,274.51) | 17,633.04 (14,828.04 to 21,255.67) | 2,525.10 (2,216.55 to 2,888.68) | 1,144.24 (1,012.51 to 1,329.28) | 1,256.14 (1,097.04 to 1,487.76) | 1,285.84 (1,081.29 to 1,550.01) | -49 (-63 to -30) | -55 (-65 to -40) | 12 (-19 to 53) | 10 (-17 to 47) | 2 (-27 to 41) |
| **Ukraine** | 235.51 (156.11 to 347.81) | 62.38 (43.74 to 98.68) | 60.55 (41.46 to 100.51) | 54.95 (36.10 to 95.54) | 34.77 (23.05 to 51.35) | 19.91 (13.96 to 31.49) | 20.58 (14.09 to 34.16) | 19.72 (12.96 to 34.29) | -43 (-75 to 49) | -43 (-73 to 37) | -1 (-59 to 146) | 3 (-55 to 145) | -4 (-62 to 143) | 21,128.28 (14,000.54 to 31,202.25) | 5,598.37 (3,924.67 to 8,856.41) | 5,434.42 (3,719.86 to 9,020.81) | 4,931.60 (3,240.48 to 8,574.13) | 3,119.22 (2,066.94 to 4,606.47) | 1,786.76 (1,252.59 to 2,826.59) | 1,847.18 (1,264.40 to 3,066.21) | 1,769.94 (1,163.00 to 3,077.24) | -43 (-75 to 49) | -43 (-73 to 37) | -1 (-59 to 146) | 3 (-55 to 145) | -4 (-62 to 143) |
| **Western Europe** | | | | | | | | | | | | | | | | | | | | | | | | | | |
| **Andorra** | 0.16 (0.07 to 0.27) | 0.03 (0.01 to 0.06) | 0.03 (0.01 to 0.06) | 0.02 (0.00 to 0.03) | 32.29 (13.75 to 53.84) | 7.74 (3.04 to 13.46) | 7.23 (2.77 to 12.80) | 3.86 (1.06 to 7.54) | -88 (-98 to -45) | -76 (-94 to -2) | -50 (-92 to 148) | -7 (-79 to 320) | -47 (-92 to 172) | 14.75 (6.28 to 24.59) | 3.11 (1.22 to 5.40) | 2.94 (1.13 to 5.20) | 1.59 (0.44 to 3.11) | 2,897.08 (1,233.43 to 4,832.14) | 695.09 (273.31 to 1,207.86) | 648.71 (248.73 to 1,149.22) | 346.61 (95.38 to 676.94) | -88 (-98 to -45) | -76 (-94 to -2) | -50 (-92 to 148) | -7 (-79 to 320) | -47 (-92 to 172) |
| **Australia** | 116.84 (105.43 to 128.26) | 9.86 (8.06 to 12.24) | 8.57 (6.74 to 10.68) | 8.09 (6.21 to 10.33) | 129.24 (116.62 to 141.87) | 11.65 (9.52 to 14.46) | 10.18 (8.01 to 12.69) | 9.58 (7.35 to 12.22) | -93 (-95 to -90) | -91 (-93 to -88) | -18 (-49 to 28) | -13 (-45 to 33) | -6 (-42 to 53) | 10,483.46 (9,459.94 to 11,508.07) | 884.61 (723.09 to 1,098.68) | 769.21 (605.06 to 958.22) | 726.38 (557.46 to 927.18) | 11,596.08 (10,463.93 to 12,729.44) | 1,045.09 (854.27 to 1,298.00) | 914.02 (718.96 to 1,138.61) | 859.42 (659.57 to 1,097.00) | -93 (-95 to -90) | -91 (-93 to -88) | -18 (-49 to 28) | -13 (-45 to 33) | -6 (-42 to 53) |
| **Belgium** | 180.98 (163.49 to 199.83) | 15.94 (11.72 to 20.58) | 13.97 (10.16 to 18.61) | 13.58 (9.53 to 19.05) | 147.40 (133.15 to 162.75) | 13.62 (10.02 to 17.59) | 12.14 (8.83 to 16.18) | 11.92 (8.37 to 16.73) | -92 (-95 to -87) | -91 (-94 to -87) | -12 (-52 to 67) | -11 (-50 to 61) | -2 (-48 to 90) | 16,237.95 (14,668.41 to 17,927.89) | 1,430.26 (1,051.86 to 1,846.89) | 1,253.63 (911.54 to 1,670.67) | 1,218.75 (855.56 to 1,709.91) | 13,224.63 (11,946.35 to 14,600.96) | 1,222.43 (899.01 to 1,578.52) | 1,089.39 (792.13 to 1,451.80) | 1,070.22 (751.29 to 1,501.52) | -92 (-95 to -87) | -91 (-94 to -87) | -12 (-52 to 67) | -11 (-50 to 61) | -2 (-48 to 90) |
| **Cyprus** | 7.06 (2.19 to 13.53) | 1.76 (0.77 to 3.38) | 1.68 (0.72 to 3.28) | 1.64 (0.71 to 3.14) | 52.35 (16.21 to 100.26) | 11.82 (5.13 to 22.69) | 11.21 (4.82 to 21.86) | 10.92 (4.72 to 20.90) | -79 (-95 to 29) | -77 (-95 to 40) | -8 (-79 to 307) | -5 (-79 to 326) | -3 (-78 to 334) | 633.74 (196.21 to 1,213.96) | 158.25 (68.71 to 303.74) | 150.80 (64.84 to 294.17) | 147.14 (63.59 to 281.52) | 4,697.13 (1,454.22 to 8,997.56) | 1,061.12 (460.76 to 2,036.76) | 1,005.82 (432.46 to 1,962.01) | 980.53 (423.75 to 1,876.03) | -79 (-95 to 29) | -77 (-95 to 40) | -8 (-79 to 307) | -5 (-79 to 326) | -3 (-78 to 334) |
| **Denmark** | 14.22 (11.43 to 17.49) | 1.73 (1.29 to 2.33) | 1.69 (1.23 to 2.30) | 1.70 (1.19 to 2.36) | 22.88 (18.39 to 28.14) | 2.84 (2.12 to 3.83) | 2.75 (1.99 to 3.73) | 2.70 (1.90 to 3.74) | -88 (-93 to -80) | -88 (-92 to -79) | -5 (-50 to 76) | -3 (-48 to 76) | -2 (-49 to 88) | 1,276.10 (1,025.46 to 1,569.66) | 155.20 (116.13 to 209.30) | 152.04 (110.02 to 206.24) | 152.34 (107.08 to 211.49) | 2,053.11 (1,649.86 to 2,525.41) | 254.71 (190.60 to 343.51) | 247.04 (178.76 to 335.11) | 242.07 (170.16 to 336.06) | -88 (-93 to -80) | -88 (-92 to -79) | -5 (-50 to 76) | -3 (-48 to 76) | -2 (-49 to 88) |
| **Finland** | 19.20 (15.97 to 22.56) | 2.72 (2.12 to 3.43) | 2.51 (1.90 to 3.23) | 2.50 (1.90 to 3.31) | 29.74 (24.75 to 34.95) | 5.79 (4.52 to 7.33) | 5.33 (4.02 to 6.84) | 5.20 (3.94 to 6.88) | -82 (-89 to -72) | -81 (-87 to -70) | -10 (-46 to 52) | -8 (-45 to 51) | -2 (-42 to 71) | 1,722.43 (1,433.12 to 2,024.12) | 243.77 (190.16 to 308.23) | 225.39 (170.28 to 289.55) | 224.75 (170.31 to 297.17) | 2,668.28 (2,220.10 to 3,135.65) | 520.03 (405.67 to 657.53) | 478.02 (361.14 to 614.11) | 467.16 (354.00 to 617.69) | -82 (-89 to -72) | -81 (-87 to -70) | -10 (-46 to 52) | -8 (-45 to 51) | -2 (-42 to 71) |
| **French Republic** | 1,171.70 (1,085.66 to 1,260.59) | 133.64 (110.86 to 156.90) | 122.40 (98.23 to 150.39) | 117.34 (90.76 to 153.52) | 154.22 (142.89 to 165.92) | 19.00 (15.76 to 22.31) | 17.65 (14.17 to 21.69) | 17.09 (13.22 to 22.36) | -89 (-92 to -84) | -88 (-90 to -84) | -10 (-41 to 42) | -7 (-37 to 38) | -3 (-39 to 58) | 105,125.97 (97,404.45 to 113,105.31) | 11,993.17 (9,948.15 to 14,080.88) | 10,985.70 (8,815.58 to 13,498.54) | 10,531.11 (8,146.21 to 13,777.70) | 13,836.42 (12,820.13 to 14,886.64) | 1,705.44 (1,414.64 to 2,002.32) | 1,584.28 (1,271.32 to 1,946.67) | 1,533.53 (1,186.24 to 2,006.30) | -89 (-92 to -84) | -88 (-90 to -84) | -10 (-41 to 42) | -7 (-37 to 38) | -3 (-39 to 58) |
| **Federal Republic of Germany** | 1,075.32 (986.50 to 1,167.14) | 98.75 (80.96 to 120.32) | 89.97 (70.17 to 113.41) | 87.82 (65.53 to 116.09) | 121.76 (111.70 to 132.15) | 12.55 (10.29 to 15.29) | 11.50 (8.97 to 14.49) | 11.24 (8.39 to 14.86) | -91 (-94 to -87) | -90 (-92 to -86) | -10 (-45 to 44) | -8 (-41 to 41) | -2 (-42 to 66) | 96,474.81 (88,501.43 to 104,715.87) | 8,861.45 (7,265.19 to 10,797.62) | 8,075.22 (6,298.09 to 10,178.78) | 7,881.72 (5,880.85 to 10,419.74) | 10,923.65 (10,020.84 to 11,856.77) | 1,126.32 (923.43 to 1,372.42) | 1,032.09 (804.96 to 1,300.95) | 1,008.87 (752.76 to 1,333.75) | -91 (-94 to -87) | -90 (-92 to -86) | -10 (-45 to 44) | -8 (-41 to 41) | -2 (-42 to 66) |
| **Hellenic Republic** | 11.28 (9.18 to 13.57) | 2.31 (1.73 to 3.15) | 2.03 (1.48 to 2.78) | 1.93 (1.36 to 2.73) | 10.91 (8.88 to 13.13) | 2.71 (2.02 to 3.69) | 2.40 (1.76 to 3.29) | 2.33 (1.64 to 3.30) | -79 (-88 to -63) | -75 (-85 to -58) | -14 (-56 to 63) | -11 (-52 to 63) | -3 (-50 to 87) | 1,012.29 (823.69 to 1,217.97) | 207.28 (154.83 to 283.02) | 181.81 (132.97 to 249.07) | 173.12 (121.84 to 245.01) | 979.08 (796.66 to 1,178.00) | 242.80 (181.36 to 331.52) | 215.78 (157.82 to 295.62) | 209.01 (147.10 to 295.80) | -79 (-88 to -63) | -75 (-85 to -58) | -14 (-56 to 63) | -11 (-52 to 63) | -3 (-50 to 87) |
| **Iceland** | 3.14 (2.67 to 3.62) | 0.36 (0.28 to 0.45) | 0.34 (0.27 to 0.44) | 0.34 (0.26 to 0.44) | 68.74 (58.54 to 79.29) | 8.19 (6.47 to 10.36) | 7.63 (5.90 to 9.70) | 7.41 (5.64 to 9.61) | -89 (-93 to -84) | -88 (-92 to -82) | -10 (-46 to 48) | -7 (-43 to 50) | -3 (-42 to 63) | 281.81 (239.99 to 325.03) | 32.18 (25.45 to 40.73) | 30.82 (23.84 to 39.16) | 30.47 (23.20 to 39.52) | 6,167.75 (5,252.39 to 7,113.64) | 734.71 (581.07 to 929.69) | 685.16 (529.95 to 870.50) | 664.68 (506.03 to 862.10) | -89 (-93 to -84) | -88 (-92 to -82) | -10 (-46 to 48) | -7 (-43 to 50) | -3 (-42 to 63) |
| **Ireland** | 71.13 (64.51 to 78.12) | 10.91 (8.31 to 14.15) | 9.57 (7.17 to 12.81) | 9.37 (6.56 to 13.08) | 131.59 (119.34 to 144.52) | 18.43 (14.04 to 23.92) | 16.63 (12.45 to 22.25) | 16.54 (11.57 to 23.07) | -87 (-92 to -81) | -86 (-90 to -80) | -10 (-52 to 64) | -10 (-48 to 59) | -1 (-48 to 85) | 6,382.31 (5,787.89 to 7,009.47) | 978.88 (745.49 to 1,270.09) | 859.33 (643.15 to 1,149.86) | 841.33 (588.77 to 1,173.67) | 11,806.89 (10,707.25 to 12,967.11) | 1,654.13 (1,259.75 to 2,146.22) | 1,492.43 (1,116.98 to 1,996.99) | 1,484.14 (1,038.61 to 2,070.39) | -87 (-92 to -81) | -86 (-90 to -80) | -10 (-52 to 64) | -10 (-48 to 59) | -1 (-48 to 85) |
| **Israel** | 42.55 (28.28 to 55.57) | 12.48 (9.78 to 15.44) | 10.03 (7.73 to 12.75) | 8.39 (6.02 to 11.30) | 41.44 (27.54 to 54.11) | 6.88 (5.39 to 8.51) | 5.54 (4.27 to 7.04) | 4.59 (3.29 to 6.18) | -89 (-94 to -78) | -83 (-90 to -69) | -33 (-61 to 15) | -19 (-50 to 31) | -17 (-53 to 45) | 3,818.38 (2,537.67 to 4,986.23) | 1,120.28 (878.04 to 1,385.28) | 900.55 (693.92 to 1,144.01) | 753.34 (540.55 to 1,013.98) | 3,718.18 (2,471.08 to 4,855.39) | 617.32 (483.83 to 763.34) | 497.20 (383.12 to 631.63) | 411.93 (295.58 to 554.46) | -89 (-94 to -78) | -83 (-90 to -69) | -33 (-61 to 15) | -19 (-50 to 31) | -17 (-53 to 45) |
| **Italy** | 74.63 (65.97 to 83.47) | 12.31 (10.21 to 15.05) | 12.13 (9.46 to 15.19) | 11.51 (8.52 to 15.17) | 13.47 (11.91 to 15.07) | 2.88 (2.39 to 3.53) | 2.94 (2.29 to 3.68) | 2.85 (2.11 to 3.75) | -79 (-86 to -68) | -79 (-84 to -70) | -1 (-40 to 57) | 2 (-35 to 54) | -3 (-43 to 64) | 6,696.69 (5,920.02 to 7,490.54) | 1,104.54 (916.78 to 1,350.71) | 1,088.97 (849.26 to 1,363.40) | 1,033.46 (764.86 to 1,361.58) | 1,208.76 (1,068.57 to 1,352.05) | 258.83 (214.84 to 316.52) | 263.74 (205.68 to 330.20) | 255.78 (189.30 to 336.99) | -79 (-86 to -68) | -79 (-84 to -70) | -1 (-40 to 57) | 2 (-35 to 54) | -3 (-43 to 64) |
| **Grand Duchy of Luxembourg** | 8.05 (7.26 to 8.93) | 1.12 (0.85 to 1.42) | 1.08 (0.81 to 1.41) | 1.05 (0.79 to 1.41) | 164.35 (148.08 to 182.28) | 17.66 (13.34 to 22.37) | 16.61 (12.50 to 21.82) | 15.85 (11.83 to 21.21) | -90 (-94 to -86) | -89 (-93 to -85) | -10 (-47 to 59) | -6 (-44 to 64) | -5 (-46 to 70) | 722.68 (651.11 to 801.54) | 100.43 (75.89 to 127.25) | 96.62 (72.73 to 126.91) | 94.54 (70.56 to 126.54) | 14,746.44 (13,286.06 to 16,355.72) | 1,584.80 (1,197.51 to 2,007.98) | 1,490.70 (1,122.12 to 1,957.99) | 1,422.38 (1,061.68 to 1,903.85) | -90 (-94 to -86) | -89 (-93 to -85) | -10 (-47 to 59) | -6 (-44 to 64) | -5 (-46 to 70) |
| **Republic of Malta** | 0.45 (0.35 to 0.58) | 0.08 (0.06 to 0.10) | 0.07 (0.05 to 0.09) | 0.07 (0.05 to 0.09) | 8.17 (6.41 to 10.46) | 1.75 (1.26 to 2.27) | 1.52 (1.10 to 2.02) | 1.51 (1.06 to 2.05) | -82 (-90 to -68) | -79 (-88 to -65) | -14 (-53 to 63) | -13 (-52 to 61) | -1 (-47 to 86) | 40.47 (31.77 to 51.81) | 6.93 (4.99 to 8.99) | 5.99 (4.33 to 7.98) | 5.88 (4.14 to 7.98) | 733.01 (575.36 to 938.45) | 156.99 (113.04 to 203.62) | 136.40 (98.60 to 181.61) | 135.60 (95.51 to 183.78) | -82 (-90 to -68) | -79 (-88 to -65) | -14 (-53 to 63) | -13 (-52 to 61) | -1 (-47 to 86) |
| **Principality of Monaco** | 0.12 (0.04 to 0.21) | 0.04 (0.01 to 0.07) | 0.09 (0.02 to 0.15) | 0.09 (0.02 to 0.15) | 43.89 (15.83 to 79.98) | 12.93 (3.55 to 22.42) | 27.40 (7.41 to 45.94) | 27.53 (7.59 to 46.89) | -37 (-91 to 196) | -71 (-96 to 42) | 113 (-66 to 1,220) | 112 (-67 to 1,192) | 0 (-83 to 533) | 10.48 (3.78 to 19.10) | 3.70 (1.02 to 6.41) | 7.80 (2.11 to 13.07) | 7.81 (2.15 to 13.30) | 3,937.67 (1,420.37 to 7,176.08) | 1,160.20 (318.73 to 2,011.84) | 2,456.09 (664.34 to 4,116.70) | 2,467.93 (680.78 to 4,203.05) | -37 (-91 to 196) | -71 (-96 to 42) | 113 (-66 to 1,220) | 112 (-67 to 1,192) | 0 (-83 to 533) |
| **Netherlands** | 120.10 (107.32 to 133.86) | 14.16 (11.21 to 17.64) | 13.40 (10.42 to 16.91) | 13.17 (9.92 to 17.01) | 62.01 (55.41 to 69.11) | 8.36 (6.61 to 10.41) | 7.79 (6.06 to 9.83) | 7.47 (5.63 to 9.66) | -88 (-92 to -83) | -87 (-90 to -81) | -11 (-46 to 46) | -7 (-42 to 49) | -4 (-43 to 59) | 10,776.19 (9,629.50 to 12,010.57) | 1,270.90 (1,005.99 to 1,583.42) | 1,202.51 (935.16 to 1,517.79) | 1,181.66 (890.58 to 1,526.45) | 5,563.66 (4,971.63 to 6,200.96) | 749.89 (593.58 to 934.29) | 699.01 (543.60 to 882.28) | 670.87 (505.61 to 866.62) | -88 (-92 to -83) | -87 (-90 to -81) | -11 (-46 to 46) | -7 (-42 to 49) | -4 (-43 to 59) |
| **Norway** | 71.07 (65.19 to 77.10) | 6.02 (5.18 to 7.02) | 5.12 (4.37 to 5.92) | 5.38 (4.51 to 6.41) | 121.63 (111.57 to 131.97) | 11.02 (9.50 to 12.87) | 9.42 (8.05 to 10.91) | 9.75 (8.18 to 11.62) | -92 (-94 to -90) | -91 (-93 to -88) | -12 (-36 to 22) | -14 (-37 to 15) | 3 (-25 to 44) | 6,376.78 (5,848.91 to 6,918.83) | 539.88 (465.34 to 630.49) | 459.33 (392.28 to 531.71) | 482.57 (404.98 to 575.40) | 10,914.04 (10,010.58 to 11,841.77) | 989.33 (852.72 to 1,155.36) | 845.88 (722.42 to 979.17) | 875.04 (734.35 to 1,043.37) | -92 (-94 to -90) | -91 (-93 to -88) | -12 (-36 to 22) | -14 (-37 to 15) | 3 (-25 to 44) |
| **Portuguese Republic** | 12.42 (9.89 to 15.31) | 1.23 (0.92 to 1.63) | 1.01 (0.75 to 1.37) | 0.93 (0.66 to 1.27) | 10.87 (8.65 to 13.39) | 1.41 (1.06 to 1.88) | 1.19 (0.88 to 1.61) | 1.13 (0.81 to 1.54) | -90 (-94 to -82) | -87 (-92 to -78) | -20 (-57 to 46) | -16 (-53 to 53) | -5 (-50 to 75) | 1,114.70 (887.09 to 1,373.67) | 109.98 (82.50 to 146.58) | 90.52 (67.02 to 122.78) | 83.39 (59.61 to 113.90) | 975.29 (776.14 to 1,201.87) | 126.54 (94.92 to 168.66) | 106.77 (79.04 to 144.82) | 101.52 (72.58 to 138.67) | -90 (-94 to -82) | -87 (-92 to -78) | -20 (-57 to 46) | -16 (-53 to 53) | -5 (-50 to 75) |
| **San Marino** | 0.10 (0.03 to 0.17) | 0.03 (0.01 to 0.05) | 0.03 (0.01 to 0.05) | 0.02 (0.01 to 0.04) | 40.01 (14.11 to 70.00) | 13.06 (5.10 to 22.44) | 12.10 (4.82 to 21.07) | 9.49 (3.65 to 16.86) | -76 (-95 to 19) | -67 (-93 to 59) | -27 (-84 to 231) | -7 (-79 to 313) | -22 (-83 to 250) | 8.60 (3.03 to 15.04) | 2.79 (1.09 to 4.80) | 2.53 (1.01 to 4.41) | 1.96 (0.75 to 3.48) | 3,589.95 (1,266.42 to 6,278.79) | 1,171.48 (457.36 to 2,013.29) | 1,085.67 (432.69 to 1,890.71) | 851.35 (328.01 to 1,513.55) | -76 (-95 to 19) | -67 (-93 to 59) | -27 (-84 to 231) | -7 (-79 to 313) | -22 (-83 to 250) |
| **Kingdom of Spain** | 130.61 (112.06 to 151.43) | 29.15 (22.89 to 37.57) | 25.46 (19.14 to 33.96) | 24.07 (17.42 to 33.21) | 32.91 (28.24 to 38.16) | 8.07 (6.34 to 10.40) | 7.32 (5.50 to 9.77) | 7.12 (5.15 to 9.82) | -78 (-86 to -65) | -75 (-83 to -63) | -12 (-50 to 55) | -9 (-47 to 54) | -3 (-47 to 78) | 11,720.36 (10,054.68 to 13,589.00) | 2,616.46 (2,054.38 to 3,371.62) | 2,285.02 (1,718.02 to 3,048.62) | 2,160.05 (1,563.25 to 2,980.63) | 2,953.28 (2,533.56 to 3,424.13) | 724.65 (568.98 to 933.80) | 657.05 (494.01 to 876.62) | 639.00 (462.45 to 881.75) | -78 (-86 to -65) | -75 (-83 to -63) | -12 (-50 to 55) | -9 (-47 to 54) | -3 (-47 to 78) |
| **Kingdom of Sweden** | 107.17 (98.51 to 116.37) | 11.71 (9.92 to 13.80) | 11.61 (9.49 to 14.33) | 10.43 (8.52 to 13.01) | 88.42 (81.27 to 96.00) | 10.23 (8.66 to 12.06) | 10.24 (8.36 to 12.63) | 9.22 (7.54 to 11.50) | -90 (-92 to -86) | -88 (-91 to -85) | -10 (-37 to 33) | 0 (-31 to 46) | -10 (-40 to 38) | 9,616.15 (8,838.58 to 10,441.56) | 1,050.99 (889.82 to 1,238.60) | 1,042.27 (851.43 to 1,286.08) | 936.24 (765.07 to 1,167.71) | 7,933.60 (7,292.08 to 8,614.59) | 918.17 (777.37 to 1,082.07) | 919.01 (750.74 to 1,133.99) | 827.92 (676.56 to 1,032.61) | -90 (-92 to -86) | -88 (-91 to -85) | -10 (-37 to 33) | 0 (-31 to 46) | -10 (-40 to 38) |
| **Swiss Confederation** | 20.80 (16.90 to 25.31) | 2.81 (2.03 to 3.84) | 2.73 (1.91 to 3.98) | 2.67 (1.83 to 3.92) | 25.32 (20.58 to 30.82) | 3.28 (2.36 to 4.47) | 3.18 (2.22 to 4.63) | 3.06 (2.10 to 4.50) | -88 (-93 to -78) | -87 (-92 to -78) | -7 (-53 to 91) | -3 (-50 to 96) | -4 (-55 to 103) | 1,866.06 (1,516.05 to 2,271.40) | 252.56 (181.86 to 344.26) | 245.30 (171.30 to 357.56) | 239.45 (164.36 to 351.77) | 2,272.17 (1,845.99 to 2,765.72) | 294.00 (211.70 to 400.75) | 285.09 (199.09 to 415.56) | 274.83 (188.64 to 403.74) | -88 (-93 to -78) | -87 (-92 to -78) | -7 (-53 to 91) | -3 (-50 to 96) | -4 (-55 to 103) |
| **United Kingdom** | 1,027.41 (985.55 to 1,076.81) | 103.79 (95.52 to 112.50) | 82.43 (74.24 to 91.45) | 87.33 (71.75 to 102.00) | 131.12 (125.78 to 137.43) | 14.46 (13.30 to 15.67) | 11.77 (10.60 to 13.06) | 12.62 (10.37 to 14.74) | -90 (-92 to -88) | -89 (-90 to -88) | -13 (-34 to 11) | -19 (-32 to -2) | 7 (-21 to 39) | 92,184.85 (88,429.32 to 96,620.04) | 9,314.49 (8,572.23 to 10,096.41) | 7,398.29 (6,663.81 to 8,208.36) | 7,838.68 (6,439.69 to 9,155.36) | 11,765.20 (11,285.89 to 12,331.24) | 1,297.23 (1,193.86 to 1,406.13) | 1,056.62 (951.72 to 1,172.32) | 1,132.93 (930.73 to 1,323.22) | -90 (-92 to -88) | -89 (-90 to -88) | -13 (-34 to 11) | -19 (-32 to -2) | 7 (-21 to 39) |
| **Andean Latin America** | | | | | | | | | | | | | | | | | | | | | | | | | | |
| **Plurinational State of Bolivia** | 86.61 (33.65 to 177.27) | 26.68 (13.37 to 48.91) | 25.41 (12.67 to 45.20) | 24.16 (12.32 to 43.21) | 40.18 (15.61 to 82.23) | 10.98 (5.50 to 20.13) | 10.54 (5.26 to 18.76) | 10.09 (5.15 to 18.06) | -75 (-94 to 16) | -73 (-93 to 29) | -8 (-74 to 228) | -4 (-74 to 241) | -4 (-73 to 244) | 7,774.19 (3,020.12 to 15,913.38) | 2,394.57 (1,200.35 to 4,390.09) | 2,281.18 (1,136.95 to 4,057.87) | 2,168.37 (1,106.07 to 3,879.04) | 3,606.01 (1,400.87 to 7,381.33) | 985.77 (494.14 to 1,807.26) | 946.54 (471.76 to 1,683.75) | 906.10 (462.20 to 1,620.95) | -75 (-94 to 16) | -73 (-93 to 29) | -8 (-74 to 228) | -4 (-74 to 241) | -4 (-73 to 244) |
| **Ecuador** | 42.75 (29.28 to 65.84) | 11.86 (7.86 to 17.04) | 10.37 (7.03 to 15.23) | 9.79 (6.23 to 15.12) | 14.91 (10.21 to 22.97) | 3.58 (2.38 to 5.15) | 3.18 (2.16 to 4.67) | 3.08 (1.96 to 4.76) | -79 (-91 to -53) | -76 (-90 to -50) | -14 (-62 to 100) | -11 (-58 to 97) | -3 (-58 to 121) | 3,836.85 (2,627.58 to 5,909.23) | 1,064.58 (705.75 to 1,529.68) | 930.94 (630.67 to 1,366.78) | 878.16 (559.18 to 1,357.34) | 1,338.46 (916.62 to 2,061.40) | 321.66 (213.24 to 462.19) | 285.56 (193.46 to 419.25) | 276.35 (175.97 to 427.15) | -79 (-91 to -53) | -76 (-90 to -50) | -14 (-62 to 100) | -11 (-58 to 97) | -3 (-58 to 121) |
| **Peru** | 153.94 (66.52 to 304.82) | 41.18 (20.23 to 75.46) | 35.56 (17.16 to 65.90) | 32.82 (15.45 to 60.96) | 24.97 (10.79 to 49.44) | 6.23 (3.06 to 11.41) | 5.33 (2.57 to 9.89) | 4.93 (2.32 to 9.16) | -80 (-95 to -15) | -75 (-94 to 6) | -21 (-80 to 200) | -14 (-77 to 223) | -8 (-77 to 256) | 13,816.23 (5,970.29 to 27,351.54) | 3,695.96 (1,815.77 to 6,773.46) | 3,191.38 (1,540.46 to 5,910.46) | 2,945.32 (1,386.94 to 5,469.63) | 2,241.03 (968.40 to 4,436.49) | 558.83 (274.55 to 1,024.15) | 478.75 (231.09 to 886.65) | 442.76 (208.49 to 822.23) | -80 (-95 to -15) | -75 (-94 to 6) | -21 (-80 to 200) | -14 (-77 to 223) | -8 (-77 to 256) |
| **Central Latin America** | | | | | | | | | | | | | | | | | | | | | | | | | | |
| **Colombia** | 84.83 (67.71 to 103.86) | 55.14 (39.34 to 76.40) | 50.54 (35.15 to 73.06) | 47.87 (31.52 to 70.43) | 9.55 (7.62 to 11.69) | 7.97 (5.69 to 11.04) | 7.35 (5.11 to 10.63) | 7.13 (4.70 to 10.49) | -25 (-60 to 38) | -17 (-51 to 45) | -10 (-57 to 85) | -8 (-54 to 87) | -3 (-56 to 105) | 7,612.16 (6,075.49 to 9,319.82) | 4,948.34 (3,531.27 to 6,856.21) | 4,535.35 (3,154.64 to 6,556.34) | 4,296.09 (2,828.94 to 6,321.31) | 856.72 (683.78 to 1,048.91) | 715.14 (510.34 to 990.87) | 659.95 (459.04 to 954.03) | 640.14 (421.52 to 941.90) | -25 (-60 to 38) | -17 (-51 to 45) | -10 (-57 to 85) | -8 (-54 to 87) | -3 (-56 to 105) |
| **Costa Rica** | 6.26 (4.76 to 8.10) | 2.77 (1.55 to 5.68) | 2.30 (1.29 to 4.70) | 2.12 (1.14 to 4.49) | 7.78 (5.92 to 10.07) | 4.45 (2.48 to 9.13) | 3.96 (2.22 to 8.08) | 3.86 (2.07 to 8.19) | -50 (-79 to 38) | -43 (-75 to 54) | -13 (-77 to 230) | -11 (-76 to 225) | -2 (-74 to 269) | 561.95 (427.55 to 726.72) | 248.58 (138.71 to 509.67) | 206.55 (115.96 to 421.22) | 190.11 (102.04 to 402.62) | 698.53 (531.46 to 903.34) | 399.02 (222.66 to 818.13) | 355.23 (199.43 to 724.40) | 346.57 (186.02 to 733.97) | -50 (-79 to 38) | -43 (-75 to 54) | -13 (-77 to 230) | -11 (-76 to 225) | -2 (-74 to 269) |
| **El Salvador** | 20.51 (8.41 to 37.56) | 7.27 (2.81 to 13.93) | 6.84 (2.56 to 13.54) | 6.48 (2.48 to 13.13) | 12.32 (5.05 to 22.56) | 5.99 (2.32 to 11.48) | 5.76 (2.16 to 11.41) | 5.59 (2.14 to 11.32) | -55 (-91 to 124) | -51 (-90 to 127) | -7 (-81 to 389) | -4 (-81 to 392) | -3 (-81 to 425) | 1,840.76 (755.18 to 3,370.28) | 652.75 (252.39 to 1,249.92) | 613.80 (229.75 to 1,215.26) | 581.85 (222.64 to 1,178.32) | 1,105.74 (453.64 to 2,024.52) | 537.95 (208.01 to 1,030.09) | 517.35 (193.65 to 1,024.29) | 501.83 (192.03 to 1,016.28) | -55 (-91 to 124) | -51 (-90 to 127) | -7 (-81 to 389) | -4 (-81 to 392) | -3 (-81 to 425) |
| **Guatemala** | 93.88 (71.41 to 129.05) | 83.68 (66.53 to 104.75) | 72.50 (55.61 to 93.87) | 68.98 (51.03 to 93.99) | 28.55 (21.72 to 39.25) | 27.18 (21.61 to 34.02) | 24.22 (18.57 to 31.35) | 23.52 (17.40 to 32.05) | -18 (-56 to 48) | -5 (-45 to 57) | -13 (-49 to 48) | -11 (-45 to 45) | -3 (-45 to 73) | 8,423.44 (6,406.41 to 11,581.53) | 7,507.02 (5,969.60 to 9,396.89) | 6,503.86 (4,989.13 to 8,421.62) | 6,187.92 (4,578.79 to 8,432.84) | 2,561.75 (1,948.33 to 3,522.19) | 2,438.30 (1,938.95 to 3,052.14) | 2,172.43 (1,666.48 to 2,813.00) | 2,109.76 (1,561.13 to 2,875.16) | -18 (-56 to 48) | -5 (-45 to 57) | -13 (-49 to 48) | -11 (-45 to 45) | -3 (-45 to 73) |
| **Honduras** | 32.32 (12.75 to 65.08) | 15.19 (4.31 to 33.15) | 14.64 (4.11 to 33.80) | 14.35 (3.91 to 33.30) | 18.78 (7.41 to 37.81) | 6.98 (1.98 to 15.22) | 6.71 (1.88 to 15.50) | 6.57 (1.79 to 15.24) | -65 (-95 to 106) | -63 (-95 to 105) | -6 (-88 to 671) | -4 (-88 to 683) | -2 (-88 to 709) | 2,900.31 (1,144.47 to 5,840.98) | 1,363.07 (386.74 to 2,972.74) | 1,313.86 (369.06 to 3,031.65) | 1,287.28 (351.58 to 2,987.07) | 1,685.25 (665.00 to 3,393.95) | 625.99 (177.61 to 1,365.23) | 602.39 (169.21 to 1,389.98) | 589.13 (160.90 to 1,367.05) | -65 (-95 to 106) | -63 (-95 to 105) | -6 (-88 to 671) | -4 (-88 to 683) | -2 (-88 to 709) |
| **United Mexican States** | 350.65 (308.07 to 405.46) | 454.00 (355.19 to 572.84) | 388.08 (286.45 to 520.86) | 368.04 (263.52 to 513.26) | 14.31 (12.57 to 16.54) | 22.59 (17.67 to 28.50) | 20.03 (14.78 to 26.88) | 19.61 (14.04 to 27.35) | 37 (-15 to 118) | 58 (7 to 127) | -13 (-51 to 55) | -11 (-48 to 52) | -2 (-48 to 85) | 31,463.41 (27,640.02 to 36,384.66) | 40,748.93 (31,880.55 to 51,413.18) | 34,820.25 (25,700.87 to 46,720.93) | 33,022.81 (23,650.50 to 46,040.73) | 1,283.72 (1,127.73 to 1,484.51) | 2,027.17 (1,585.98 to 2,557.69) | 1,796.74 (1,326.17 to 2,410.81) | 1,759.45 (1,260.09 to 2,453.04) | 37 (-15 to 118) | 58 (7 to 127) | -13 (-51 to 55) | -11 (-48 to 52) | -2 (-48 to 85) |
| **Nicaragua** | 24.24 (12.04 to 43.04) | 10.40 (3.26 to 19.87) | 9.92 (3.06 to 19.37) | 9.54 (2.76 to 18.45) | 17.78 (8.83 to 31.56) | 8.00 (2.51 to 15.29) | 7.73 (2.38 to 15.10) | 7.53 (2.18 to 14.56) | -58 (-93 to 65) | -55 (-92 to 73) | -6 (-86 to 480) | -3 (-84 to 501) | -3 (-86 to 511) | 2,175.70 (1,080.49 to 3,862.10) | 932.41 (292.88 to 1,780.34) | 889.72 (274.59 to 1,736.52) | 855.20 (248.08 to 1,653.53) | 1,595.61 (792.41 to 2,832.37) | 717.27 (225.30 to 1,369.54) | 693.63 (214.07 to 1,353.80) | 674.94 (195.79 to 1,304.99) | -58 (-93 to 65) | -55 (-92 to 73) | -6 (-86 to 480) | -3 (-84 to 501) | -3 (-86 to 511) |
| **Panama** | 1.52 (1.19 to 1.96) | 5.66 (4.18 to 7.48) | 5.12 (3.72 to 6.91) | 4.92 (3.44 to 6.73) | 2.64 (2.07 to 3.41) | 7.80 (5.75 to 10.29) | 7.18 (5.22 to 9.69) | 7.03 (4.91 to 9.63) | 166 (44 to 365) | 195 (69 to 398) | -10 (-52 to 67) | -8 (-49 to 69) | -2 (-49 to 84) | 136.70 (106.95 to 176.14) | 508.14 (374.66 to 670.87) | 458.99 (334.07 to 619.75) | 441.26 (308.42 to 604.29) | 237.21 (185.59 to 305.64) | 699.27 (515.60 to 923.22) | 643.99 (468.72 to 869.56) | 630.69 (440.83 to 863.72) | 166 (44 to 365) | 195 (69 to 398) | -10 (-52 to 67) | -8 (-49 to 69) | -2 (-49 to 84) |
| **Bolivarian Republic of Venezuela** | 14.50 (11.63 to 17.78) | 25.63 (17.02 to 38.65) | 21.87 (14.28 to 32.90) | 19.59 (12.90 to 30.25) | 2.72 (2.18 to 3.33) | 5.13 (3.41 to 7.74) | 4.80 (3.13 to 7.22) | 4.39 (2.89 to 6.78) | 62 (-13 to 211) | 89 (2 to 255) | -14 (-63 to 99) | -7 (-59 to 112) | -8 (-60 to 117) | 1,301.01 (1,043.83 to 1,595.51) | 2,298.86 (1,527.81 to 3,464.23) | 1,961.53 (1,281.32 to 2,949.91) | 1,757.59 (1,157.53 to 2,713.15) | 243.68 (195.51 to 298.85) | 460.31 (305.92 to 693.65) | 430.24 (281.04 to 647.03) | 394.16 (259.59 to 608.45) | 62 (-13 to 211) | 89 (2 to 255) | -14 (-63 to 99) | -7 (-59 to 112) | -8 (-60 to 117) |
| **Southern Latin America** | | | | | | | | | | | | | | | | | | | | | | | | | | |
| **Argentine Republic** | 456.38 (319.00 to 634.54) | 131.28 (99.71 to 172.59) | 103.53 (76.62 to 139.10) | 97.74 (67.88 to 137.61) | 67.64 (47.28 to 94.04) | 21.82 (16.58 to 28.69) | 18.72 (13.85 to 25.15) | 18.43 (12.80 to 25.94) | -73 (-86 to -45) | -68 (-82 to -39) | -16 (-55 to 57) | -14 (-52 to 52) | -2 (-49 to 87) | 40,954.59 (28,626.38 to 56,945.07) | 11,784.00 (8,950.04 to 15,490.79) | 9,293.24 (6,877.87 to 12,485.94) | 8,773.61 (6,093.45 to 12,352.72) | 6,069.60 (4,242.52 to 8,439.45) | 1,959.03 (1,487.90 to 2,575.27) | 1,680.17 (1,243.48 to 2,257.39) | 1,654.09 (1,148.80 to 2,328.86) | -73 (-86 to -45) | -68 (-82 to -39) | -16 (-55 to 57) | -14 (-52 to 52) | -2 (-49 to 87) |
| **Republic of Chile** | 78.15 (64.58 to 94.54) | 21.80 (16.53 to 27.20) | 15.73 (12.41 to 19.71) | 14.62 (10.80 to 18.49) | 26.18 (21.63 to 31.67) | 10.25 (7.78 to 12.80) | 7.62 (6.01 to 9.55) | 7.23 (5.34 to 9.15) | -72 (-83 to -58) | -61 (-75 to -41) | -29 (-58 to 18) | -26 (-53 to 23) | -5 (-44 to 52) | 7,014.51 (5,796.68 to 8,485.62) | 1,956.56 (1,484.04 to 2,441.84) | 1,411.54 (1,113.58 to 1,769.48) | 1,312.37 (969.49 to 1,660.09) | 2,349.62 (1,941.68 to 2,842.39) | 920.32 (698.05 to 1,148.58) | 683.87 (539.52 to 857.29) | 649.00 (479.44 to 820.96) | -72 (-83 to -58) | -61 (-75 to -41) | -29 (-58 to 18) | -26 (-53 to 23) | -5 (-44 to 52) |
| **Uruguay** | 18.21 (14.68 to 22.43) | 6.95 (5.32 to 9.04) | 6.50 (4.85 to 8.53) | 6.01 (4.20 to 8.33) | 33.14 (26.72 to 40.83) | 18.37 (14.06 to 23.87) | 17.93 (13.37 to 23.52) | 16.98 (11.86 to 23.52) | -49 (-71 to -12) | -45 (-66 to -11) | -8 (-50 to 67) | -2 (-44 to 67) | -5 (-50 to 76) | 1,633.96 (1,317.20 to 2,013.11) | 624.07 (477.76 to 811.20) | 583.54 (435.23 to 765.57) | 539.95 (377.15 to 747.87) | 2,974.28 (2,397.67 to 3,664.43) | 1,648.64 (1,262.13 to 2,143.02) | 1,609.19 (1,200.22 to 2,111.17) | 1,524.49 (1,064.85 to 2,111.54) | -49 (-71 to -12) | -45 (-66 to -11) | -8 (-50 to 67) | -2 (-44 to 67) | -5 (-50 to 76) |
| **Tropical Latin America** | | | | | | | | | | | | | | | | | | | | | | | | | | |
| **Brazil** | 187.47 (150.67 to 220.39) | 144.29 (113.75 to 185.72) | 119.34 (91.83 to 153.78) | 113.12 (85.96 to 143.92) | 5.98 (4.80 to 7.02) | 4.27 (3.37 to 5.50) | 3.58 (2.75 to 4.61) | 3.44 (2.61 to 4.38) | -42 (-63 to -9) | -29 (-52 to 14) | -19 (-52 to 30) | -16 (-50 to 37) | -4 (-43 to 59) | 16,820.08 (13,517.49 to 19,774.20) | 12,948.70 (10,207.62 to 16,666.52) | 10,708.66 (8,240.23 to 13,799.24) | 10,150.94 (7,712.45 to 12,914.19) | 536.14 (430.87 to 630.30) | 383.30 (302.16 to 493.35) | 320.89 (246.92 to 413.50) | 308.79 (234.61 to 392.84) | -42 (-63 to -9) | -29 (-52 to 14) | -19 (-52 to 30) | -16 (-50 to 37) | -4 (-43 to 59) |
| **Paraguay** | 9.73 (1.87 to 22.45) | 5.84 (1.76 to 12.08) | 5.50 (1.59 to 10.95) | 5.57 (1.50 to 11.00) | 7.74 (1.49 to 17.87) | 4.51 (1.36 to 9.32) | 4.28 (1.24 to 8.53) | 4.37 (1.18 to 8.64) | -44 (-93 to 482) | -42 (-92 to 528) | -3 (-87 to 535) | -5 (-87 to 527) | 2 (-86 to 599) | 872.97 (167.47 to 2,014.23) | 523.91 (158.04 to 1,084.03) | 493.61 (142.41 to 982.52) | 499.55 (134.22 to 986.85) | 694.74 (133.28 to 1,602.98) | 404.23 (121.94 to 836.40) | 384.30 (110.87 to 764.94) | 392.28 (105.40 to 774.95) | -44 (-93 to 482) | -42 (-92 to 528) | -3 (-87 to 535) | -5 (-87 to 527) | 2 (-86 to 599) |
| **North Africa and Middel East** | | | | | | | | | | | | | | | | | | | | | | | | | | |
| **Afghanistan** | 773.87 (373.56 to 1,467.84) | 903.38 (462.17 to 1,494.08) | 903.16 (460.97 to 1,489.58) | 897.17 (441.97 to 1,520.09) | 193.04 (93.18 to 366.14) | 81.62 (41.76 to 134.99) | 79.57 (40.61 to 131.24) | 77.12 (37.99 to 130.67) | -60 (-90 to 40) | -58 (-89 to 45) | -6 (-72 to 213) | -3 (-70 to 214) | -3 (-71 to 222) | 69,444.73 (33,523.11 to 131,750.84) | 81,051.41 (41,473.80 to 134,010.24) | 81,032.12 (41,372.53 to 133,687.79) | 80,496.55 (39,650.84 to 136,428.93) | 17,322.54 (8,362.12 to 32,864.39) | 7,323.15 (3,747.24 to 12,108.08) | 7,139.45 (3,645.18 to 11,778.75) | 6,919.54 (3,408.42 to 11,727.53) | -60 (-90 to 40) | -58 (-89 to 45) | -6 (-72 to 213) | -3 (-70 to 214) | -3 (-71 to 222) |
| **People's Democratic Republic of Algeria** | 391.45 (132.85 to 921.68) | 219.85 (111.43 to 366.23) | 202.02 (104.64 to 329.51) | 190.03 (97.49 to 310.75) | 52.71 (17.89 to 124.10) | 22.99 (11.65 to 38.30) | 21.67 (11.23 to 35.35) | 20.99 (10.77 to 34.33) | -60 (-91 to 92) | -56 (-91 to 114) | -9 (-72 to 195) | -6 (-71 to 203) | -3 (-70 to 206) | 35,121.70 (11,921.71 to 82,685.69) | 19,725.81 (9,997.73 to 32,865.38) | 18,126.38 (9,388.19 to 29,564.93) | 17,050.48 (8,745.17 to 27,881.24) | 4,729.09 (1,605.24 to 11,133.51) | 2,062.86 (1,045.53 to 3,436.95) | 1,944.65 (1,007.19 to 3,171.81) | 1,883.72 (966.16 to 3,080.29) | -60 (-91 to 92) | -56 (-91 to 114) | -9 (-72 to 195) | -6 (-71 to 203) | -3 (-70 to 206) |
| **Kingdom of Bahrain** | 3.86 (1.96 to 6.80) | 2.41 (1.32 to 3.92) | 2.19 (1.19 to 3.57) | 2.09 (1.20 to 3.34) | 29.84 (15.17 to 52.57) | 12.85 (7.03 to 20.85) | 12.00 (6.53 to 19.58) | 11.83 (6.83 to 18.93) | -60 (-87 to 25) | -57 (-87 to 37) | -8 (-67 to 169) | -7 (-69 to 178) | -1 (-65 to 190) | 346.19 (176.07 to 610.05) | 216.59 (118.58 to 351.59) | 196.25 (106.88 to 320.28) | 187.29 (108.08 to 299.54) | 2,676.97 (1,361.52 to 4,717.26) | 1,152.81 (631.14 to 1,871.31) | 1,076.77 (586.40 to 1,757.31) | 1,061.88 (612.78 to 1,698.28) | -60 (-87 to 25) | -57 (-87 to 37) | -8 (-67 to 169) | -7 (-69 to 178) | -1 (-65 to 190) |
| **Egypt** | 1,942.73 (946.53 to 3,487.63) | 729.62 (361.35 to 1,257.90) | 629.49 (308.03 to 1,053.01) | 600.28 (303.40 to 1,003.50) | 107.64 (52.44 to 193.23) | 26.68 (13.21 to 45.99) | 23.81 (11.65 to 39.83) | 23.38 (11.82 to 39.09) | -78 (-94 to -25) | -75 (-93 to -12) | -12 (-74 to 196) | -11 (-75 to 201) | -2 (-70 to 236) | 174,311.05 (84,939.87 to 312,908.32) | 65,474.83 (32,423.52 to 112,863.94) | 56,492.01 (27,648.64 to 94,481.74) | 53,871.88 (27,234.12 to 90,044.36) | 9,657.84 (4,706.16 to 17,336.92) | 2,393.94 (1,185.50 to 4,126.62) | 2,136.71 (1,045.76 to 3,573.61) | 2,098.65 (1,060.94 to 3,507.80) | -78 (-94 to -25) | -75 (-93 to -12) | -12 (-74 to 196) | -11 (-75 to 201) | -2 (-70 to 236) |
| **Iran** | 1,133.93 (575.98 to 2,052.55) | 271.37 (159.75 to 408.27) | 128.02 (73.08 to 199.25) | 73.13 (40.65 to 122.73) | 73.73 (37.45 to 133.46) | 22.20 (13.07 to 33.41) | 11.32 (6.46 to 17.61) | 6.94 (3.86 to 11.65) | -91 (-97 to -69) | -70 (-90 to -11) | -69 (-88 to -11) | -49 (-81 to 35) | -39 (-78 to 80) | 101,737.85 (51,684.03 to 184,122.55) | 24,349.94 (14,334.37 to 36,634.65) | 11,487.80 (6,558.38 to 17,881.52) | 6,562.71 (3,648.04 to 11,014.51) | 6,615.06 (3,360.53 to 11,971.77) | 1,992.47 (1,172.93 to 2,997.68) | 1,015.48 (579.74 to 1,580.66) | 622.86 (346.23 to 1,045.38) | -91 (-97 to -69) | -70 (-90 to -11) | -69 (-88 to -11) | -49 (-81 to 35) | -39 (-78 to 80) |
| **Iraq** | 512.73 (262.82 to 866.56) | 290.86 (150.96 to 485.07) | 264.04 (133.87 to 449.23) | 249.02 (124.37 to 419.56) | 77.50 (39.73 to 130.99) | 33.76 (17.52 to 56.31) | 31.37 (15.90 to 53.37) | 30.18 (15.07 to 50.85) | -61 (-88 to 28) | -56 (-87 to 42) | -11 (-73 to 190) | -7 (-72 to 205) | -4 (-72 to 220) | 46,004.54 (23,581.08 to 77,758.30) | 26,096.08 (13,542.63 to 43,517.50) | 23,689.57 (12,011.41 to 40,303.92) | 22,342.58 (11,158.00 to 37,647.66) | 6,954.07 (3,564.53 to 11,753.98) | 3,029.12 (1,571.97 to 5,051.32) | 2,814.48 (1,427.03 to 4,788.37) | 2,708.15 (1,352.47 to 4,563.29) | -61 (-88 to 28) | -56 (-87 to 42) | -11 (-73 to 190) | -7 (-72 to 205) | -4 (-72 to 220) |
| **Hashemite Kingdom of Jordan** | 43.99 (21.26 to 77.06) | 36.48 (20.17 to 58.54) | 34.65 (19.40 to 55.83) | 34.64 (19.47 to 56.67) | 34.59 (16.71 to 60.59) | 17.11 (9.46 to 27.46) | 16.36 (9.16 to 26.35) | 16.39 (9.21 to 26.81) | -53 (-85 to 60) | -51 (-84 to 64) | -4 (-66 to 183) | -4 (-67 to 179) | 0 (-65 to 193) | 3,947.35 (1,907.63 to 6,914.27) | 3,273.63 (1,809.91 to 5,252.60) | 3,109.37 (1,740.59 to 5,010.55) | 3,108.07 (1,746.84 to 5,084.19) | 3,103.79 (1,499.97 to 5,436.68) | 1,535.42 (848.90 to 2,463.60) | 1,467.69 (821.60 to 2,365.08) | 1,470.40 (826.42 to 2,405.29) | -53 (-85 to 60) | -51 (-84 to 64) | -4 (-66 to 183) | -4 (-67 to 179) | 0 (-65 to 193) |
| **State of Kuwait** | 9.74 (6.90 to 12.79) | 13.65 (10.38 to 17.49) | 11.69 (9.04 to 14.77) | 11.40 (8.42 to 15.12) | 27.30 (19.34 to 35.85) | 25.85 (19.64 to 33.10) | 22.84 (17.66 to 28.86) | 22.88 (16.89 to 30.34) | -16 (-53 to 57) | -5 (-45 to 71) | -11 (-49 to 54) | -12 (-47 to 47) | 0 (-41 to 72) | 873.91 (619.32 to 1,147.71) | 1,225.28 (931.26 to 1,569.40) | 1,048.77 (810.92 to 1,325.36) | 1,023.17 (755.27 to 1,356.80) | 2,449.21 (1,735.71 to 3,216.56) | 2,319.36 (1,762.79 to 2,970.74) | 2,049.49 (1,584.69 to 2,590.01) | 2,053.33 (1,515.71 to 2,722.88) | -16 (-53 to 57) | -5 (-45 to 71) | -11 (-49 to 54) | -12 (-47 to 47) | 0 (-41 to 72) |
| **Lebanese Republic** | 22.64 (9.32 to 43.35) | 13.83 (6.62 to 24.68) | 12.31 (5.80 to 22.54) | 11.56 (5.62 to 22.13) | 27.29 (11.23 to 52.24) | 15.84 (7.59 to 28.28) | 14.81 (6.98 to 27.13) | 14.56 (7.08 to 27.88) | -47 (-86 to 148) | -42 (-85 to 152) | -8 (-75 to 267) | -7 (-75 to 257) | -2 (-74 to 300) | 2,031.87 (836.15 to 3,889.78) | 1,240.66 (594.21 to 2,213.93) | 1,104.36 (520.18 to 2,022.73) | 1,037.49 (504.62 to 1,985.77) | 2,448.46 (1,007.59 to 4,687.30) | 1,421.82 (680.97 to 2,537.21) | 1,329.00 (625.99 to 2,434.19) | 1,306.79 (635.60 to 2,501.22) | -47 (-86 to 148) | -42 (-85 to 152) | -8 (-75 to 267) | -7 (-75 to 257) | -2 (-74 to 300) |
| **State of Libya** | 36.68 (14.55 to 81.56) | 16.22 (7.01 to 29.90) | 15.00 (6.63 to 27.24) | 14.18 (6.36 to 25.53) | 28.66 (11.37 to 63.74) | 19.04 (8.23 to 35.09) | 18.29 (8.09 to 33.22) | 17.92 (8.04 to 32.26) | -37 (-87 to 184) | -34 (-87 to 209) | -6 (-77 to 292) | -4 (-77 to 303) | -2 (-76 to 299) | 3,291.08 (1,305.95 to 7,317.33) | 1,455.59 (629.58 to 2,681.90) | 1,345.57 (595.25 to 2,443.60) | 1,272.37 (570.91 to 2,290.44) | 2,571.82 (1,020.54 to 5,718.14) | 1,708.26 (738.86 to 3,147.44) | 1,640.83 (725.86 to 2,979.80) | 1,607.66 (721.35 to 2,894.01) | -37 (-87 to 184) | -34 (-87 to 209) | -6 (-77 to 292) | -4 (-77 to 303) | -2 (-76 to 299) |
| **Morocco** | 1,220.49 (619.75 to 2,450.15) | 238.02 (113.15 to 413.30) | 213.89 (101.90 to 372.06) | 197.89 (92.89 to 346.37) | 162.17 (82.35 to 325.56) | 35.13 (16.70 to 61.00) | 32.60 (15.53 to 56.70) | 31.12 (14.61 to 54.46) | -81 (-96 to -34) | -78 (-95 to -26) | -11 (-76 to 226) | -7 (-75 to 240) | -5 (-74 to 251) | 109,516.63 (55,609.66 to 219,887.07) | 21,359.43 (10,153.03 to 37,078.26) | 19,193.90 (9,143.30 to 33,386.56) | 17,758.96 (8,336.59 to 31,080.28) | 14,551.82 (7,389.03 to 29,217.09) | 3,152.44 (1,498.49 to 5,472.38) | 2,925.10 (1,393.42 to 5,088.03) | 2,792.38 (1,310.83 to 4,886.99) | -81 (-96 to -34) | -78 (-95 to -26) | -11 (-76 to 226) | -7 (-75 to 240) | -5 (-74 to 251) |
| **Oman** | 26.71 (14.10 to 44.72) | 16.04 (8.53 to 25.55) | 14.78 (8.02 to 23.71) | 13.71 (7.20 to 21.96) | 38.23 (20.19 to 64.01) | 19.80 (10.53 to 31.52) | 18.33 (9.95 to 29.41) | 17.15 (9.01 to 27.48) | -55 (-86 to 36) | -48 (-84 to 56) | -13 (-71 to 161) | -7 (-68 to 179) | -6 (-69 to 176) | 2,396.14 (1,265.60 to 4,011.70) | 1,439.64 (765.36 to 2,292.41) | 1,326.15 (719.78 to 2,127.49) | 1,230.01 (645.77 to 1,970.54) | 3,430.15 (1,811.75 to 5,742.87) | 1,776.38 (944.38 to 2,828.61) | 1,645.30 (893.00 to 2,639.48) | 1,539.17 (808.09 to 2,465.83) | -55 (-86 to 36) | -48 (-84 to 56) | -13 (-71 to 161) | -7 (-68 to 179) | -6 (-69 to 176) |
| **Palestine** | 23.95 (8.31 to 50.42) | 24.74 (13.39 to 41.20) | 23.07 (12.40 to 38.08) | 22.44 (11.67 to 37.57) | 28.43 (9.86 to 59.85) | 20.18 (10.92 to 33.60) | 19.10 (10.26 to 31.52) | 18.82 (9.79 to 31.51) | -34 (-84 to 220) | -29 (-82 to 241) | -7 (-71 to 188) | -5 (-69 to 189) | -1 (-69 to 207) | 2,148.98 (745.34 to 4,523.77) | 2,219.93 (1,201.47 to 3,697.05) | 2,070.30 (1,112.56 to 3,416.88) | 2,013.39 (1,047.46 to 3,371.63) | 2,550.83 (884.72 to 5,369.70) | 1,810.57 (979.92 to 3,015.31) | 1,713.89 (921.03 to 2,828.63) | 1,688.69 (878.54 to 2,827.89) | -34 (-84 to 220) | -29 (-82 to 241) | -7 (-71 to 188) | -5 (-69 to 189) | -1 (-69 to 207) |
| **Qatar** | 2.63 (1.04 to 4.84) | 3.68 (1.72 to 6.66) | 3.61 (1.65 to 6.69) | 3.32 (1.49 to 6.42) | 23.64 (9.37 to 43.44) | 9.95 (4.66 to 18.00) | 9.54 (4.37 to 17.70) | 8.61 (3.88 to 16.67) | -64 (-91 to 78) | -58 (-89 to 92) | -13 (-78 to 257) | -4 (-76 to 280) | -10 (-78 to 281) | 236.18 (93.63 to 433.99) | 330.35 (154.76 to 597.49) | 323.79 (148.41 to 600.50) | 297.53 (133.92 to 575.91) | 2,121.30 (840.92 to 3,897.98) | 893.11 (418.40 to 1,615.30) | 856.59 (392.61 to 1,588.61) | 772.90 (347.89 to 1,496.02) | -64 (-91 to 78) | -58 (-89 to 92) | -13 (-78 to 257) | -4 (-76 to 280) | -10 (-78 to 281) |
| **Kingdom of Saudi Arabia** | 199.45 (87.44 to 372.83) | 55.01 (22.53 to 105.54) | 47.50 (18.02 to 88.10) | 44.97 (16.84 to 85.32) | 40.38 (17.70 to 75.49) | 11.28 (4.62 to 21.63) | 9.98 (3.79 to 18.51) | 9.69 (3.63 to 18.39) | -76 (-95 to 4) | -72 (-94 to 22) | -14 (-83 to 298) | -12 (-83 to 301) | -3 (-80 to 386) | 17,896.36 (7,848.62 to 33,453.13) | 4,937.02 (2,021.69 to 9,472.45) | 4,262.88 (1,617.13 to 7,907.31) | 4,036.23 (1,510.65 to 7,657.81) | 3,623.59 (1,589.16 to 6,773.47) | 1,012.01 (414.41 to 1,941.70) | 895.47 (339.70 to 1,661.03) | 869.87 (325.57 to 1,650.37) | -76 (-95 to 4) | -72 (-94 to 22) | -14 (-83 to 298) | -12 (-83 to 301) | -3 (-80 to 386) |
| **Sudan** | 1,205.81 (584.81 to 2,416.50) | 784.70 (358.94 to 1,597.46) | 726.24 (350.37 to 1,487.07) | 678.43 (326.59 to 1,425.55) | 148.94 (72.23 to 298.47) | 67.55 (30.90 to 137.52) | 63.59 (30.68 to 130.20) | 60.26 (29.01 to 126.62) | -60 (-90 to 75) | -55 (-90 to 90) | -11 (-79 to 310) | -6 (-78 to 321) | -5 (-78 to 313) | 108,194.03 (52,492.50 to 216,855.45) | 70,392.60 (32,204.05 to 143,300.57) | 65,148.41 (31,439.52 to 133,388.34) | 60,860.94 (29,290.49 to 127,906.70) | 13,363.48 (6,483.56 to 26,784.69) | 6,059.78 (2,772.30 to 12,336.10) | 5,704.12 (2,752.71 to 11,678.92) | 5,405.73 (2,601.61 to 11,360.79) | -60 (-90 to 75) | -55 (-90 to 90) | -11 (-79 to 310) | -6 (-78 to 321) | -5 (-78 to 313) |
| **Syrian Arab Republic** | 404.15 (208.82 to 724.02) | 35.38 (12.44 to 70.84) | 31.20 (11.23 to 59.95) | 29.51 (10.06 to 56.89) | 91.03 (47.03 to 163.08) | 17.40 (6.12 to 34.84) | 15.62 (5.62 to 30.02) | 15.13 (5.16 to 29.17) | -83 (-97 to -38) | -81 (-96 to -26) | -13 (-85 to 377) | -10 (-84 to 391) | -3 (-83 to 419) | 36,260.97 (18,731.91 to 64,958.91) | 3,174.76 (1,116.29 to 6,356.82) | 2,800.18 (1,008.00 to 5,379.56) | 2,648.07 (903.27 to 5,105.20) | 8,167.52 (4,219.23 to 14,631.53) | 1,561.45 (549.03 to 3,126.49) | 1,402.06 (504.71 to 2,693.56) | 1,357.83 (463.16 to 2,617.76) | -83 (-97 to -38) | -81 (-96 to -26) | -13 (-85 to 377) | -10 (-84 to 391) | -3 (-83 to 419) |
| **Tunisia** | 126.69 (53.03 to 253.22) | 33.12 (17.73 to 55.55) | 29.79 (15.75 to 50.68) | 28.07 (14.82 to 48.20) | 59.52 (24.91 to 118.96) | 18.38 (9.84 to 30.83) | 17.14 (9.06 to 29.16) | 16.77 (8.85 to 28.80) | -72 (-93 to 16) | -69 (-92 to 24) | -9 (-71 to 193) | -7 (-71 to 196) | -2 (-70 to 218) | 11,366.71 (4,758.25 to 22,717.99) | 2,972.51 (1,591.11 to 4,985.03) | 2,673.46 (1,412.98 to 4,547.94) | 2,519.32 (1,330.32 to 4,326.15) | 5,340.05 (2,235.41 to 10,672.85) | 1,649.85 (883.13 to 2,766.88) | 1,537.99 (812.86 to 2,616.34) | 1,504.99 (794.71 to 2,584.36) | -72 (-93 to 16) | -69 (-92 to 24) | -9 (-71 to 193) | -7 (-71 to 196) | -2 (-70 to 218) |
| **Turkey** | 1,120.53 (399.88 to 2,230.69) | 221.71 (115.44 to 366.20) | 194.58 (105.91 to 312.02) | 179.28 (96.91 to 290.95) | 78.72 (28.09 to 156.72) | 19.99 (10.41 to 33.01) | 18.38 (10.00 to 29.47) | 17.69 (9.56 to 28.71) | -78 (-94 to 2) | -75 (-93 to 18) | -11 (-71 to 176) | -8 (-70 to 183) | -4 (-68 to 187) | 100,534.69 (35,883.56 to 200,057.29) | 19,896.39 (10,360.19 to 32,864.25) | 17,461.99 (9,505.13 to 28,005.04) | 16,088.32 (8,696.69 to 26,114.00) | 7,063.10 (2,521.01 to 14,055.10) | 1,793.70 (934.00 to 2,962.79) | 1,649.19 (897.71 to 2,644.92) | 1,587.55 (858.16 to 2,576.85) | -78 (-94 to 2) | -75 (-93 to 18) | -11 (-71 to 176) | -8 (-70 to 183) | -4 (-68 to 187) |
| **United Arab Emirates** | 11.03 (5.69 to 19.45) | 9.22 (4.82 to 15.94) | 8.42 (4.20 to 14.86) | 7.19 (3.52 to 12.93) | 23.26 (12.00 to 41.01) | 10.63 (5.55 to 18.37) | 10.37 (5.17 to 18.30) | 9.44 (4.62 to 16.96) | -59 (-89 to 41) | -54 (-86 to 53) | -11 (-75 to 206) | -2 (-72 to 230) | -9 (-75 to 228) | 989.94 (510.73 to 1,745.10) | 827.20 (432.10 to 1,430.35) | 755.89 (377.07 to 1,332.92) | 645.43 (315.96 to 1,160.23) | 2,087.52 (1,076.99 to 3,679.95) | 953.42 (498.03 to 1,648.59) | 930.78 (464.32 to 1,641.33) | 846.81 (414.55 to 1,522.23) | -59 (-89 to 41) | -54 (-86 to 53) | -11 (-75 to 206) | -2 (-72 to 230) | -9 (-75 to 228) |
| **Yemen** | 985.33 (484.38 to 1,715.60) | 830.00 (363.86 to 1,581.87) | 788.69 (344.98 to 1,520.19) | 753.83 (338.89 to 1,519.31) | 162.47 (79.87 to 282.89) | 86.29 (37.83 to 164.45) | 82.88 (36.25 to 159.76) | 79.69 (35.82 to 160.61) | -51 (-87 to 101) | -47 (-87 to 106) | -8 (-78 to 325) | -4 (-78 to 322) | -4 (-78 to 343) | 88,411.46 (43,464.71 to 153,971.96) | 74,460.33 (32,644.25 to 141,897.68) | 70,754.83 (30,935.08 to 136,409.52) | 67,627.41 (30,389.93 to 136,349.23) | 14,578.26 (7,166.94 to 25,388.60) | 7,741.07 (3,393.77 to 14,752.02) | 7,435.65 (3,250.98 to 14,335.33) | 7,148.93 (3,212.54 to 14,413.56) | -51 (-87 to 101) | -47 (-87 to 106) | -8 (-78 to 325) | -4 (-78 to 322) | -4 (-78 to 343) |
| **High-income North America** | | | | | | | | | | | | | | | | | | | | | | | | | | |
| **Canada** | 325.34 (289.82 to 361.22) | 23.98 (17.82 to 31.69) | 23.23 (16.61 to 31.01) | 20.68 (14.11 to 29.03) | 81.96 (73.01 to 91.00) | 6.45 (4.79 to 8.52) | 6.33 (4.53 to 8.45) | 5.65 (3.86 to 7.93) | -93 (-96 to -89) | -92 (-95 to -88) | -12 (-55 to 66) | -2 (-47 to 76) | -11 (-54 to 75) | 29,193.13 (26,004.95 to 32,414.33) | 2,151.55 (1,599.12 to 2,844.34) | 2,084.64 (1,490.57 to 2,782.67) | 1,855.61 (1,266.64 to 2,605.38) | 7,354.28 (6,551.12 to 8,165.75) | 578.71 (430.12 to 765.06) | 568.17 (406.25 to 758.42) | 507.22 (346.23 to 712.17) | -93 (-96 to -89) | -92 (-95 to -88) | -12 (-55 to 66) | -2 (-47 to 76) | -11 (-54 to 75) |
| **Greenland** | 1.60 (0.64 to 3.12) | 0.42 (0.24 to 0.68) | 0.44 (0.24 to 0.71) | 0.38 (0.20 to 0.61) | 136.78 (54.21 to 265.83) | 51.25 (28.74 to 83.24) | 55.52 (30.38 to 89.30) | 48.51 (26.30 to 79.31) | -65 (-90 to 46) | -63 (-89 to 54) | -5 (-68 to 176) | 8 (-64 to 211) | -13 (-71 to 161) | 143.98 (57.08 to 279.76) | 37.68 (21.13 to 61.20) | 39.72 (21.73 to 63.89) | 33.69 (18.26 to 55.07) | 12,272.26 (4,865.10 to 23,845.29) | 4,598.70 (2,579.43 to 7,469.81) | 4,982.39 (2,726.10 to 8,013.77) | 4,353.47 (2,360.30 to 7,117.16) | -65 (-90 to 46) | -63 (-89 to 54) | -5 (-68 to 176) | 8 (-64 to 211) | -13 (-71 to 161) |
| **United States of America** | 5,195.05 (4,974.84 to 5,404.35) | 1,297.06 (1,192.37 to 1,404.36) | 1,223.53 (1,089.26 to 1,365.80) | 1,173.66 (995.93 to 1,354.42) | 126.88 (121.51 to 132.00) | 34.90 (32.08 to 37.79) | 33.40 (29.74 to 37.29) | 32.12 (27.26 to 37.07) | -75 (-79 to -69) | -72 (-76 to -69) | -8 (-28 to 16) | -4 (-21 to 16) | -4 (-27 to 25) | 466,176.93 (446,414.11 to 484,957.34) | 116,381.75 (106,986.56 to 126,011.60) | 109,788.46 (97,738.11 to 122,553.70) | 105,314.81 (89,369.39 to 121,542.28) | 11,385.99 (10,903.30 to 11,844.68) | 3,131.56 (2,878.76 to 3,390.68) | 2,997.33 (2,668.34 to 3,345.83) | 2,882.36 (2,445.95 to 3,326.49) | -75 (-79 to -69) | -72 (-76 to -69) | -8 (-28 to 16) | -4 (-21 to 16) | -4 (-27 to 25) |
| **Oceania** | | | | | | | | | | | | | | | | | | | | | | | | | | |
| **American Samoa** | 0.27 (0.11 to 0.49) | 0.08 (0.04 to 0.15) | 0.08 (0.04 to 0.15) | 0.07 (0.03 to 0.13) | 15.91 (6.21 to 28.73) | 11.18 (5.45 to 20.00) | 11.92 (5.71 to 21.75) | 10.86 (4.97 to 19.85) | -32 (-83 to 220) | -30 (-81 to 222) | -3 (-75 to 264) | 7 (-71 to 299) | -9 (-77 to 247) | 24.33 (9.50 to 43.93) | 7.43 (3.63 to 13.30) | 7.47 (3.58 to 13.63) | 6.48 (2.96 to 11.85) | 1,427.70 (557.37 to 2,577.80) | 1,002.69 (489.26 to 1,794.57) | 1,069.38 (512.67 to 1,951.64) | 973.99 (445.75 to 1,781.21) | -32 (-83 to 220) | -30 (-81 to 222) | -3 (-75 to 264) | 7 (-71 to 299) | -9 (-77 to 247) |
| **Cook Islands** | 0.08 (0.03 to 0.16) | 0.01 (0.00 to 0.02) | 0.02 (0.00 to 0.05) | 0.03 (0.00 to 0.05) | 19.39 (7.20 to 36.41) | 4.35 (0.79 to 9.52) | 11.25 (2.16 to 23.72) | 11.71 (2.18 to 24.54) | -40 (-94 to 241) | -78 (-98 to 32) | 169 (-77 to 3,022) | 158 (-77 to 2,916) | 4 (-91 to 1,034) | 7.54 (2.80 to 14.16) | 0.88 (0.16 to 1.91) | 2.23 (0.43 to 4.70) | 2.30 (0.43 to 4.81) | 1,739.62 (646.06 to 3,267.68) | 390.80 (70.54 to 854.59) | 1,008.76 (194.17 to 2,127.08) | 1,050.70 (195.32 to 2,200.51) | -40 (-94 to 241) | -78 (-98 to 32) | 169 (-77 to 3,022) | 158 (-77 to 2,916) | 4 (-91 to 1,034) |
| **Fiji** | 2.97 (1.02 to 6.00) | 1.97 (0.86 to 3.72) | 1.87 (0.84 to 3.52) | 1.83 (0.82 to 3.30) | 16.11 (5.51 to 32.47) | 10.67 (4.67 to 20.16) | 10.26 (4.61 to 19.39) | 10.22 (4.58 to 18.45) | -37 (-86 to 235) | -34 (-86 to 266) | -4 (-77 to 295) | -4 (-77 to 315) | 0 (-76 to 300) | 266.85 (91.23 to 537.97) | 176.72 (77.42 to 333.72) | 167.37 (75.27 to 316.15) | 164.21 (73.66 to 296.42) | 1,445.25 (494.10 to 2,913.65) | 957.57 (419.50 to 1,808.31) | 920.87 (414.11 to 1,739.44) | 916.91 (411.33 to 1,655.15) | -37 (-86 to 235) | -34 (-86 to 266) | -4 (-77 to 295) | -4 (-77 to 315) | 0 (-76 to 300) |
| **Guam** | 0.64 (0.25 to 1.13) | 0.36 (0.18 to 0.63) | 0.31 (0.16 to 0.56) | 0.30 (0.15 to 0.53) | 17.50 (6.94 to 31.00) | 12.85 (6.54 to 22.77) | 11.60 (5.87 to 20.71) | 11.29 (5.66 to 20.26) | -35 (-82 to 192) | -27 (-79 to 228) | -12 (-75 to 210) | -10 (-74 to 217) | -3 (-73 to 245) | 57.24 (22.70 to 101.38) | 32.07 (16.32 to 56.85) | 28.06 (14.19 to 50.09) | 26.59 (13.32 to 47.73) | 1,570.45 (622.73 to 2,781.71) | 1,152.64 (586.53 to 2,043.49) | 1,041.20 (526.48 to 1,858.46) | 1,013.24 (507.63 to 1,818.56) | -35 (-82 to 192) | -27 (-79 to 228) | -12 (-75 to 210) | -10 (-74 to 217) | -3 (-73 to 245) |
| **Kiribati** | 1.10 (0.34 to 2.64) | 0.52 (0.22 to 0.96) | 0.51 (0.20 to 0.92) | 0.50 (0.20 to 0.89) | 42.50 (13.11 to 102.51) | 17.98 (7.49 to 33.52) | 17.73 (7.09 to 31.99) | 17.43 (6.88 to 31.11) | -59 (-93 to 137) | -58 (-93 to 156) | -3 (-79 to 315) | -1 (-79 to 327) | -2 (-78 to 339) | 98.34 (30.33 to 237.09) | 46.35 (19.31 to 86.38) | 45.58 (18.24 to 82.23) | 44.73 (17.65 to 79.81) | 3,813.28 (1,176.24 to 9,193.55) | 1,613.09 (672.25 to 3,006.43) | 1,590.23 (636.25 to 2,869.02) | 1,563.84 (617.27 to 2,790.43) | -59 (-93 to 137) | -58 (-93 to 156) | -3 (-79 to 315) | -1 (-79 to 327) | -2 (-78 to 339) |
| **Marshall Islands** | 0.37 (0.14 to 0.70) | 0.14 (0.06 to 0.27) | 0.14 (0.06 to 0.26) | 0.14 (0.06 to 0.25) | 25.18 (9.90 to 47.65) | 12.29 (5.41 to 23.42) | 12.28 (5.60 to 22.80) | 12.02 (5.54 to 22.12) | -52 (-88 to 124) | -51 (-89 to 137) | -2 (-76 to 309) | 0 (-76 to 321) | -2 (-76 to 295) | 33.01 (12.97 to 62.45) | 12.85 (5.66 to 24.48) | 12.63 (5.76 to 23.44) | 12.17 (5.61 to 22.41) | 2,259.52 (887.98 to 4,275.02) | 1,103.19 (485.55 to 2,101.10) | 1,101.91 (502.48 to 2,045.27) | 1,078.35 (496.99 to 1,985.04) | -52 (-88 to 124) | -51 (-89 to 137) | -2 (-76 to 309) | 0 (-76 to 321) | -2 (-76 to 295) |
| **Federated States of Micronesia** | 1.04 (0.42 to 1.89) | 0.22 (0.12 to 0.41) | 0.21 (0.11 to 0.40) | 0.21 (0.11 to 0.37) | 33.77 (13.59 to 61.34) | 11.86 (6.14 to 21.77) | 11.41 (5.92 to 21.44) | 11.21 (5.70 to 20.17) | -67 (-91 to 48) | -65 (-90 to 60) | -5 (-74 to 229) | -4 (-73 to 249) | -2 (-73 to 241) | 93.36 (37.57 to 169.54) | 20.17 (10.44 to 37.02) | 19.13 (9.92 to 35.96) | 18.56 (9.44 to 33.39) | 3,030.07 (1,219.19 to 5,502.26) | 1,064.15 (550.93 to 1,952.99) | 1,023.68 (531.12 to 1,924.61) | 1,006.09 (511.58 to 1,810.46) | -67 (-91 to 48) | -65 (-90 to 60) | -5 (-74 to 229) | -4 (-73 to 249) | -2 (-73 to 241) |
| **Nauru** | 0.07 (0.02 to 0.15) | 0.04 (0.02 to 0.08) | 0.04 (0.02 to 0.08) | 0.04 (0.02 to 0.08) | 20.34 (6.71 to 42.63) | 14.32 (5.66 to 27.54) | 14.09 (5.68 to 27.41) | 14.02 (5.74 to 26.61) | -31 (-87 to 296) | -30 (-87 to 310) | -2 (-79 to 370) | -2 (-79 to 384) | -1 (-79 to 369) | 6.28 (2.07 to 13.17) | 3.71 (1.47 to 7.13) | 3.63 (1.46 to 7.06) | 3.59 (1.47 to 6.82) | 1,824.62 (602.54 to 3,824.23) | 1,284.96 (507.90 to 2,470.09) | 1,264.43 (509.43 to 2,459.37) | 1,257.45 (515.23 to 2,386.94) | -31 (-87 to 296) | -30 (-87 to 310) | -2 (-79 to 370) | -2 (-79 to 384) | -1 (-79 to 369) |
| **Niue** | 0.01 (0.00 to 0.02) | 0.00 (0.00 to 0.01) | 0.01 (0.01 to 0.02) | 0.01 (0.01 to 0.02) | 20.98 (8.14 to 39.05) | 14.07 (6.81 to 24.52) | 46.11 (24.31 to 74.97) | 46.78 (25.28 to 74.79) | 123 (-35 to 819) | -33 (-83 to 201) | 233 (3 to 998) | 228 (-1 to 1,001) | 1 (-66 to 208) | 0.87 (0.34 to 1.62) | 0.29 (0.14 to 0.50) | 0.94 (0.50 to 1.53) | 0.95 (0.51 to 1.52) | 1,882.11 (730.39 to 3,503.11) | 1,262.11 (611.09 to 2,200.09) | 4,137.02 (2,181.55 to 6,727.58) | 4,197.32 (2,268.67 to 6,712.32) | 123 (-35 to 819) | -33 (-83 to 201) | 233 (3 to 998) | 228 (-1 to 1,001) | 1 (-66 to 208) |
| **Northern Mariana Islands** | 0.11 (0.04 to 0.22) | 0.04 (0.02 to 0.07) | 0.03 (0.02 to 0.06) | 0.03 (0.02 to 0.06) | 9.92 (3.66 to 19.03) | 5.63 (2.80 to 10.48) | 5.46 (2.60 to 10.46) | 5.22 (2.54 to 9.60) | -47 (-87 to 162) | -43 (-85 to 186) | -7 (-76 to 243) | -3 (-75 to 273) | -4 (-76 to 270) | 10.21 (3.76 to 19.59) | 3.22 (1.60 to 6.00) | 3.02 (1.44 to 5.79) | 2.78 (1.35 to 5.10) | 890.39 (328.30 to 1,707.86) | 505.26 (251.45 to 940.57) | 490.02 (233.02 to 938.42) | 468.65 (227.54 to 861.45) | -47 (-87 to 162) | -43 (-85 to 186) | -7 (-76 to 243) | -3 (-75 to 273) | -4 (-76 to 270) |
| **Palau** | 0.07 (0.02 to 0.16) | 0.03 (0.01 to 0.04) | 0.02 (0.01 to 0.04) | 0.02 (0.01 to 0.04) | 22.62 (7.10 to 54.74) | 12.88 (6.20 to 22.90) | 12.64 (6.10 to 22.66) | 12.49 (6.00 to 22.11) | -45 (-89 to 212) | -43 (-89 to 223) | -3 (-74 to 257) | -2 (-73 to 266) | -1 (-74 to 263) | 6.12 (1.92 to 14.80) | 2.24 (1.08 to 3.99) | 2.11 (1.02 to 3.79) | 2.01 (0.97 to 3.56) | 2,029.46 (636.85 to 4,909.16) | 1,155.68 (555.92 to 2,054.95) | 1,134.46 (547.16 to 2,033.38) | 1,120.52 (538.28 to 1,984.14) | -45 (-89 to 212) | -43 (-89 to 223) | -3 (-74 to 257) | -2 (-73 to 266) | -1 (-74 to 263) |
| **Independent State of Papua New Guinea** | 91.43 (31.50 to 176.73) | 126.72 (58.17 to 213.82) | 126.28 (60.14 to 213.18) | 125.62 (58.65 to 219.37) | 65.46 (22.56 to 126.54) | 40.51 (18.60 to 68.36) | 39.42 (18.77 to 66.54) | 38.33 (17.90 to 66.94) | -41 (-86 to 197) | -38 (-85 to 203) | -5 (-74 to 260) | -3 (-73 to 258) | -3 (-73 to 257) | 8,203.07 (2,827.13 to 15,854.19) | 11,368.86 (5,220.66 to 19,181.93) | 11,329.29 (5,395.53 to 19,121.20) | 11,270.49 (5,263.97 to 19,672.25) | 5,873.43 (2,024.24 to 11,351.67) | 3,634.72 (1,669.09 to 6,132.63) | 3,536.22 (1,684.11 to 5,968.31) | 3,439.08 (1,606.25 to 6,002.80) | -41 (-86 to 197) | -38 (-85 to 203) | -5 (-74 to 260) | -3 (-73 to 258) | -3 (-73 to 257) |
| **Samoa** | 1.53 (0.61 to 2.76) | 0.81 (0.34 to 1.71) | 0.79 (0.32 to 1.70) | 0.78 (0.32 to 1.67) | 28.87 (11.47 to 51.90) | 13.56 (5.73 to 28.62) | 13.18 (5.37 to 28.33) | 12.77 (5.27 to 27.52) | -56 (-90 to 140) | -53 (-89 to 150) | -6 (-82 to 380) | -3 (-81 to 394) | -3 (-81 to 412) | 137.63 (54.66 to 247.43) | 72.58 (30.68 to 153.16) | 71.03 (28.94 to 152.62) | 69.56 (28.69 to 149.89) | 2,590.84 (1,028.87 to 4,657.80) | 1,216.83 (514.45 to 2,567.98) | 1,182.90 (482.03 to 2,541.86) | 1,145.88 (472.67 to 2,469.38) | -56 (-90 to 140) | -53 (-89 to 150) | -6 (-82 to 380) | -3 (-81 to 394) | -3 (-81 to 412) |
| **Solomon Islands** | 4.22 (1.49 to 8.14) | 2.89 (1.40 to 5.29) | 2.86 (1.36 to 5.23) | 2.85 (1.32 to 5.35) | 32.27 (11.41 to 62.27) | 14.80 (7.17 to 27.06) | 14.50 (6.91 to 26.53) | 14.30 (6.63 to 26.83) | -56 (-89 to 135) | -54 (-88 to 137) | -3 (-75 to 274) | -2 (-74 to 270) | -1 (-75 to 288) | 378.57 (133.83 to 730.39) | 259.70 (125.76 to 474.87) | 256.67 (122.27 to 469.50) | 255.61 (118.58 to 479.83) | 2,895.44 (1,023.62 to 5,586.35) | 1,328.05 (643.10 to 2,428.38) | 1,301.03 (619.80 to 2,379.87) | 1,282.79 (595.10 to 2,408.05) | -56 (-89 to 135) | -54 (-88 to 137) | -3 (-75 to 274) | -2 (-74 to 270) | -1 (-75 to 288) |
| **Tokelau** | 0.01 (0.00 to 0.02) | 0.00 (0.00 to 0.00) | 0.02 (0.01 to 0.03) | 0.02 (0.01 to 0.03) | 23.03 (9.72 to 42.05) | 10.54 (4.20 to 21.28) | 86.97 (39.13 to 160.63) | 87.78 (38.64 to 160.42) | 281 (-8 to 1,550) | -54 (-90 to 119) | 733 (82 to 3,717) | 725 (84 to 3,721) | 1 (-76 to 310) | 0.77 (0.32 to 1.40) | 0.17 (0.07 to 0.34) | 1.38 (0.62 to 2.55) | 1.38 (0.61 to 2.51) | 2,066.70 (872.17 to 3,773.61) | 945.50 (377.13 to 1,909.91) | 7,801.72 (3,509.28 to 14,411.22) | 7,874.84 (3,465.23 to 14,391.48) | 281 (-8 to 1,550) | -54 (-90 to 119) | 733 (82 to 3,717) | 725 (84 to 3,721) | 1 (-76 to 310) |
| **Tonga** | 0.61 (0.23 to 1.10) | 0.29 (0.14 to 0.59) | 0.28 (0.13 to 0.58) | 0.28 (0.12 to 0.56) | 19.20 (7.11 to 34.43) | 9.71 (4.58 to 19.60) | 9.53 (4.35 to 19.65) | 9.42 (4.18 to 18.87) | -51 (-88 to 166) | -49 (-87 to 176) | -3 (-79 to 312) | -2 (-78 to 329) | -1 (-79 to 334) | 55.10 (20.39 to 98.84) | 26.15 (12.35 to 52.80) | 25.41 (11.60 to 52.38) | 24.90 (11.04 to 49.87) | 1,722.61 (637.62 to 3,090.03) | 871.22 (411.33 to 1,759.31) | 855.56 (390.54 to 1,763.51) | 845.25 (374.96 to 1,693.08) | -51 (-88 to 166) | -49 (-87 to 176) | -3 (-79 to 312) | -2 (-78 to 329) | -1 (-79 to 334) |
| **Tuvalu** | 0.12 (0.02 to 0.33) | 0.03 (0.01 to 0.06) | 0.03 (0.01 to 0.06) | 0.03 (0.01 to 0.06) | 35.41 (7.11 to 98.18) | 12.87 (5.41 to 23.31) | 12.48 (5.61 to 22.76) | 12.18 (5.66 to 22.88) | -66 (-94 to 222) | -64 (-94 to 228) | -5 (-76 to 323) | -3 (-76 to 321) | -2 (-75 to 308) | 10.65 (2.14 to 29.53) | 2.95 (1.24 to 5.35) | 2.89 (1.30 to 5.28) | 2.85 (1.32 to 5.35) | 3,177.06 (638.02 to 8,808.36) | 1,155.26 (485.05 to 2,091.73) | 1,119.99 (503.26 to 2,043.19) | 1,093.29 (507.86 to 2,052.45) | -66 (-94 to 222) | -64 (-94 to 228) | -5 (-76 to 323) | -3 (-76 to 321) | -2 (-75 to 308) |
| **Vanuatu** | 1.48 (0.58 to 2.80) | 1.15 (0.55 to 2.11) | 1.12 (0.52 to 2.12) | 1.11 (0.53 to 2.11) | 25.05 (9.90 to 47.62) | 13.56 (6.51 to 24.91) | 13.18 (6.12 to 24.92) | 12.99 (6.19 to 24.65) | -48 (-87 to 149) | -46 (-86 to 152) | -4 (-75 to 278) | -3 (-75 to 283) | -1 (-75 to 303) | 132.37 (52.33 to 251.74) | 103.22 (49.58 to 189.67) | 100.76 (46.80 to 190.58) | 99.91 (47.57 to 189.48) | 2,247.57 (888.62 to 4,274.56) | 1,216.53 (584.35 to 2,235.48) | 1,182.21 (549.13 to 2,236.17) | 1,166.09 (555.27 to 2,211.55) | -48 (-87 to 149) | -46 (-86 to 152) | -4 (-75 to 278) | -3 (-75 to 283) | -1 (-75 to 303) |
| **Central sub-Saharan Africa** | | | | | | | | | | | | | | | | | | | | | | | | | | |
| **Angola** | 369.77 (158.01 to 650.91) | 357.51 (162.30 to 642.12) | 331.82 (156.64 to 570.01) | 318.86 (144.21 to 567.24) | 80.68 (34.48 to 142.02) | 31.62 (14.35 to 56.79) | 29.01 (13.69 to 49.83) | 27.51 (12.44 to 48.94) | -66 (-91 to 42) | -61 (-90 to 65) | -13 (-78 to 241) | -8 (-76 to 247) | -5 (-75 to 257) | 33,179.61 (14,183.64 to 58,399.15) | 32,071.42 (14,563.25 to 57,582.38) | 29,766.66 (14,053.61 to 51,107.73) | 28,604.55 (12,939.17 to 50,883.36) | 7,239.47 (3,094.73 to 12,742.14) | 2,836.42 (1,287.99 to 5,092.64) | 2,602.17 (1,228.55 to 4,467.79) | 2,468.06 (1,116.42 to 4,390.32) | -66 (-91 to 42) | -61 (-90 to 65) | -13 (-78 to 241) | -8 (-76 to 247) | -5 (-75 to 257) |
| **Central African Republic** | 71.66 (21.33 to 176.72) | 71.79 (26.16 to 160.64) | 69.67 (25.04 to 154.68) | 67.41 (23.85 to 152.53) | 61.93 (18.44 to 152.75) | 40.35 (14.70 to 90.29) | 38.89 (13.98 to 86.34) | 37.66 (13.33 to 85.22) | -39 (-91 to 362) | -35 (-90 to 390) | -7 (-85 to 480) | -4 (-85 to 487) | -3 (-85 to 510) | 6,429.24 (1,914.76 to 15,852.35) | 6,440.61 (2,347.47 to 14,408.41) | 6,250.71 (2,247.17 to 13,873.51) | 6,047.39 (2,140.12 to 13,683.51) | 5,557.05 (1,655.01 to 13,701.83) | 3,620.02 (1,319.42 to 8,098.41) | 3,489.15 (1,254.37 to 7,744.20) | 3,378.98 (1,195.79 to 7,645.68) | -39 (-91 to 362) | -35 (-90 to 390) | -7 (-85 to 480) | -4 (-85 to 487) | -3 (-85 to 510) |
| **Congo** | 28.36 (8.55 to 74.46) | 20.95 (7.58 to 49.03) | 19.59 (7.47 to 46.33) | 18.67 (7.19 to 42.35) | 32.90 (9.92 to 86.37) | 16.20 (5.86 to 37.91) | 15.49 (5.90 to 36.63) | 14.97 (5.77 to 33.96) | -54 (-93 to 242) | -51 (-93 to 282) | -8 (-85 to 479) | -4 (-84 to 525) | -3 (-84 to 475) | 2,544.59 (767.43 to 6,678.61) | 1,879.82 (680.00 to 4,396.37) | 1,757.60 (670.11 to 4,155.72) | 1,674.93 (645.43 to 3,798.26) | 2,951.59 (890.18 to 7,746.85) | 1,453.72 (525.87 to 3,399.85) | 1,389.56 (529.79 to 3,285.52) | 1,343.32 (517.65 to 3,046.28) | -54 (-93 to 242) | -51 (-93 to 282) | -8 (-85 to 479) | -4 (-84 to 525) | -3 (-84 to 475) |
| **Democratic Republic of the Congo** | 687.34 (174.72 to 1,750.10) | 511.71 (185.62 to 1,213.45) | 483.64 (177.39 to 1,090.38) | 451.34 (168.02 to 969.92) | 41.23 (10.48 to 104.98) | 18.40 (6.68 to 43.64) | 17.49 (6.42 to 39.43) | 16.39 (6.10 to 35.22) | -60 (-94 to 236) | -55 (-94 to 316) | -11 (-86 to 428) | -5 (-85 to 490) | -6 (-85 to 449) | 61,671.34 (15,684.86 to 156,985.56) | 45,909.46 (16,659.34 to 108,785.22) | 43,390.71 (15,921.80 to 97,788.34) | 40,493.86 (15,073.91 to 86,960.59) | 3,699.46 (940.88 to 9,417.04) | 1,650.90 (599.07 to 3,911.91) | 1,569.19 (575.80 to 3,536.43) | 1,470.37 (547.35 to 3,157.62) | -60 (-94 to 236) | -55 (-94 to 316) | -11 (-86 to 428) | -5 (-85 to 490) | -6 (-85 to 449) |
| **Republic of Equatorial Guinea** | 17.39 (8.12 to 31.80) | 8.08 (3.16 to 18.04) | 7.48 (2.87 to 16.23) | 7.25 (2.70 to 15.58) | 91.91 (42.91 to 168.13) | 21.71 (8.49 to 48.48) | 20.37 (7.80 to 44.19) | 19.91 (7.41 to 42.75) | -78 (-96 to 0) | -76 (-95 to 13) | -8 (-85 to 403) | -6 (-84 to 420) | -2 (-83 to 448) | 1,559.96 (728.09 to 2,854.78) | 724.55 (283.54 to 1,617.11) | 671.18 (257.17 to 1,454.65) | 650.85 (242.30 to 1,396.66) | 8,246.93 (3,849.11 to 15,092.13) | 1,947.51 (762.13 to 4,346.63) | 1,827.23 (700.14 to 3,960.20) | 1,786.20 (664.97 to 3,832.97) | -78 (-96 to 0) | -76 (-95 to 13) | -8 (-85 to 403) | -6 (-84 to 420) | -2 (-83 to 448) |
| **Gabonese Republic** | 8.75 (3.29 to 19.71) | 6.76 (2.73 to 13.85) | 5.99 (2.43 to 12.19) | 5.47 (2.24 to 10.83) | 25.83 (9.73 to 58.22) | 15.61 (6.31 to 31.99) | 14.04 (5.70 to 28.59) | 12.96 (5.31 to 25.68) | -50 (-91 to 164) | -40 (-89 to 229) | -17 (-83 to 307) | -10 (-82 to 353) | -8 (-81 to 350) | 784.75 (295.70 to 1,767.53) | 606.13 (244.98 to 1,242.31) | 537.24 (218.11 to 1,093.22) | 490.74 (201.24 to 971.81) | 2,317.51 (873.25 to 5,219.84) | 1,400.13 (565.89 to 2,869.66) | 1,260.08 (511.58 to 2,564.11) | 1,163.22 (477.00 to 2,303.51) | -50 (-91 to 164) | -40 (-89 to 229) | -17 (-83 to 307) | -10 (-82 to 353) | -8 (-81 to 350) |
| **Eastern sub-Saharan Africa** | | | | | | | | | | | | | | | | | | | | | | | | | | |
| **Republic of Burundi** | 183.68 (63.79 to 371.71) | 86.29 (29.84 to 193.31) | 83.91 (30.37 to 182.30) | 83.29 (30.38 to 172.28) | 76.00 (26.39 to 153.80) | 19.75 (6.83 to 44.24) | 18.89 (6.84 to 41.05) | 18.55 (6.77 to 38.37) | -76 (-96 to 45) | -74 (-96 to 68) | -6 (-85 to 462) | -4 (-85 to 501) | -2 (-84 to 461) | 16,482.25 (5,724.01 to 33,358.30) | 7,742.04 (2,678.22 to 17,342.99) | 7,529.06 (2,725.74 to 16,355.23) | 7,472.78 (2,725.88 to 15,453.21) | 6,819.53 (2,368.31 to 13,802.00) | 1,771.80 (612.92 to 3,969.02) | 1,695.26 (613.73 to 3,682.57) | 1,664.33 (607.11 to 3,441.72) | -76 (-96 to 45) | -74 (-96 to 68) | -6 (-85 to 462) | -4 (-85 to 501) | -2 (-84 to 461) |
| **Union of the Comoros** | 13.93 (5.70 to 29.15) | 4.41 (1.56 to 10.02) | 4.27 (1.62 to 9.71) | 4.15 (1.50 to 9.51) | 75.55 (30.93 to 158.12) | 26.39 (9.34 to 59.95) | 25.78 (9.77 to 58.57) | 25.18 (9.11 to 57.63) | -67 (-94 to 86) | -65 (-94 to 94) | -5 (-85 to 517) | -2 (-84 to 527) | -2 (-84 to 490) | 1,249.90 (511.91 to 2,614.77) | 395.46 (140.01 to 898.14) | 383.25 (145.29 to 870.28) | 372.58 (134.86 to 852.41) | 6,778.95 (2,776.41 to 14,181.48) | 2,367.17 (838.06 to 5,376.14) | 2,312.65 (876.70 to 5,251.58) | 2,258.38 (817.44 to 5,166.84) | -67 (-94 to 86) | -65 (-94 to 94) | -5 (-85 to 517) | -2 (-84 to 527) | -2 (-84 to 490) |
| **Republic of Djibouti** | 4.58 (1.11 to 12.94) | 5.47 (1.69 to 13.86) | 5.25 (1.63 to 12.98) | 4.97 (1.60 to 12.07) | 31.48 (7.64 to 88.97) | 18.42 (5.70 to 46.66) | 17.68 (5.51 to 43.76) | 16.87 (5.43 to 40.93) | -46 (-94 to 436) | -41 (-94 to 511) | -8 (-88 to 618) | -4 (-88 to 667) | -5 (-88 to 644) | 410.91 (99.69 to 1,161.04) | 490.83 (152.05 to 1,242.70) | 470.56 (146.55 to 1,164.08) | 446.03 (143.69 to 1,082.24) | 2,824.66 (685.30 to 7,981.12) | 1,652.49 (511.91 to 4,183.81) | 1,586.34 (494.04 to 3,924.28) | 1,512.96 (487.40 to 3,671.00) | -46 (-94 to 436) | -41 (-94 to 511) | -8 (-88 to 618) | -4 (-88 to 667) | -5 (-88 to 644) |
| **Eritrea** | 121.19 (48.43 to 296.27) | 83.02 (37.75 to 158.34) | 80.44 (36.09 to 156.03) | 78.04 (34.33 to 147.69) | 87.22 (34.85 to 213.23) | 44.47 (20.22 to 84.82) | 42.82 (19.21 to 83.07) | 41.28 (18.15 to 78.11) | -53 (-91 to 124) | -49 (-91 to 143) | -7 (-79 to 286) | -4 (-77 to 311) | -4 (-78 to 307) | 10,874.40 (4,345.62 to 26,591.60) | 7,447.60 (3,384.88 to 14,211.13) | 7,215.63 (3,235.64 to 14,002.09) | 7,000.61 (3,078.71 to 13,252.10) | 7,826.63 (3,127.67 to 19,138.79) | 3,989.48 (1,813.19 to 7,612.53) | 3,841.50 (1,722.61 to 7,454.51) | 3,702.57 (1,628.31 to 7,008.93) | -53 (-91 to 124) | -49 (-91 to 143) | -7 (-79 to 286) | -4 (-77 to 311) | -4 (-78 to 307) |
| **Ethiopia** | 3,557.72 (1,410.23 to 7,897.38) | 1,490.06 (727.57 to 2,599.93) | 1,318.55 (641.41 to 2,341.48) | 1,300.04 (627.77 to 2,297.57) | 160.79 (63.73 to 356.91) | 45.61 (22.27 to 79.58) | 39.92 (19.42 to 70.88) | 38.84 (18.76 to 68.65) | -76 (-95 to 8) | -72 (-94 to 25) | -15 (-76 to 208) | -12 (-76 to 218) | -3 (-74 to 254) | 319,247.84 (126,504.99 to 708,720.19) | 133,693.31 (65,270.06 to 233,314.06) | 118,293.48 (57,545.32 to 210,129.67) | 116,634.41 (56,320.99 to 206,189.41) | 14,427.92 (5,717.20 to 32,029.53) | 4,092.04 (1,997.77 to 7,141.21) | 3,581.05 (1,742.05 to 6,361.18) | 3,484.86 (1,682.79 to 6,160.63) | -76 (-95 to 8) | -72 (-94 to 25) | -15 (-76 to 208) | -12 (-76 to 218) | -3 (-74 to 254) |
| **Keny** | 468.52 (243.34 to 755.04) | 283.02 (149.27 to 468.66) | 240.77 (132.18 to 396.37) | 225.83 (126.15 to 376.19) | 50.27 (26.11 to 81.02) | 23.52 (12.41 to 38.95) | 20.42 (11.21 to 33.61) | 19.50 (10.89 to 32.48) | -61 (-87 to 24) | -53 (-85 to 49) | -17 (-72 to 162) | -13 (-71 to 171) | -5 (-68 to 190) | 42,051.27 (21,834.48 to 67,776.05) | 25,399.54 (13,397.89 to 42,062.55) | 21,602.51 (11,861.08 to 35,569.07) | 20,263.23 (11,319.48 to 33,753.23) | 4,512.11 (2,342.84 to 7,272.39) | 2,111.10 (1,113.58 to 3,496.06) | 1,831.89 (1,005.82 to 3,016.25) | 1,749.39 (977.25 to 2,914.03) | -61 (-87 to 24) | -53 (-85 to 49) | -17 (-72 to 162) | -13 (-71 to 171) | -5 (-68 to 190) |
| **Madagascar** | 284.66 (111.09 to 654.66) | 348.31 (161.30 to 597.12) | 326.81 (148.62 to 572.19) | 302.90 (136.79 to 534.97) | 58.75 (22.93 to 135.12) | 41.68 (19.30 to 71.46) | 39.51 (17.97 to 69.18) | 36.97 (16.70 to 65.29) | -37 (-88 to 185) | -29 (-86 to 212) | -11 (-77 to 238) | -5 (-75 to 258) | -6 (-76 to 263) | 25,540.49 (9,970.11 to 58,717.68) | 31,249.40 (14,474.00 to 53,560.26) | 29,319.37 (13,337.43 to 51,306.20) | 27,174.13 (12,274.78 to 47,981.52) | 5,271.60 (2,057.85 to 12,119.42) | 3,739.74 (1,732.16 to 6,409.77) | 3,544.70 (1,612.49 to 6,202.89) | 3,316.55 (1,498.11 to 5,856.05) | -37 (-88 to 185) | -29 (-86 to 212) | -11 (-77 to 238) | -5 (-75 to 258) | -6 (-76 to 263) |
| **Malawi** | 306.23 (91.59 to 799.33) | 117.88 (40.23 to 286.48) | 112.42 (38.70 to 264.29) | 108.72 (38.59 to 252.28) | 67.04 (20.05 to 174.98) | 21.19 (7.23 to 51.49) | 20.31 (6.99 to 47.74) | 19.68 (6.99 to 45.67) | -71 (-96 to 128) | -68 (-96 to 157) | -7 (-86 to 532) | -4 (-86 to 560) | -3 (-85 to 553) | 27,479.01 (8,220.06 to 71,726.51) | 10,575.36 (3,609.40 to 25,696.78) | 10,085.54 (3,472.83 to 23,708.41) | 9,753.87 (3,462.98 to 22,629.79) | 6,015.42 (1,799.45 to 15,701.62) | 1,900.55 (648.66 to 4,618.09) | 1,821.84 (627.33 to 4,282.66) | 1,765.54 (626.83 to 4,096.20) | -71 (-96 to 128) | -68 (-96 to 157) | -7 (-86 to 532) | -4 (-86 to 560) | -3 (-85 to 553) |
| **Mozambique** | 663.75 (251.79 to 1,240.75) | 430.09 (161.91 to 920.72) | 404.76 (153.66 to 862.57) | 382.02 (142.94 to 827.89) | 119.48 (45.32 to 223.34) | 41.30 (15.55 to 88.41) | 38.55 (14.64 to 82.16) | 36.04 (13.48 to 78.10) | -70 (-94 to 72) | -65 (-93 to 95) | -13 (-85 to 402) | -7 (-83 to 428) | -7 (-84 to 434) | 59,554.41 (22,596.43 to 111,330.34) | 38,581.00 (14,527.59 to 82,588.19) | 36,308.28 (13,782.07 to 77,371.60) | 34,269.18 (12,825.43 to 74,230.29) | 10,720.19 (4,067.51 to 20,040.19) | 3,704.71 (1,395.00 to 7,930.47) | 3,458.48 (1,312.79 to 7,369.89) | 3,232.95 (1,209.95 to 7,002.87) | -70 (-94 to 72) | -65 (-93 to 95) | -13 (-85 to 402) | -7 (-83 to 428) | -7 (-84 to 434) |
| **Rwanda** | 267.47 (118.11 to 479.97) | 67.82 (24.96 to 144.98) | 67.71 (25.97 to 144.39) | 65.73 (24.65 to 138.63) | 90.41 (39.92 to 162.24) | 19.15 (7.05 to 40.93) | 19.04 (7.30 to 40.61) | 18.34 (6.88 to 38.69) | -80 (-96 to -3) | -79 (-96 to 3) | -4 (-83 to 449) | -1 (-82 to 476) | -4 (-83 to 430) | 23,997.42 (10,595.19 to 43,070.61) | 6,084.38 (2,239.58 to 13,005.73) | 6,074.71 (2,330.86 to 12,951.14) | 5,896.57 (2,211.72 to 12,435.13) | 8,111.64 (3,581.40 to 14,558.79) | 1,717.59 (632.22 to 3,671.45) | 1,708.49 (655.55 to 3,642.46) | 1,645.67 (617.27 to 3,470.52) | -80 (-96 to -3) | -79 (-96 to 3) | -4 (-83 to 449) | -1 (-82 to 476) | -4 (-83 to 430) |
| **Federal Republic of Somalia** | 325.20 (153.57 to 599.61) | 510.05 (253.15 to 907.60) | 509.65 (257.60 to 903.67) | 510.91 (251.50 to 891.41) | 92.08 (43.48 to 169.79) | 60.29 (29.92 to 107.28) | 58.29 (29.46 to 103.35) | 56.54 (27.83 to 98.65) | -39 (-84 to 127) | -35 (-82 to 147) | -6 (-74 to 230) | -3 (-73 to 245) | -3 (-73 to 235) | 29,183.65 (13,779.66 to 53,794.92) | 45,768.44 (22,718.66 to 81,439.34) | 45,732.62 (23,117.33 to 81,092.71) | 45,848.36 (22,568.13 to 79,989.78) | 8,263.71 (3,901.88 to 15,232.70) | 5,409.89 (2,685.38 to 9,626.24) | 5,230.48 (2,643.95 to 9,274.65) | 5,073.98 (2,497.58 to 8,852.36) | -39 (-84 to 127) | -35 (-82 to 147) | -6 (-74 to 230) | -3 (-73 to 245) | -3 (-73 to 235) |
| **South Sudan** | 298.17 (123.42 to 543.28) | 349.43 (156.96 to 653.40) | 366.42 (167.00 to 681.13) | 384.34 (178.48 to 708.41) | 127.70 (52.86 to 232.68) | 107.23 (48.17 to 200.50) | 106.08 (48.35 to 197.19) | 108.00 (50.15 to 199.07) | -15 (-78 to 277) | -16 (-79 to 279) | 1 (-75 to 313) | -1 (-76 to 310) | 2 (-75 to 312) | 26,753.81 (11,076.97 to 48,747.95) | 31,349.40 (14,082.49 to 58,640.27) | 32,874.43 (14,984.17 to 61,133.44) | 34,481.99 (16,012.70 to 63,557.46) | 11,458.45 (4,744.18 to 20,878.37) | 9,620.08 (4,321.44 to 17,994.73) | 9,517.15 (4,337.92 to 17,698.14) | 9,689.88 (4,499.77 to 17,860.46) | -15 (-78 to 277) | -16 (-79 to 279) | 1 (-75 to 313) | -1 (-76 to 310) | 2 (-75 to 312) |
| **Uganda** | 525.03 (214.03 to 974.68) | 369.63 (141.83 to 810.50) | 365.01 (143.40 to 770.68) | 360.43 (143.57 to 740.99) | 61.76 (25.18 to 114.65) | 24.34 (9.34 to 53.37) | 24.01 (9.43 to 50.70) | 23.65 (9.42 to 48.62) | -62 (-92 to 93) | -61 (-92 to 112) | -3 (-82 to 421) | -1 (-82 to 443) | -2 (-81 to 415) | 47,111.89 (19,207.90 to 87,443.16) | 33,160.29 (12,725.97 to 72,697.10) | 32,743.92 (12,866.88 to 69,127.79) | 32,334.50 (12,881.79 to 66,459.56) | 5,541.63 (2,259.37 to 10,285.68) | 2,183.48 (837.96 to 4,786.84) | 2,153.95 (846.40 to 4,547.34) | 2,121.61 (845.23 to 4,360.71) | -62 (-92 to 93) | -61 (-92 to 112) | -3 (-82 to 421) | -1 (-82 to 443) | -2 (-81 to 415) |
| **United Republic of Tanzania** | 788.94 (242.30 to 1,879.82) | 772.82 (313.10 to 1,530.75) | 716.55 (286.03 to 1,461.34) | 662.44 (255.55 to 1,311.47) | 71.20 (21.87 to 169.65) | 42.34 (17.16 to 83.87) | 39.28 (15.68 to 80.12) | 36.31 (14.01 to 71.89) | -49 (-92 to 229) | -41 (-90 to 284) | -14 (-83 to 319) | -7 (-81 to 367) | -8 (-83 to 358) | 70,784.53 (21,741.26 to 168,673.37) | 69,328.66 (28,083.39 to 137,375.80) | 64,282.32 (25,649.34 to 131,061.79) | 59,426.95 (22,922.28 to 117,703.22) | 6,388.00 (1,962.05 to 15,222.04) | 3,798.62 (1,538.73 to 7,527.03) | 3,524.27 (1,406.22 to 7,185.45) | 3,257.70 (1,256.57 to 6,452.33) | -49 (-92 to 229) | -41 (-90 to 284) | -14 (-83 to 319) | -7 (-81 to 367) | -8 (-83 to 358) |
| **Zambia** | 223.71 (74.39 to 512.42) | 141.05 (50.86 to 329.42) | 135.26 (48.41 to 302.05) | 126.35 (45.00 to 277.41) | 63.03 (20.96 to 144.37) | 23.53 (8.48 to 54.96) | 22.79 (8.16 to 50.88) | 21.47 (7.65 to 47.14) | -66 (-95 to 125) | -63 (-94 to 162) | -9 (-86 to 456) | -3 (-85 to 500) | -6 (-85 to 478) | 20,069.53 (6,675.04 to 45,967.79) | 12,652.58 (4,562.89 to 29,537.87) | 12,133.44 (4,342.39 to 27,092.99) | 11,334.24 (4,038.58 to 24,878.81) | 5,654.43 (1,880.64 to 12,951.07) | 2,110.76 (761.20 to 4,927.65) | 2,043.89 (731.48 to 4,563.85) | 1,925.95 (686.25 to 4,227.48) | -66 (-95 to 125) | -63 (-94 to 162) | -9 (-86 to 456) | -3 (-85 to 500) | -6 (-85 to 478) |
| **Southern sub-Saharan Africa** | | | | | | | | | | | | | | | | | | | | | | | | | | |
| **Botswana** | 7.07 (2.41 to 15.71) | 6.55 (2.07 to 15.97) | 6.51 (2.07 to 15.77) | 6.51 (2.05 to 16.04) | 15.87 (5.40 to 35.24) | 13.75 (4.36 to 33.54) | 13.72 (4.37 to 33.23) | 13.77 (4.33 to 33.90) | -13 (-88 to 527) | -13 (-88 to 521) | 0 (-87 to 678) | 0 (-87 to 663) | 0 (-87 to 676) | 634.83 (216.19 to 1,409.17) | 587.32 (186.09 to 1,432.46) | 584.33 (186.14 to 1,414.93) | 584.24 (183.87 to 1,438.69) | 1,424.34 (485.06 to 3,161.70) | 1,233.56 (390.86 to 3,008.64) | 1,231.04 (392.16 to 2,980.94) | 1,235.08 (388.70 to 3,041.37) | -13 (-88 to 527) | -13 (-88 to 521) | 0 (-87 to 678) | 0 (-87 to 663) | 0 (-87 to 676) |
| **Eswatini** | 9.95 (3.14 to 21.27) | 6.71 (1.74 to 18.25) | 6.57 (1.73 to 18.06) | 6.35 (1.63 to 17.72) | 32.30 (10.21 to 69.04) | 22.99 (5.98 to 62.52) | 22.86 (6.03 to 62.87) | 22.45 (5.77 to 62.66) | -31 (-92 to 514) | -29 (-91 to 512) | -2 (-91 to 948) | -1 (-90 to 952) | -2 (-91 to 939) | 892.61 (282.15 to 1,907.85) | 602.11 (156.58 to 1,636.94) | 588.99 (155.43 to 1,619.85) | 569.46 (146.35 to 1,589.47) | 2,898.11 (916.09 to 6,194.36) | 2,062.36 (536.32 to 5,606.89) | 2,050.71 (541.18 to 5,639.93) | 2,013.89 (517.57 to 5,621.13) | -31 (-92 to 514) | -29 (-91 to 512) | -2 (-91 to 948) | -1 (-90 to 952) | -2 (-91 to 939) |
| **Lesotho** | 21.14 (9.89 to 39.14) | 8.04 (1.98 to 24.25) | 8.02 (2.01 to 24.37) | 8.09 (2.02 to 24.89) | 41.37 (19.35 to 76.59) | 19.36 (4.76 to 58.37) | 19.56 (4.89 to 59.41) | 19.94 (4.97 to 61.36) | -52 (-94 to 217) | -53 (-94 to 202) | 3 (-91 to 1,190) | 1 (-92 to 1,148) | 2 (-92 to 1,155) | 1,896.87 (887.10 to 3,512.97) | 721.69 (177.34 to 2,175.30) | 720.02 (180.01 to 2,185.92) | 725.91 (181.12 to 2,232.97) | 3,711.89 (1,735.91 to 6,874.35) | 1,737.23 (426.88 to 5,236.33) | 1,755.34 (438.84 to 5,329.06) | 1,789.49 (446.48 to 5,504.66) | -52 (-94 to 217) | -53 (-94 to 202) | 3 (-91 to 1,190) | 1 (-92 to 1,148) | 2 (-92 to 1,155) |
| **Namibia** | 10.83 (4.29 to 20.94) | 9.43 (3.11 to 20.73) | 9.17 (2.88 to 20.20) | 9.03 (2.89 to 19.64) | 22.19 (8.80 to 42.90) | 16.61 (5.47 to 36.50) | 16.25 (5.10 to 35.80) | 16.09 (5.15 to 34.99) | -27 (-88 to 298) | -25 (-87 to 315) | -3 (-86 to 539) | -2 (-86 to 554) | -1 (-86 to 587) | 971.85 (385.47 to 1,878.85) | 846.14 (278.75 to 1,859.40) | 822.65 (257.96 to 1,811.02) | 810.09 (259.48 to 1,761.33) | 1,990.93 (789.68 to 3,848.99) | 1,490.33 (490.98 to 3,275.03) | 1,458.12 (457.22 to 3,209.98) | 1,443.44 (462.36 to 3,138.41) | -27 (-88 to 298) | -25 (-87 to 315) | -3 (-86 to 539) | -2 (-86 to 554) | -1 (-86 to 587) |
| **South Africa** | 249.43 (81.60 to 610.85) | 183.34 (61.51 to 467.80) | 161.48 (55.30 to 418.66) | 157.63 (54.39 to 408.99) | 24.90 (8.15 to 60.98) | 18.17 (6.10 to 46.37) | 16.34 (5.59 to 42.35) | 16.30 (5.62 to 42.28) | -35 (-91 to 419) | -27 (-90 to 469) | -10 (-88 to 593) | -10 (-88 to 595) | 0 (-87 to 656) | 22,382.71 (7,323.24 to 54,806.29) | 16,450.25 (5,520.21 to 41,974.89) | 14,489.57 (4,962.76 to 37,564.57) | 14,144.16 (4,880.90 to 36,697.50) | 2,234.34 (731.04 to 5,471.01) | 1,630.46 (547.13 to 4,160.33) | 1,465.75 (502.03 to 3,800.00) | 1,462.14 (504.56 to 3,793.57) | -35 (-91 to 419) | -27 (-90 to 469) | -10 (-88 to 593) | -10 (-88 to 595) | 0 (-87 to 656) |
| **Zimbabwe** | 94.42 (38.69 to 202.95) | 96.69 (33.43 to 233.83) | 94.91 (33.92 to 228.42) | 99.34 (36.01 to 238.74) | 25.39 (10.40 to 54.57) | 21.30 (7.36 to 51.51) | 20.97 (7.49 to 50.47) | 21.99 (7.97 to 52.86) | -13 (-85 to 408) | -16 (-87 to 395) | 3 (-85 to 618) | -2 (-85 to 585) | 5 (-84 to 605) | 8,472.07 (3,472.17 to 18,203.74) | 8,675.53 (3,000.15 to 20,974.94) | 8,515.81 (3,044.20 to 20,493.13) | 8,913.27 (3,231.51 to 21,420.24) | 2,277.78 (933.52 to 4,894.22) | 1,911.30 (660.96 to 4,620.97) | 1,881.62 (672.63 to 4,528.08) | 1,973.37 (715.45 to 4,742.38) | -13 (-85 to 408) | -16 (-87 to 395) | 3 (-85 to 618) | -2 (-85 to 585) | 5 (-84 to 605) |
| **Western sub-Saharan Africa** | | | | | | | | | | | | | | | | | | | | | | | | | | |
| **Benin** | 108.42 (34.71 to 263.46) | 127.46 (38.49 to 310.16) | 127.22 (38.11 to 318.35) | 122.91 (37.15 to 314.89) | 49.34 (15.80 to 119.91) | 26.50 (8.00 to 64.49) | 25.97 (7.78 to 64.97) | 24.62 (7.44 to 63.08) | -50 (-94 to 299) | -46 (-93 to 308) | -7 (-88 to 688) | -2 (-88 to 711) | -5 (-89 to 711) | 9,725.27 (3,114.88 to 23,615.94) | 11,432.83 (3,454.24 to 27,812.81) | 11,410.81 (3,419.64 to 28,544.10) | 11,023.79 (3,333.31 to 28,232.59) | 4,426.21 (1,417.66 to 10,748.20) | 2,377.02 (718.18 to 5,782.61) | 2,328.88 (697.93 to 5,825.69) | 2,208.19 (667.70 to 5,655.30) | -50 (-94 to 299) | -46 (-93 to 308) | -7 (-88 to 688) | -2 (-88 to 711) | -5 (-89 to 711) |
| **Burkina Faso** | 317.09 (120.64 to 621.20) | 337.85 (111.60 to 878.07) | 334.71 (112.66 to 846.31) | 334.68 (115.50 to 838.67) | 74.43 (28.32 to 145.82) | 39.08 (12.91 to 101.57) | 37.96 (12.78 to 95.98) | 37.17 (12.83 to 93.16) | -50 (-91 to 229) | -47 (-91 to 259) | -5 (-87 to 622) | -3 (-87 to 643) | -2 (-87 to 629) | 28,452.66 (10,827.71 to 55,735.86) | 30,309.70 (10,015.67 to 78,756.91) | 30,028.47 (10,110.16 to 75,915.04) | 30,025.32 (10,364.44 to 75,225.47) | 6,679.03 (2,541.71 to 13,083.54) | 3,506.10 (1,158.57 to 9,110.27) | 3,405.47 (1,146.57 to 8,609.37) | 3,335.06 (1,151.23 to 8,355.65) | -50 (-91 to 229) | -47 (-91 to 259) | -5 (-87 to 622) | -3 (-87 to 643) | -2 (-87 to 629) |
| **Cabo Verde** | 7.31 (3.64 to 12.77) | 1.41 (0.78 to 2.57) | 1.27 (0.68 to 2.29) | 1.18 (0.62 to 2.11) | 60.20 (29.97 to 105.15) | 15.71 (8.69 to 28.69) | 14.58 (7.85 to 26.32) | 13.98 (7.39 to 24.96) | -77 (-93 to -17) | -74 (-92 to -4) | -11 (-74 to 187) | -7 (-73 to 203) | -4 (-72 to 218) | 655.76 (326.64 to 1,145.41) | 126.36 (69.90 to 230.80) | 113.77 (61.27 to 205.43) | 105.94 (55.93 to 189.15) | 5,400.53 (2,690.08 to 9,433.00) | 1,408.96 (779.46 to 2,573.54) | 1,307.93 (704.41 to 2,361.61) | 1,254.59 (662.41 to 2,239.97) | -77 (-93 to -17) | -74 (-92 to -4) | -11 (-74 to 187) | -7 (-73 to 203) | -4 (-72 to 218) |
| **Cameroon** | 183.63 (69.56 to 413.11) | 331.79 (130.50 to 697.44) | 308.08 (119.78 to 654.12) | 287.20 (112.57 to 604.91) | 40.99 (15.53 to 92.21) | 33.47 (13.16 to 70.35) | 30.97 (12.04 to 65.76) | 28.74 (11.27 to 60.54) | -30 (-88 to 290) | -18 (-86 to 353) | -14 (-84 to 360) | -7 (-83 to 399) | -7 (-83 to 403) | 16,474.04 (6,243.46 to 37,053.39) | 29,761.46 (11,709.30 to 62,549.00) | 27,635.26 (10,748.50 to 58,667.02) | 25,763.94 (10,101.25 to 54,240.24) | 3,677.36 (1,393.67 to 8,271.10) | 3,002.21 (1,181.18 to 6,309.68) | 2,778.27 (1,080.58 to 5,898.00) | 2,578.38 (1,010.91 to 5,428.21) | -30 (-88 to 290) | -18 (-86 to 353) | -14 (-84 to 360) | -7 (-83 to 399) | -7 (-83 to 403) |
| **Chad** | 263.70 (116.17 to 532.93) | 503.82 (234.99 to 915.21) | 508.01 (223.72 to 913.66) | 515.15 (231.19 to 930.43) | 90.08 (39.68 to 182.04) | 66.81 (31.16 to 121.36) | 65.12 (28.68 to 117.12) | 63.80 (28.63 to 115.22) | -29 (-84 to 190) | -26 (-83 to 206) | -5 (-76 to 270) | -3 (-76 to 276) | -2 (-76 to 302) | 23,658.60 (10,423.21 to 47,807.72) | 45,195.52 (21,086.25 to 82,129.87) | 45,571.90 (20,078.04 to 81,954.66) | 46,213.02 (20,745.69 to 83,493.67) | 8,081.62 (3,560.50 to 16,330.80) | 5,992.82 (2,795.99 to 10,890.24) | 5,841.53 (2,573.66 to 10,505.18) | 5,723.04 (2,569.16 to 10,339.90) | -29 (-84 to 190) | -26 (-83 to 206) | -5 (-76 to 270) | -3 (-76 to 276) | -2 (-76 to 302) |
| **Côte d'Ivoire** | 316.02 (129.48 to 599.85) | 260.69 (102.49 to 561.37) | 245.83 (97.42 to 529.88) | 235.41 (96.17 to 516.61) | 61.60 (25.24 to 116.92) | 28.99 (11.40 to 62.44) | 27.20 (10.78 to 58.62) | 25.90 (10.58 to 56.84) | -58 (-91 to 125) | -53 (-90 to 147) | -11 (-83 to 399) | -6 (-83 to 414) | -5 (-82 to 427) | 28,355.52 (11,620.47 to 53,825.72) | 23,385.94 (9,196.78 to 50,348.49) | 22,053.18 (8,742.05 to 47,530.64) | 21,118.40 (8,628.88 to 46,345.85) | 5,526.73 (2,264.93 to 10,491.08) | 2,601.03 (1,022.89 to 5,599.86) | 2,439.88 (967.19 to 5,258.61) | 2,323.55 (949.39 to 5,099.20) | -58 (-91 to 125) | -53 (-90 to 147) | -11 (-83 to 399) | -6 (-83 to 414) | -5 (-82 to 427) |
| **Gambia** | 17.76 (8.66 to 31.65) | 13.64 (6.73 to 24.14) | 12.88 (6.35 to 22.46) | 12.50 (6.37 to 21.31) | 42.02 (20.49 to 74.89) | 18.51 (9.13 to 32.74) | 17.36 (8.56 to 30.28) | 16.71 (8.52 to 28.49) | -60 (-89 to 39) | -56 (-88 to 60) | -10 (-74 to 212) | -6 (-74 to 232) | -4 (-72 to 233) | 1,593.24 (777.18 to 2,839.73) | 1,223.67 (603.86 to 2,164.57) | 1,154.88 (569.59 to 2,015.24) | 1,121.17 (571.98 to 1,911.80) | 3,769.25 (1,838.62 to 6,718.16) | 1,659.95 (819.16 to 2,936.30) | 1,557.02 (767.93 to 2,716.97) | 1,498.92 (764.70 to 2,555.94) | -60 (-89 to 39) | -56 (-88 to 60) | -10 (-74 to 212) | -6 (-74 to 232) | -4 (-72 to 233) |
| **Ghana** | 264.85 (125.40 to 457.20) | 192.52 (79.24 to 379.89) | 178.62 (74.21 to 349.78) | 167.92 (71.05 to 324.62) | 46.23 (21.89 to 79.80) | 20.50 (8.44 to 40.45) | 19.02 (7.90 to 37.24) | 17.85 (7.55 to 34.51) | -61 (-91 to 58) | -56 (-89 to 85) | -13 (-81 to 309) | -7 (-80 to 341) | -6 (-80 to 337) | 23,763.41 (11,253.80 to 41,031.52) | 17,272.08 (7,109.61 to 34,074.51) | 16,025.08 (6,658.26 to 31,382.19) | 15,065.42 (6,375.04 to 29,127.14) | 4,147.67 (1,964.24 to 7,161.65) | 1,839.01 (756.98 to 3,628.01) | 1,706.16 (708.89 to 3,341.20) | 1,601.57 (677.71 to 3,096.43) | -61 (-91 to 58) | -56 (-89 to 85) | -13 (-81 to 309) | -7 (-80 to 341) | -6 (-80 to 337) |
| **Guinea** | 287.84 (131.11 to 494.26) | 243.32 (104.32 to 470.35) | 234.61 (101.51 to 442.36) | 226.35 (98.21 to 425.98) | 108.16 (49.27 to 185.73) | 52.80 (22.64 to 102.06) | 50.30 (21.77 to 94.85) | 47.84 (20.76 to 90.04) | -56 (-89 to 83) | -51 (-88 to 107) | -9 (-80 to 298) | -5 (-79 to 319) | -5 (-78 to 314) | 25,828.88 (11,768.22 to 44,358.52) | 21,829.25 (9,360.84 to 42,190.79) | 21,048.45 (9,108.26 to 39,674.64) | 20,308.56 (8,812.55 to 38,211.88) | 9,705.74 (4,422.16 to 16,668.65) | 4,736.85 (2,031.26 to 9,155.21) | 4,513.11 (1,952.95 to 8,506.84) | 4,292.67 (1,862.73 to 8,076.94) | -56 (-89 to 83) | -51 (-88 to 107) | -9 (-80 to 298) | -5 (-79 to 319) | -5 (-78 to 314) |
| **Guinea-**  **Bissau** | 39.21 (17.60 to 68.51) | 30.59 (14.02 to 54.64) | 28.49 (13.01 to 51.09) | 26.74 (12.03 to 48.42) | 92.54 (41.55 to 161.68) | 44.53 (20.41 to 79.54) | 41.36 (18.89 to 74.17) | 38.70 (17.41 to 70.06) | -58 (-89 to 69) | -52 (-87 to 91) | -13 (-78 to 243) | -7 (-76 to 263) | -6 (-77 to 271) | 3,518.63 (1,579.86 to 6,148.70) | 2,745.07 (1,257.99 to 4,903.51) | 2,556.50 (1,167.75 to 4,584.40) | 2,399.73 (1,079.96 to 4,344.87) | 8,304.00 (3,728.47 to 14,510.99) | 3,995.93 (1,831.22 to 7,137.93) | 3,711.38 (1,695.28 to 6,655.36) | 3,472.24 (1,562.63 to 6,286.71) | -58 (-89 to 69) | -52 (-87 to 91) | -13 (-78 to 243) | -7 (-76 to 263) | -6 (-77 to 271) |
| **Liberia** | 107.38 (41.63 to 228.63) | 51.64 (21.62 to 105.83) | 49.31 (20.37 to 101.38) | 48.15 (20.32 to 97.58) | 104.83 (40.64 to 223.19) | 32.61 (13.65 to 66.82) | 31.32 (12.94 to 64.39) | 30.63 (12.93 to 62.07) | -71 (-94 to 53) | -69 (-94 to 64) | -6 (-81 to 355) | -4 (-81 to 371) | -2 (-80 to 380) | 9,633.48 (3,734.28 to 20,502.61) | 4,633.01 (1,940.17 to 9,489.46) | 4,423.75 (1,827.75 to 9,094.22) | 4,319.97 (1,822.91 to 8,752.48) | 9,404.48 (3,645.52 to 20,015.25) | 2,925.41 (1,225.08 to 5,991.92) | 2,809.53 (1,160.80 to 5,775.75) | 2,747.96 (1,159.56 to 5,567.51) | -71 (-94 to 53) | -69 (-94 to 64) | -6 (-81 to 355) | -4 (-81 to 371) | -2 (-80 to 380) |
| **Mali** | 313.05 (118.90 to 543.70) | 305.92 (104.03 to 711.75) | 302.53 (103.30 to 699.63) | 302.62 (103.96 to 681.94) | 76.60 (29.09 to 133.04) | 32.37 (11.01 to 75.32) | 31.11 (10.62 to 71.95) | 30.22 (10.38 to 68.11) | -61 (-92 to 134) | -58 (-92 to 159) | -7 (-86 to 519) | -4 (-86 to 553) | -3 (-86 to 541) | 28,088.22 (10,669.27 to 48,790.90) | 27,443.84 (9,336.26 to 63,826.91) | 27,139.10 (9,269.43 to 62,734.44) | 27,149.15 (9,329.34 to 61,158.53) | 6,872.88 (2,610.65 to 11,938.59) | 2,904.25 (988.01 to 6,754.49) | 2,790.81 (953.21 to 6,451.21) | 2,711.58 (931.79 to 6,108.33) | -61 (-92 to 134) | -58 (-92 to 159) | -7 (-86 to 519) | -4 (-86 to 553) | -3 (-86 to 541) |
| **Mauritania** | 34.44 (14.24 to 62.63) | 26.96 (12.14 to 48.16) | 25.00 (11.21 to 44.75) | 24.74 (11.05 to 46.16) | 41.99 (17.37 to 76.36) | 20.23 (9.11 to 36.14) | 18.85 (8.45 to 33.75) | 18.73 (8.36 to 34.94) | -55 (-89 to 101) | -52 (-88 to 108) | -7 (-77 to 284) | -7 (-77 to 270) | -1 (-75 to 313) | 3,089.54 (1,277.56 to 5,618.79) | 2,418.66 (1,089.38 to 4,319.45) | 2,242.42 (1,005.79 to 4,015.07) | 2,219.57 (991.28 to 4,139.64) | 3,767.11 (1,557.74 to 6,851.06) | 1,814.77 (817.39 to 3,240.99) | 1,690.91 (758.42 to 3,027.58) | 1,680.27 (750.42 to 3,133.81) | -55 (-89 to 101) | -52 (-88 to 108) | -7 (-77 to 284) | -7 (-77 to 270) | -1 (-75 to 313) |
| **Niger** | 468.34 (203.16 to 906.88) | 556.63 (240.58 to 1,115.35) | 567.20 (236.67 to 1,156.09) | 567.89 (231.93 to 1,107.94) | 115.17 (49.96 to 223.01) | 53.53 (23.14 to 107.26) | 52.60 (21.95 to 107.22) | 50.75 (20.73 to 99.02) | -56 (-91 to 98) | -54 (-90 to 115) | -5 (-81 to 328) | -2 (-80 to 363) | -4 (-81 to 351) | 42,018.08 (18,231.74 to 81,380.41) | 49,942.67 (21,578.16 to 100,085.91) | 50,888.33 (21,232.10 to 103,705.74) | 50,951.07 (20,800.74 to 99,424.55) | 10,332.88 (4,483.46 to 20,012.68) | 4,802.99 (2,075.17 to 9,625.26) | 4,719.49 (1,969.11 to 9,617.89) | 4,553.67 (1,859.03 to 8,885.91) | -56 (-91 to 98) | -54 (-90 to 115) | -5 (-81 to 328) | -2 (-80 to 363) | -4 (-81 to 351) |
| **Nigeria** | 2,845.39 (1,349.28 to 5,050.90) | 5,378.53 (2,530.72 to 9,162.03) | 4,921.45 (2,201.00 to 8,239.40) | 4,786.26 (2,075.20 to 8,117.05) | 75.44 (35.77 to 133.92) | 69.67 (32.78 to 118.69) | 63.26 (28.29 to 105.91) | 60.97 (26.44 to 103.40) | -19 (-80 to 189) | -8 (-76 to 232) | -12 (-78 to 215) | -9 (-76 to 223) | -4 (-75 to 265) | 255,315.35 (121,066.40 to 453,292.33) | 482,461.94 (227,047.19 to 821,902.80) | 441,441.93 (197,339.28 to 739,072.01) | 429,317.45 (186,071.91 to 728,042.61) | 6,769.36 (3,209.92 to 12,018.46) | 6,249.82 (2,941.17 to 10,646.95) | 5,674.39 (2,536.64 to 9,500.19) | 5,468.91 (2,370.30 to 9,274.26) | -19 (-80 to 189) | -8 (-76 to 232) | -12 (-78 to 215) | -9 (-76 to 223) | -4 (-75 to 265) |
| **Sao Tome and Principe** | 2.33 (1.05 to 4.61) | 0.81 (0.35 to 1.48) | 0.78 (0.33 to 1.42) | 0.71 (0.32 to 1.29) | 53.24 (24.09 to 105.28) | 16.05 (6.95 to 29.34) | 15.78 (6.74 to 28.70) | 14.62 (6.61 to 26.43) | -73 (-94 to 10) | -70 (-93 to 22) | -9 (-77 to 280) | -2 (-77 to 312) | -7 (-77 to 292) | 209.01 (94.59 to 413.12) | 72.80 (31.56 to 133.11) | 70.00 (29.89 to 127.24) | 63.92 (28.91 to 115.53) | 4,776.50 (2,161.65 to 9,440.99) | 1,439.79 (624.15 to 2,632.42) | 1,416.00 (604.71 to 2,574.04) | 1,312.16 (593.39 to 2,371.62) | -73 (-94 to 10) | -70 (-93 to 22) | -9 (-77 to 280) | -2 (-77 to 312) | -7 (-77 to 292) |
| **Senegal** | 181.11 (76.26 to 325.72) | 125.48 (48.48 to 268.15) | 115.79 (44.40 to 248.15) | 106.78 (40.70 to 231.42) | 55.48 (23.36 to 99.77) | 26.70 (10.32 to 57.06) | 24.72 (9.48 to 52.99) | 22.85 (8.71 to 49.52) | -59 (-91 to 112) | -52 (-90 to 144) | -14 (-85 to 380) | -7 (-83 to 413) | -8 (-84 to 422) | 16,250.40 (6,844.08 to 29,214.03) | 11,258.62 (4,350.76 to 24,060.71) | 10,389.76 (3,983.77 to 22,256.46) | 9,582.02 (3,652.06 to 20,764.85) | 4,977.70 (2,096.43 to 8,948.63) | 2,395.86 (925.85 to 5,120.16) | 2,218.54 (850.66 to 4,752.45) | 2,050.40 (781.48 to 4,443.36) | -59 (-91 to 112) | -52 (-90 to 144) | -14 (-85 to 380) | -7 (-83 to 413) | -8 (-84 to 422) |
| **Sierra Leone** | 183.23 (67.21 to 400.93) | 82.71 (20.50 to 232.52) | 79.61 (19.63 to 220.35) | 78.65 (20.11 to 219.74) | 99.40 (36.46 to 217.48) | 29.50 (7.31 to 82.93) | 28.13 (6.93 to 77.85) | 27.47 (7.03 to 76.75) | -72 (-97 to 111) | -70 (-97 to 127) | -7 (-92 to 950) | -5 (-92 to 964) | -2 (-91 to 1,007) | 16,437.77 (6,030.67 to 35,951.93) | 7,417.56 (1,839.77 to 20,846.29) | 7,139.95 (1,761.34 to 19,754.35) | 7,053.45 (1,804.91 to 19,703.31) | 8,916.70 (3,271.35 to 19,502.19) | 2,645.64 (656.20 to 7,435.30) | 2,522.55 (622.28 to 6,979.22) | 2,463.74 (630.45 to 6,882.28) | -72 (-97 to 111) | -70 (-97 to 127) | -7 (-92 to 950) | -5 (-92 to 964) | -2 (-91 to 1,007) |
| **Togolese Republic** | 63.89 (24.05 to 122.05) | 52.39 (19.85 to 105.67) | 48.29 (18.79 to 93.67) | 45.54 (18.31 to 88.50) | 42.47 (15.99 to 81.12) | 21.75 (8.24 to 43.88) | 20.27 (7.89 to 39.31) | 19.30 (7.76 to 37.50) | -55 (-90 to 135) | -49 (-90 to 174) | -11 (-82 to 355) | -7 (-82 to 377) | -5 (-80 to 376) | 5,731.88 (2,157.96 to 10,951.43) | 4,699.70 (1,780.95 to 9,473.70) | 4,332.14 (1,685.66 to 8,401.15) | 4,085.53 (1,642.67 to 7,935.43) | 3,809.72 (1,434.30 to 7,278.92) | 1,951.53 (739.53 to 3,933.93) | 1,818.30 (707.51 to 3,526.16) | 1,731.17 (696.05 to 3,362.50) | -55 (-90 to 135) | -49 (-90 to 174) | -11 (-82 to 355) | -7 (-82 to 377) | -5 (-80 to 376) |

*DALYs* Disability-Adjusted Life Years, *SIDS* sudden infant death syndrome, *UI* uncertainty interval.

**Supplementary Table 3. Sex-specific trends in SIDS deaths and DALYs from 1990 to 2021, with percentage change in rates**

| **Gender** | **Deaths (95% UI)** | | | | | | | | | | | | | **DALYs (95% UI)** | | | | | | | | | | | | | |
| --- | --- | --- | --- | --- | --- | --- | --- | --- | --- | --- | --- | --- | --- | --- | --- | --- | --- | --- | --- | --- | --- | --- | --- | --- | --- | --- | --- |
|  | **Number** | | | | **Rate (per 100,000)** | | | | **Percentage change in rates (%)** | | | | | **Number** | | | | **Rate (per 100,000)** | | | | **Percentage change in rates (%)** | | | | |  |
|  | **1990** | **2019** | **2020** | **2021** | **1990** | **2019** | **2020** | **2021** | **From 1990 to 2021** | **From 1990 to 2019** | **From 2019 to 2021** | **From 2019 to 2020** | **From 2020 to 2021** | **1990** | **2019** | **2020** | **2021** | **1990** | **2019** | **2020** | **2021** | **From 1990 to 2021** | **From 1990 to 2019** | **From 2019 to 2021** | **From 2019 to 2020** | **From 2020 to 2021** |  |
| **Both** | 75,718 ( 45,928 to 114,652) | 35,521 (20,892 to 47,432) | 35,521 (20,892 to 47,432) | 30,608 (17,810 to 41,094) | 59.27 (35.95 to 89.75) | 26.88 (15.81 to 35.89) | 24.69 (14.37 to 33.55) | 24.69 (14.37 to 33.55) | -59 (-84 to -10) | -55 (-82 to 0) | -10 (-61 to 105) | -8 (-60 to 112) | -2 (-58 to 126) | 6,794,660 (4,121,026 to 10,289,656) | 3,186,992 (1,874,798 to 4,254,987) | 2,859,783 (1,665,146 to 3,886,254) | 2,746,174 (1,598,180 to 3,686,290) | 5318.82 (3225.91 to 8054.6 8) | 2411.72 (1418.73 to 3219.91) | 2215.01 (1289.72 to 3010.05) | 2167.56 (1261.44 to 2909.59) | -59 (-84 to -10) | -55 (-82 to 0) | -10 (-61 to 105) | -8 (-60 to 112) | -2 (-58 to 126) |  |
| **Female** | 37,883 (17,959 to 63,099) | 16,516 (7,831 to 23,143) | 14,650 (6,923 to 20,507) | 14,041 (6,529 to 19,533) | 61.34 (29.08 to 102.18) | 25.88 (12.27 to 36.26) | 23.49 (11.10 to 32.87) | 22.93 (10.66 to 31.90) | -63 (-90 to 10) | -58 (-88 to 25) | -11 (-71 to 1.60) | -9 (-69 to 168) | -2 (-68 to 187) | 3,399,487 (1,611,241 to 5,663,275) | 1,481,814 (702,611 to 2,076,841) | 1,314,412 (621,171 to 1,840,120) | 1,259,806 (585,867 to 1,752,413) | 5,504.74 (2,609.06 to 9,170.46) | 2,321.93 (1,100.96 to 3,254.31) | 2,107.10 (995.78 to 2,949.85) | 2,057.31 (956.74 to 2,861.75) | -63 (-90 to 10) | -58 (-88 to 25) | -11 (-71 to 1.60) | -9 (-69 to 168) | -2 (-68 to 187) |  |
| **Male** | 37,836 (19,934 to 59,775) | 19,005 (9,435 to 29,682) | 17,224 (8,325 to 26,638) | 16,566 (8,041 to 24,981) | 57.33 (30.21 to 90.58) | 27.81 (13.81 to 43.44) | 25.81 (12.48 to 39.92) | 25.31 (12.28 to 38.16) | -56 (-86 to 26) | -51 (-85 to 44) | -9 (-72 to 176) | -7 (-71 to 189) | -2 (-69 to 206) | 3,395,173 (1,788,727 to 5,364,024) | 1,705,178 (846,505 to 2,663,672) | 1,545,371 (746,950 to 2,389,839) | 1,486,369 (721,571 to 2,241,382) | 5,144.82 (2,710.52 to 8,128.29) | 2,495.57 (1,238.88 to 3,898.36) | 2,315.89 (1,119.38 to 3,581.41) | 2,270.69 (1,102.33 to 3,424.11) | -56 (-86 to 26) | -51 (-85 to 44) | -9 (-72 to 176) | -7 (-71 to 189) | -2 (-69 to 206) |  |

*DALYs* Disability-Adjusted Life Years, *SIDS* sudden infant death syndrome, *UI* uncertainty interval.

**Supplementary Table 4. Age-specific trends in SIDS deaths and DALYs from 1990 to 2021, with percentage change in rates**

| **Age groups** | **Gender** | **Deaths (95% UI)** | | | | | | | | | | | | | **DALYs (95% UI)** | | | | | | | | | | | | | |
| --- | --- | --- | --- | --- | --- | --- | --- | --- | --- | --- | --- | --- | --- | --- | --- | --- | --- | --- | --- | --- | --- | --- | --- | --- | --- | --- | --- | --- |
|  |  | **Number** | | | | **Rate (per 100,000)** | | | | **Percentage change in rates (%)** | | | | | **Number** | | | | **Rate (per 100,000)** | | | | **Percentage change in rates (%)** | | | | |  |
|  |  | **1990** | **2019** | **2020** | **2021** | **1990** | **2019** | **2020** | **2021** | **From 1990 to 2021** | **From 1990 to 2019** | **From 2019 to 2021** | **From 2019 to 2020** | **From 2020 to 2021** | **1990** | **2019** | **2020** | **2021** | **1990** | **2019** | **2020** | **2021** | **From 1990 to 2021** | **From 1990 to 2019** | **From 2019 to 2021** | **From 2019 to 2020** | **From 2020 to 2021** |  |
| **<28 days** | **Both** | 8,875 (4,401 to 14,607) | 3,604 (2,022 to 5,122) | 3,345 (1,861 to 4,804) | 3,232 (1,777 to 4,673) | 88.40 (43.84 to 145.50) | 35.57 (19.96 to 50.55) | 33.83 (18.82 to 48.58) | 33.16 (18.23 to 47.95) | -62 (-87 to 9) | -60 (-86 to 15) | -7 (-64 to 140) | -5 (-63 to 143) | -2 (-62 to 155) | 798,505.15 (395,958.80 to 1,314,279.60) | 324,268.72 (181,936.05 to 460,837.11) | 300,984.94 (167,427.83 to 432,247.12) | 290,793.41 (159,868.56 to 420,411.82) | 7,953.58 (3,943.98 to 13,090.99) | 3,200.32 (1,795.59 to 4,548.16) | 3,043.39 (1,692.94 to 4,370.65) | 2,983.96 (1,640.48 to 4,314.04) | -62 (-87 to 9) | -60 (-86 to 15) | -7 (-64 to 140) | -5 (-63 to 143) | -2 (-62 to 155) |  |
|  | **Female** | 4,845 (1,766 to 8,718) (0 to 0) | 1,770 (811 to 2,637) (0 to 0) | 1,595 (731 to 2,370) (0 to 0) | 1,537 (704 to 2,313) (0 to 0) | 100.00 (36.45 to 179.95) | 36.19 (16.58 to 53.91) | 33.38 (15.30 to 49.61) | 32.65 (14.95 to 49.12) | -67 (-92 to 35) | -64 (-91 to 48) | -10 (-72 to 196) | -8 (-72 to 199) | -2 (-70 to 221) | 435,921 (158,900 to 784,410) | 159,277 (72,964 to 237,300) | 143,494 (65,758 to 213,253) | 138,320 (63,331 to 208,086) | 8,997.49 (3,279.72 to 16,190.39) | 3,255.75 (1,491.43 to 4,850.58) | 3,003.64 (1,376.45 to 4,463.84) | 2,937.46 (1,344.95 to 4,419.08) | -67 (-92 to 35) | -64 (-91 to 48) | -10 (-72 to 196) | -8 (-72 to 199) | -2 (-70 to 221) |  |
|  | **Male** | 4,030 (1,886 to 6,885) (0 to 0) | 1,834 (880 to 2,734) (0 to 0) | 1,750 (822 to 2,616) (0 to 0) | 1,695 (788 to 2,528) (0 to 0) | 77.58 (36.31 to 132.54) | 34.99 (16.80 to 52.16) | 34.24 (16.09 to 51.16) | 33.65 (15.65 to 50.20) | -57 (-88 to 38) | -55 (-87 to 44) | -4 (-70 to 199) | -2 (-69 to 205) | -2 (-69 to 212) | 362,585 (169,728 to 619,446) | 164,991 (79,202 to 245,944) | 157,491 (73,999 to 235,334) | 152,474 (70,908 to 227,491) | 6,979.95 (3,267.36 to 11,924.67) | 3,148.57 (1,511.43 to 4,693.42) | 3,080.55 (1,447.43 to 4,603.17) | 3,027.44 (1,407.91 to 4,516.93) | -57 (-88 to 38) | -55 (-87 to 44) | -4 (-70 to 199) | -2 (-69 to 205) | -2 (-69 to 212) |  |
| **1-5 months** | **Both** | 58,230 (35,908 to 89,079) | 27,069 (16,173 to 36,478) | 24,045 (14,057 to 32,442) | 23,119 (13,568 to 30,900) | 106.66 (65.77 to 163.16) | 48.35 (28.89 to 65.16) | 43.98 (25.71 to 59.34) | 43.01 (25.24 to 57.48) | -60 (-85 to -13) | -55 (-82 to -1) | -11 (-61 to 99) | -9 (-61 to 105) | -2 (-57 to 124) | 5,226,734 (3,223,140 to 7,995,790) | 2,429,724.67 (1,451,738.70 to 3,274,313.95) | 2,158,284.10 (1,261,730.99 to 2,911,986.44) | 2,075,162.21 (1,217,849.49 to 2,773,591.85) | 9,573.53 (5,903.65 to 14,645.46) | 4,340.14 (2,593.20 to 5,848.81) | 3,947.50 (2,307.70 to 5,326.02) | 3,860.26 (2,265.47 to 5,159.50) | -60 (-85 to -13) | -55 (-82 to -1) | -11 (-61 to 99) | -9 (-61 to 105) | -2 (-57 to 124) |  |
|  | **Female** | 28,721 (13,996 to 48,541) (0 to 0) | 12,408 (5,985 to 17,645) (0 to 0) | 10,927 (5,177 to 15,162) (0 to 0) | 10,484 (4,903 to 14,483) (0 to 0) | 108.85 (53.04 to 183.96) | 45.89 (22.14 to 65.26) | 41.36 (19.60 to 57.39) | 40.35 (18.87 to 55.74) | -63 (-90 to 5) | -58 (-88 to 23) | -12 (-71 to 152) | -10 (-70 to 159) | -2 (-67 to 184) | 2,577,970 (1,256,296 to 4,357,103) | 1,113,733 (537,260 to 1,583,845) | 980,825 (464,704 to 1,360,917) | 941,059 (440,052 to 1,300,011) | 9,770.12 (4,761.17 to 16,512.77) | 4,119.04 (1,987.01 to 5,857.70) | 3,712.53 (1,758.95 to 5,151.22) | 3,621.67 (1,693.54 to 5,003.09) | -63 (-90 to 5) | -58 (-88 to 23) | -12 (-71 to 152) | -10 (-70 to 159) | -2 (-67 to 184) |  |
|  | **Male** | 29,509 (15,718 to 46,693) (0 to 0) | 14,661 (7,349 to 22,214) (0 to 0) | 13,118 (6,489 to 19,670) (0 to 0) | 12,635 (6,327 to 19,135) (0 to 0) | 104.61 (55.72 to 165.52) | 50.65 (25.39 to 76.75) | 46.43 (22.97 to 69.61) | 45.49 (22.78 to 68.90) | -57 (-86 to 24) | -52 (-85 to 38) | -10 (-70 to 171) | -8 (-70 to 174) | -2 (-67 to 200) | 2,648,765 (1,410,879 to 4,191,194) | 1,315,991 (659,674 to 1,993,952) | 1,177,459 (582,468 to 1,765,588) | 1,134,103 (567,880 to 1,717,593) | 9,389.64 (5,001.44 to 14,857.42) | 4,546.70 (2,279.15 to 6,889.02) | 4,167.20 (2,061.44 to 6,248.68) | 4,083.49 (2,044.73 to 6,184.42) | -57 (-86 to 24) | -52 (-85 to 38) | -10 (-70 to 171) | -8 (-70 to 174) | -2 (-67 to 200) |  |
| **6-11 months** | **Both** | 8,614 (4,820 to 12,673) | 4,848 (2,186 to 8,275) | 4,484 (1,973 to 7,772) | 4,257 (1,884 to 7,390) | 13.65 (7.64 to 20.08) | 7.34 (3.31 to 12.53) | 6.95 (3.06 to 12.04) | 6.74 (2.98 to 11.69) | -51 (-85 to 53) | -46 (-84 to 64) | -8 (-76 to 253) | -5 (-76 to 264) | -3 (-75 to 283) | 769,420 (430,531 to 1,131,958) | 432,999.06 (195,286.14 to 739,074.48) | 400,514.32 (176,236.15 to 694,179.88) | 380,218.88 (168,310.59 to 660,100.54) | 1,219.13 (682.17 to 1,793.56) | 655.75 (295.75 to 1,119.28) | 620.52 (273.05 to 1,075.50) | 601.69 (266.35 to 1,044.59) | -51 (-85 to 53) | -46 (-84 to 64) | -8 (-76 to 253) | -5 (-76 to 264) | -3 (-75 to 283) |  |
|  | **Female** | 4,317 (2,198 to 6,916) (0 to 0) | 2,338 (1,011 to 3,702) (0 to 0) | 2,128 (882 to 3,402) (0 to 0) | 2,020 (824 to 3,202) (0 to 0) | 14.14 (7.20 to 22.66) | 7.33 (3.17 to 11.61) | 6.82 (2.83 to 10.91) | 6.61 (2.70 to 10.48) | -53 (-88 to 46) | -48 (-86 to 61) | -10 (-77 to 231) | -7 (-76 to 244) | -3 (-75 to 271) | 385,596 (196,348 to 617,707) | 208,803 (90,304 to 330,642) | 190,092 (78,736 to 303,859) | 180,427 (73,565 to 286,009) | 1,263.24 (643.25 to 2,023.65) | 654.82 (283.20 to 1,036.91) | 609.59 (252.49 to 974.42) | 590.74 (240.86 to 936.43) | -53 (-88 to 46) | -48 (-86 to 61) | -10 (-77 to 231) | -7 (-76 to 244) | -3 (-75 to 271) |  |
|  | **Male** | 4,297 (1,968 to 7,010) (0 to 0) | 2,510 (970 to 4,799) (0 to 0) | 2,356 (905 to 4,617) (0 to 0) | 2,237 (863 to 4,363) (0 to 0) | 13.19 (6.04 to 21.51) | 7.35 (2.84 to 14.06) | 7.06 (2.71 to 13.84) | 6.85 (2.64 to 13.36) | -48 (-88 to 121) | -44 (-87 to 133) | -7 (-81 to 371) | -4 (-81 to 387) | -3 (-81 to 393) | 383,824 (175,776 to 626,142) | 224,196 (86,617 to 428,682) | 210,422 (80,811 to 412,396) | 199,792 (77,043 to 389,731) | 1,177.81 (539.39 to 1,921.39) | 656.62 (253.68 to 1,255.51) | 630.74 (242.23 to 1,236.16) | 611.93 (235.97 to 1,193.68) | -48 (-88 to 121) | -44 (-87 to 133) | -7 (-81 to 371) | -4 (-81 to 387) | -3 (-81 to 393) |  |

*DALYs* Disability-Adjusted Life Years, *SIDS* sudden infant death syndrome, *UI* uncertainty interval.

**Supplementary Table 5. SDI-specific trends in SIDS deaths and DALYs from 1990 to 2021, with percentage change in rates**

| **SDI**  **Level** | **Deaths (95% UI)** | | | | | | | | | | | | | **DALYs (95% UI)** | | | | | | | | | | | | | | | | |  |
| --- | --- | --- | --- | --- | --- | --- | --- | --- | --- | --- | --- | --- | --- | --- | --- | --- | --- | --- | --- | --- | --- | --- | --- | --- | --- | --- | --- | --- | --- | --- | --- |
|  | **Number** | | | | **Rate (per 100,000)** | | | | **Percentage change in rates (%)** | | | | | **Number** | | | | **Rate (per 100,000)** | | | | **Percentage change in rates (%)** | | | | | | | | |  |
|  | **1990** | **2019** | **2020** | **2021** | **1990** | **2019** | **2020** | **2021** | **From 1990 to 2021** | **From 1990 to 2019** | **From 2019 to 2021** | **From 2019 to 2020** | **From 2020 to 2021** | **1990** | **2019** | **2020** | **2021** | **1990** | **2019** | **2020** | **2021** | **From 1990 to 2021** | **From 1990 to 2019** | | **From 2019 to 2021** | | **From 2019 to 2020** | | **From 2020 to 2021** | |  |
| **Low SDI** | 19,634 (9,630 to 31,308) | 16,230 (8,594 to 23,703) | 15,127 (7,966 to 22,232) | 14,785 (7,697 to 21,646) | 95.98 (47.08 to 153.05) | 47.87 (25.35 to 69.92) | 44.23 (23.29 to 65) | 42.8 (22.28 to 62.67) | -50 (-83 to 49) | -55 (-85 to 33) | -11 (-68 to 147) | -8 (-67 to 156) | -3 (-66 to 169) | 1,761,821 (864,012 to 2,809,316) | 1,456,060 (770,998 to 2,126,825) | 1,357,086 (714,664 to 1,994,516) | 1,326,431 (690,406 to 1,942,045) | 8,613.06 (4,223.91 to 13,733.97) | 4,294.89 (2,274.18 to 6,273.42) | 3,967.66 (2,089.44 to 5,831.29) | 3,840.09 (1,998.76 to 5,622.32) | -50 (-83 to 49) | | -55 (-85 to 33) | | -11 (-68 to 147) | | -8 (-67 to 156) | | -3 (-66 to 169) | |
| **Low-**  **middle SDI** | 29,785 (14,481 to 54,150) | 11,620 (6,366 to 17,252) | 9,830 (5,370 to 14,736) | 9,345 (5,131 to 13,812) | 81.65 (39.69 to 148.43) | 29.81 (16.34 to 44.27) | 25.58 (13.97 to 38.34) | 24.6 (13.51 to 36.36) | -63 (-89 to 12) | -70 (-91 to -8) | -17 (-69 to 123) | -14 (-68 to 1.35) | -04 (-65 to 160) | 2,672,926 (1,299,197 to 4,859,911) | 1,042,544 (571,234 to 1,547,660) | 882,015 (481,890 to 1,321,888) | 838,442 (460,422 to 1,239,118) | 7,326.94 (3,561.32 to 13,321.84) | 2,675.06 (1,465.73 to 3,971.13) | 2,294.98 (1,253.87 to 3,439.52) | 2,207.37 (1,212.16 to 3,262.24) | -63 (-89 to 12) | | -70 (-91 to -8) | | -17 (-69 to 123) | | -14 (-68 to 1.35) | | -04 (-65 to 160) | |
| **Middle SDI** | 11,021 (6,502 to 15,784) | 4,446 (2,788 to 6,010) | 3,872 (2,417 to 5,243) | 3,593 (2,232 to 4,970) | 27.33 (16.12 to 39.14) | 12.74 (7.99 to 17.22) | 11.63 (7.26 to 15.75) | 11.26 (7 to 15.58) | -53 (-80 to 7) | -59 (-82 to -3) | -12 (-59 to 95) | -9 (-58 to 97) | -3 (-56 to 1.15) | 989,023 (583,424 to 1,416,389) | 398,976 (250,237 to 539,364) | 347,466 (216,869 to 470,393) | 322,451 (200,345 to 446,090) | 2,452.23 (1,446.57 to 3,511.86) | 1,143.37 (717.12 to 1,545.68) | 1,043.58 (651.356 to 1,412.78) | 1,010.71 (627.98 to 1,398.25) | -53 (-80 to 7) | | -59 (-82 to -3) | | -12 (-59 to 95) | | -9 (-58 to 97) | | -3 (-56 to 1.15) |  |
| **High-**  **middle SDI** | 4,232 (3,079 to 5,965) | 1,169 (874 to 1,482) | 1,139 (819 to 1,474) | 1,058 (746 to 1,371) | 23.46 (17.07 to 33.07) | 8.58 (6.42-10.88) | 8.98 (6.46 to 11.63) | 8.89 (6.27 to 11.52) | -63 (-81 to -36) | -62 (-81 to -33) | 4 (-42 to 79) | 5 (-41 to 81) | -1 (-46 to 78) | 379,695 (276,271 to 535,135) | 104,931 (78,478 to 133,042) | 102,183 (73,525 to 132,293) | 94,943 (66,953 to 123,029) | 2,104.96 (1,531.60 to 2,966.70) | 770.01 (575.89 to 976.29) | 806.05 (579.99 to 1,043.56) | 797.57 (562.44 to 1,033.51) | -63 (-81 to -36) | | -62 (-81 to -33) | | 4 (-42 to 79) | | 5 (-41 to 81) | | -1 (-46 to 78) |  |
| **High SDI** | 11,006 (10,626 to 11,372) | 2,033 (1,890 to 2,189) | 1,884 (1,695 to 2,081) | 1,805 (1,576 to 2,043) | 89.2 (86.12 to 92.17) | 19.1 (17.75 to 20.56) | 18.12 (16.3 to 20.01) | 17.6 (15.36 to 19.92) | -79 (-81 to -76) | -80 (-83 to -77) | -8 (-25 to 12) | -5 (-21 to 13) | -3 (-23 to 22) | 987,580 (953,398 to 1,020,385) | 182,417 (169,586 to 196,392) | 169,074 (152,130 to 186,742) | 162,001 (141,387 to 183,365) | 8,004.17 (7,727.13 to 8,270.04) | 1,713.59 (1,593.05 to 1,844.86) | 1,626.04 (1,463.08 to 1,795.96) | 1,578.99 (1,378.06 to 1,787.21) | -79 (-81 to -76) | | -80 (-83 to -77) | | -8 (-25 to 12) | | -5 (-21 to 13) | | -3 (-23 to 22) |  |

*DALYs* Disability-Adjusted Life Years, *SIDS* sudden infant death syndrome, *UI* uncertainty interval.

**Supplementary Table 6. Health system grouping levels-specific trends in SIDS deaths and DALYs from 1990 to 2021, with percentage change in rates**

| **Health system grouping**  **levels** | **Deaths (95% UI)** | | | | | | | | | | | | | **DALYs (95% UI)** | | | | | | | | | | | | |
| --- | --- | --- | --- | --- | --- | --- | --- | --- | --- | --- | --- | --- | --- | --- | --- | --- | --- | --- | --- | --- | --- | --- | --- | --- | --- | --- |
|  | **Number** | | | | **Rate (per 100,000)** | | | | **Percentage change in rates (%)** | | | | | **Number** | | | | **Rate (per 100,000)** | | | | **Percentage change in rates (%)** | | | | |
|  | **1990** | **2019** | **2020** | **2021** | **1990** | **2019** | **2020** | **2021** | **From 1990 to 2021** | **From 1990 to 2019** | **From 2019 to 2021** | **From 2019 to 2020** | **From 2020 to 2021** | **1990** | **2019** | **2020** | **2021** | **1990** | **2019** | **2020** | **2021** | **From 1990 to 2021** | **From 1990 to 2019** | **From 2019 to 2021** | **From 2019 to 2020** | **From 2020 to 2021** |
| **Minimal Health System** | 5,106 (2,354 to 7,814) | 5,134 (2,496 to 8,033) | 5,084 (2,522 to 7,748) | 5,030 (2,499 to 7,574) | 85.77 (39.54 to 131.27) | 43.52 (21.16 to 68.09) | 42.36 (21.02 to 64.56) | 41.19 (20.47 to 62.03) | -52 (-84 to 57) | -49 (-84 to 72) | -5 (-70 to 193) | -3 (-69 to 205) | -3 (-68 to 195) | 458,122 (211,258 to 701,163) | 460,606 (223,984 to 720,404) | 456,117 (226,348 to 695,017) | 451,235 (224,302 to 679,254) | 7,695.56 (3,548.73 to 11,778.18) | 3,904.50 (1,898.69 to 6,106.77) | 3,800.42 (1,885.95 to 5,790.95) | 3,695.79 (1,837.12 to 5,563.35) | -52 (-84 to 57) | -49 (-84 to 72) | -5 (-70 to 193) | -3 (-69 to 205) | -3 (-68 to 195) |
| **Limited Health System** | 42,392 (20,449 to 74,509) | 22,802 (12,628 to 31,177) | 19,809 (10,756 to 27,433) | 18,995 (10,222 to 26,334) | 82.89 (39.98 to 145.69) | 37.2 (20.6 to 50.86) | 32.56 (17.68 to 45.09) | 31.42 (16.91 to 43.56) | -62 (-88 to 9) | -55 (-86 to 27) | -16 (-67 to 111) | -12 (-65 to 1.19) | -4 (-63 to 146) | 3,804,228 (1,834,604 to 6,687,257) | 2,045,751 (1,132,996 to 2,797,439) | 1,777,143 (964,940 to 2,461,217) | 1,704,178 (917,101 to 2,362,201) | 7,438.46 (3,587.23 to 13,075.69) | 3,337.21 (1,848.24 to 4,563.43) | 2,921.23 (1,586.15 to 4,045.70) | 2,818.72 (1,516.89 to 3,907.09) | -62 (-88 to 9) | -55 (-86 to 27) | -16 (-67 to 111) | -12 (-65 to 1.19) | -4 (-63 to 146) |
| **Basic Health System** | 14,759 (9,328 to 21,852) | 4,923 (3,168 to 6,672) | 4,512 (2,837 to 6,164) | 4,224 (2,633 to 5,869) | 28.47 (18 to 42.16) | 11.26 (7.24 to 15.26) | 10.91 (6.86 to 14.91) | 10.73 (6.69 to 14.91) | -62 (-84 to -17) | -60 (-83 to -15) | -5 (-56 to 106) | -3 (-55 to 106) | -2 (-55 to 117) | 1,324,390 (837,140 - 1,961,206) | 441,870 (284,325 to 598,875) | 404,892 (254,572 to 553,168) | 379,055 (236,286 to 526,720) | 2,554.96 (1,614.98 to 3,783.48) | 1,010.35 (650.12 to 1,369.35) | 979.32 (615.74 to 1,337.95) | 963.19 (600.41 to 1,338.41) | -62 (-84 to -17) | -60 (-83 to -15) | -5 (-56 to 106) | -3 (-55 to 106) | -2 (-55 to 117) |
| **Advanced Health System** | 13,422 (12,426 to 14,656) | 2,638 (2,419 to 2,863) | 2,448 (2,187 to 2,710) | 2,338 (2,042 to 2,651) | 71.73 (66.41 to 78.33) | 17.34 (15.9 to 18.82) | 16.51 (14.75 to 18.28) | 16.05 (14.02 to 18.19) | -78 (-82 to -73) | -76 (-80 to -72) | -7 (-26 to 14) | -5 (-22 to 15) | -3 (-23 to 23) | 1,204,305 (1,115,022 to 1,314,968) | 236,703 (217,033 to 256,877) | 219,672 (196,233 to 243,217) | 209,800 (183,257 to 237,878) | 6,435.93 (5,958.79 to 7,027.32) | 1,556.23 (1,426.91 to 1,688.87) | 1,481.79 (1,323.68 to 1,640.61) | 1,439.99 (1,257.80 to 1,632.70) | -78 (-82 to -73) | -76 (-80 to -72) | -7 (-26 to 14) | -5 (-22 to 15) | -3 (-23 to 23) |

*DALYs* Disability-Adjusted Life Years, *SIDS* sudden infant death syndrome, *UI* uncertainty interval.

**Supplementary Table 7. Future forecasts of global rates of deaths and DALYs of SIDS from 2021 to 2035 by BAPC model**

|  | **Death rate (95% UI)** | | | **DALYs rate (95% UI)** | | |
| --- | --- | --- | --- | --- | --- | --- |
|  | **Both** | **Male** | **Female** | **Both** | **Male** | **Female** |
| **2021** | 24.69 | 25.31 | 22.93 | 2167.56 | 2270.69 | 2057.31 |
| **The predicted value of BAPC model** | | | | | | |
| **2022** | 23.40 (22.05 to 24.75) | 24.67 (23.23 to 26.11) | 22.09 (20.68 to 23.49) | 2,109.60 (2,011.61 to 2,207.60) | 2,240.67 (2,145.91 to 2,335.43) | 1,972.47 (1,869.08 to 2,075.85) |
| **2023** | 22.61 (20.76 to 24.46) | 23.96 (22.04 to 25.89) | 21.22 (19.28 to 23.16) | 2,042.80 (1,913.92 to 2,171.67) | 2,192.19 (2,074.71 to 2,309.68) | 1,889.18 (1,745.12 to 2,033.24) |
| **2024** | 21.88 (19.42 to 24.34) | 23.31 (20.78 to 25.84) | 20.41 (17.84 to 22.99) | 1,978.81 (1,810.45 to 2,147.18) | 2,147.24 (1,999.41 to 2,295.07) | 1,808.64 (1,614.55 to 2,002.74) |
| **2025** | 21.21 (18.07 to 24.34) | 22.71 (19.51 to 25.90) | 19.67 (16.39 to 22.95) | 1,917.58 (1,703.38 to 2,131.79) | 2,104.15 (1,919.20 to 2,289.10) | 1,732.20 (1,482.31 to 1,982.10) |
| **2026** | 20.58 (16.73 to 24.44) | 22.15 (18.23 to 26.07) | 18.98 (14.96 to 22.99) | 1,858.25 (1,593.02 to 2,123.48) | 2,061.92 (1,833.49 to 2,290.35) | 1,659.00 (1,349.29 to 1,968.71) |
| **2027** | 20.01 (15.40 to 24.62) | 21.64 (16.96 to 26.31) | 18.34 (13.56 to 23.11) | 1,800.75 (1,481.06 to 2,120.44) | 2,020.54 (1,743.80 to 2,297.29) | 1,588.89 (1,217.39 to 1,960.39) |
| **2028** | 19.48 (14.10 to 24.87) | 21.16 (15.71 to 26.62) | 17.74 (12.19 to 23.29) | 1,745.04 (1,368.71 to 2,121.36) | 1,980.00 (1,651.30 to 2,308.69) | 1,521.74 (1,087.84 to 1,955.64) |
| **2029** | 18.99 (12.82 to 25.17) | 20.73 (14.46 to 27.00) | 17.19 (10.86 to 23.53) | 1,691.04 (1,256.81 to 2,125.27) | 1,940.26 (1,556.85 to 2,323.67) | 1,457.43 (961.50 to 1,953.36) |
| **2030** | 18.55 (11.56 to 25.54) | 20.33 (13.23 to 27.43) | 16.69 (9.56 to 23.81) | 1,638.72 (1,146.01 to 2,131.43) | 1,901.33 (1,461.12 to 2,341.53) | 1,395.84 (838.97 to 1,952.71) |
| **2031** | 18.14 (10.33 to 25.95) | 19.97 (12.02 to 27.92) | 16.22 (8.29 to 24.14) | 1,588.01 (1,036.78 to 2,139.25) | 1,863.17 (1,364.61 to 2,361.73) | 1,336.85 (720.64 to 1,953.06) |
| **2032** | 17.77 (9.12 to 26.42) | 19.64 (10.82 to 28.46) | 15.79 (7.06 to 24.51) | 1,538.88 (929.49 to 2,148.27) | 1,825.78 (1,267.74 to 2,383.82) | 1,280.35 (606.82 to 1,953.89) |
| **2033** | 17.43 (7.93 to 26.94) | 19.34 (9.63 to 29.06) | 15.39 (5.86 to 24.92) | 1,491.26 (824.44 to 2,158.09) | 1,789.14 (1,170.84 to 2,407.44) | 1,226.25 (497.68 to 1,954.81) |
| **2034** | 17.13 (6.75 to 27.51) | 19.08 (8.45 to 29.70) | 15.02 (4.68 to 25.37) | 1,445.12 (721.84 to 2,168.40) | 1,753.23 (1,074.19 to 2,432.28) | 1,174.43 (393.36 to 1,955.49) |
| **2035** | 16.86 (5.59 to 28.12) | 18.84 (7.28 to 30.40) | 14.69 (3.53 to 25.85) | 1,400.41 (621.88 to 2,178.93) | 1,718.05 (978.02 to 2,458.08) | 1,124.80 (293.90 to 1,955.69) |

*DALYs* Disability-Adjusted Life Years, *SIDS* sudden infant death syndrome, *UI* uncertainty interval.

**Supplementary Table 8. Future forecasts of SIDS mortality and DALYs rates for males in the 1-5 month age group in Low SDI regions, Western Sub-Saharan Africa, and Minimal health system grouping levle from 2021 to 2030 by ARIMA model**

|  | **Death rate (95% UI)** | | | | **DALYs rate (95% UI)** | | | |
| --- | --- | --- | --- | --- | --- | --- | --- | --- |
|  | **Global** | **Low SDI** | **Western Sub-Saharan Africa** | **Minimal Health System** | **Global** | **Low SDI** | **Western Sub-Saharan Africa** | **Minimal Health System** |
| **2021** | 45.493 | 74.32 | 82.55 | 76.99 | 4083.48 | 6671.30 | 7409.89 | 6911.12 |
| **The predicted value of ARIMA model** | | | | | | | | |
| **2022** | 43.59 (41.92 to 45.25) | 69.16 (66.72 to 71.60) | 80.45 (77.80 to 83.09) | 74.91 (73.42 to 76.40) | 3,912.32 (3,763.12 to 4,061.52) | 6,207.84 (5,989.03 to 6,426.65) | 7,221.13 (6,983.66 to 7,458.60) | 6,723.94 (6,590.44 to 6,857.43) |
| **2023** | 41.68 (39.33 to 44.03) | 65.70 (61.47 to 69.93) | 78.71 (73.87 to 83.54) | 72.82 (68.66 to 76.99) | 3,741.16 (3,530.16 to 3,952.15) | 5,897.23 (5,517.71 to 6,276.76) | 7,064.67 (6,630.54 to 7,498.81) | 6,536.75 (6,162.74 to 6,910.76) |
| **2024** | 39.77 (36.89 to 42.65) | 61.25 (54.47 to 68.03) | 77.15 (70.34 to 83.96) | 70.74 (63.19 to 78.29) | 3,569.99 (3,311.57 to 3,828.41) | 5,497.55 (4,889.00 to 6,106.10) | 6,925.35 (6,314.13 to 7,536.57) | 6,349.57 (5,671.78 to 7,027.35) |
| **2025** | 37.87 (34.54 to 41.19) | 57.37 (47.87 to 66.88) | 75.70 (67.14 to 84.26) | 68.65 (57.13 to 80.18) | 3,398.82 (3,100.43 to 3,697.22) | 5,149.78 (4,296.58 to 6,002.97) | 6,795.12 (6,026.70 to 7,563.54) | 6,162.38 (5,128.18 to 7,196.58) |
| **2026** | 35.96 (32.24 to 39.68) | 53.16 (40.54 to 65.79) | 74.31 (64.19 to 84.43) | 66.57 (50.56 to 82.57) | 3,227.66 (2,894.04 to 3,561.28) | 4,771.75 (3,638.71 to 5,904.79) | 6,669.71 (5,761.30 to 7,578.11) | 5,975.20 (4,538.66 to 7,411.73) |
| **2027** | 34.05 (29.98 to 38.12) | 49.15 (33.17 to 65.12) | 72.94 (61.41 to 84.46) | 64.48 (43.54 to 85.43) | 3,056.49 (2,691.03 to 3,421.95) | 4,411.35 (2,977.72 to 5,844.99) | 6,546.85 (5,512.59 to 7,581.12) | 5,788.01 (3,907.91 to 7,668.11) |
| **2028** | 32.14 (27.75 to 36.54) | 45.02 (25.42 to 64.62) | 71.58 (58.79 to 84.38) | 62.40 (36.09 to 88.71) | 2,885.33 (2,490.58 to 3,280.07) | 4,040.68 (2,281.36 to 5,800.01) | 6,425.36 (5,276.68 to 7,574.03) | 5,600.83 (3,239.42 to 7,962.23) |
| **2029** | 30.24 (25.54 to 34.94) | 40.96 (17.50 to 64.41) | 70.24 (56.27 to 84.21) | 60.31 (28.25 to 92.37) | 2,714.16 (2,292.16 to 3,136.16) | 3,676.00 (1,570.78 to 5,781.22) | 6,304.58 (5,050.77 to 7,558.38) | 5,413.64 (2,535.94 to 8,291.34) |
| **2030** | 28.33 (23.34 to 33.32) | 36.85 (9.31 to 64.39) | 68.90 (53.84 to 83.95) | 58.23 (20.05 to 96.40) | 2,542.99 (2,095.40 to 2,990.59) | 3,307.83 (835.94 to 5,779.72) | 6,184.18 (4,832.85 to 7,535.51) | 5,226.46 (1,799.67 to 8,653.24) |

*DALYs* Disability-Adjusted Life Years, *SIDS* sudden infant death syndrome, *UI* uncertainty interval.

**Supplementary Figure 1. Global number and rates of (A) deaths and (B) DALYs of SIDS from 1990 to 2021**

**
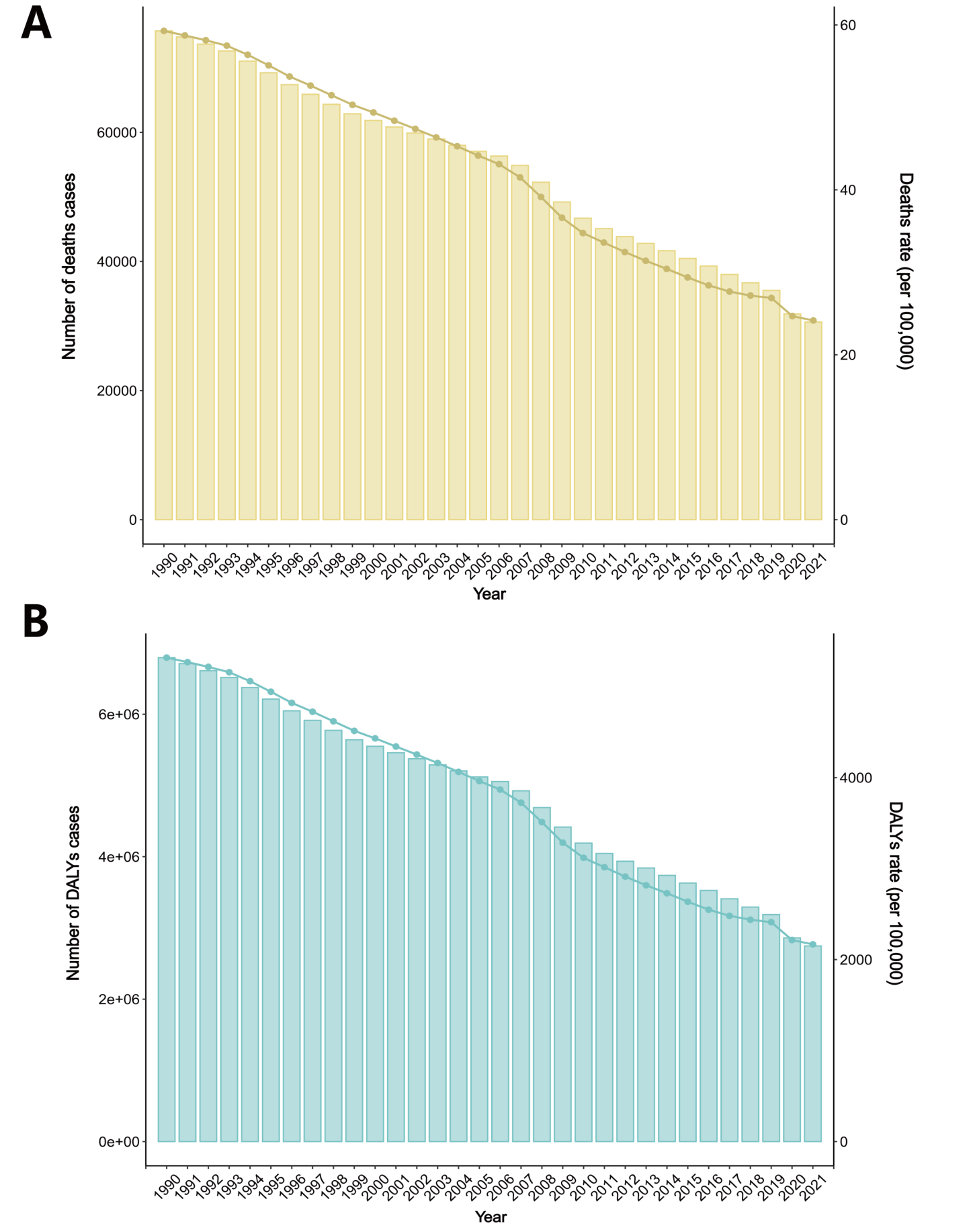
**

**Supplementary Figure 2. Global number and rates of (A) deaths and (B) DALYs of SIDS by sex from 1990 to 2021**

**
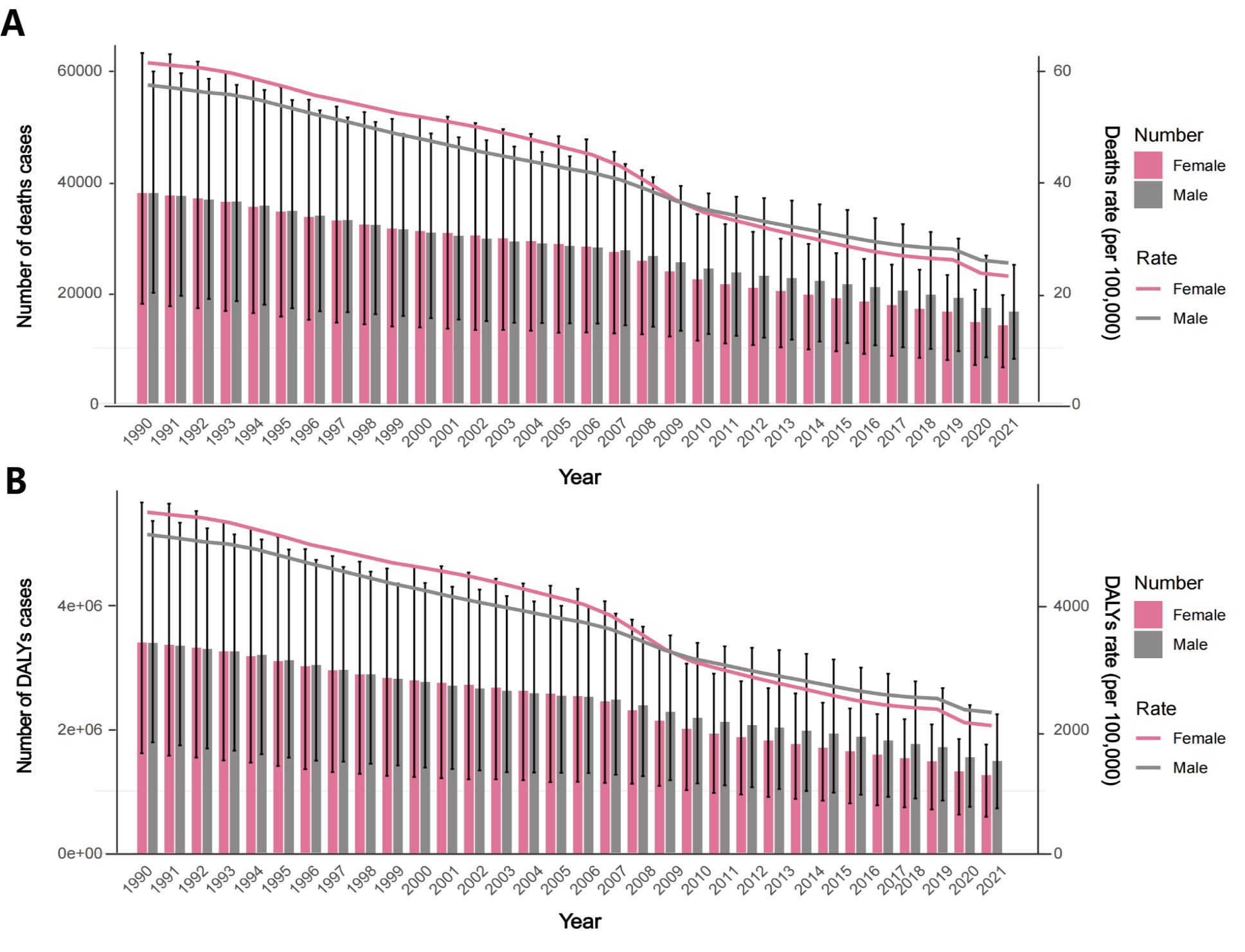
**

**Supplementary Figure 3. Global number and rates of (A) deaths and (B) DALYs of SIDS by age from 1990 to 2021**

**
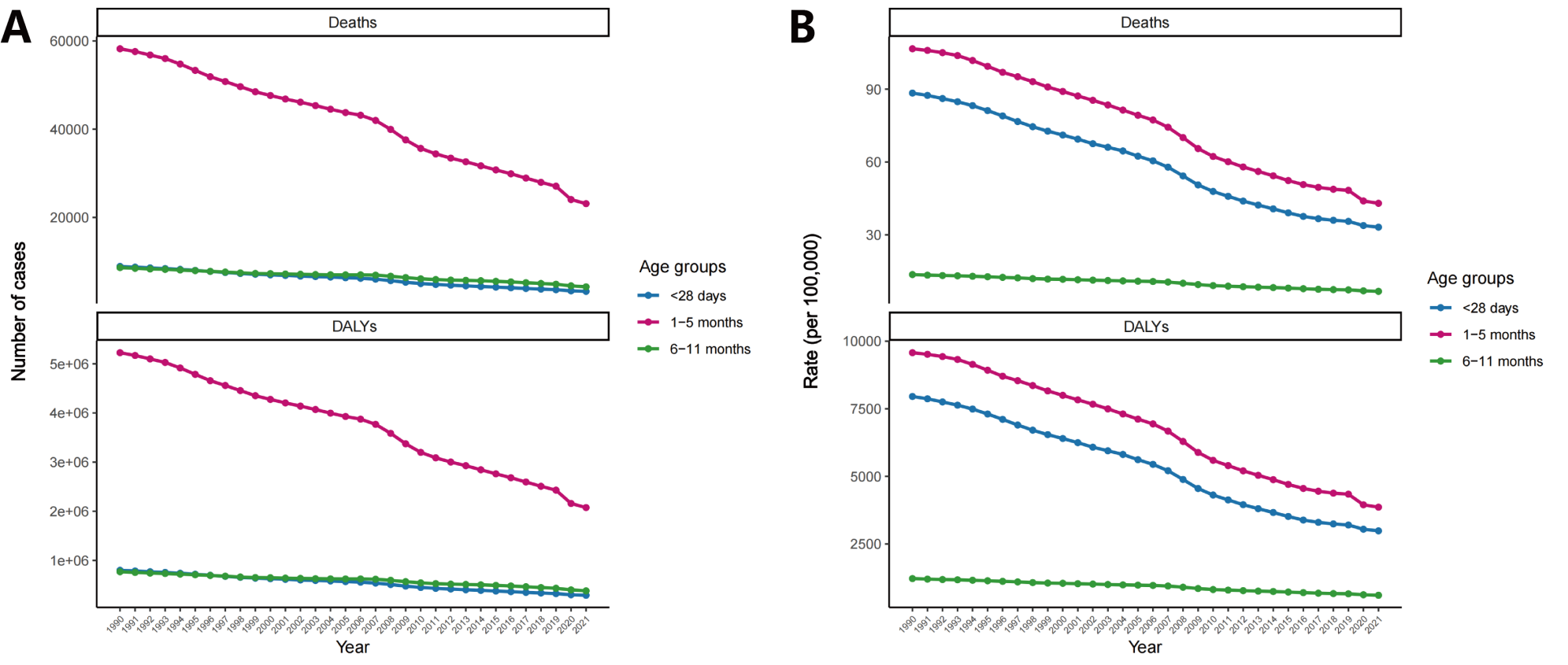
**

**Supplementary Figure 4. Global number and rates of (A) deaths and (B) DALYs of SIDS by SDI from 1990 to 2021**

**
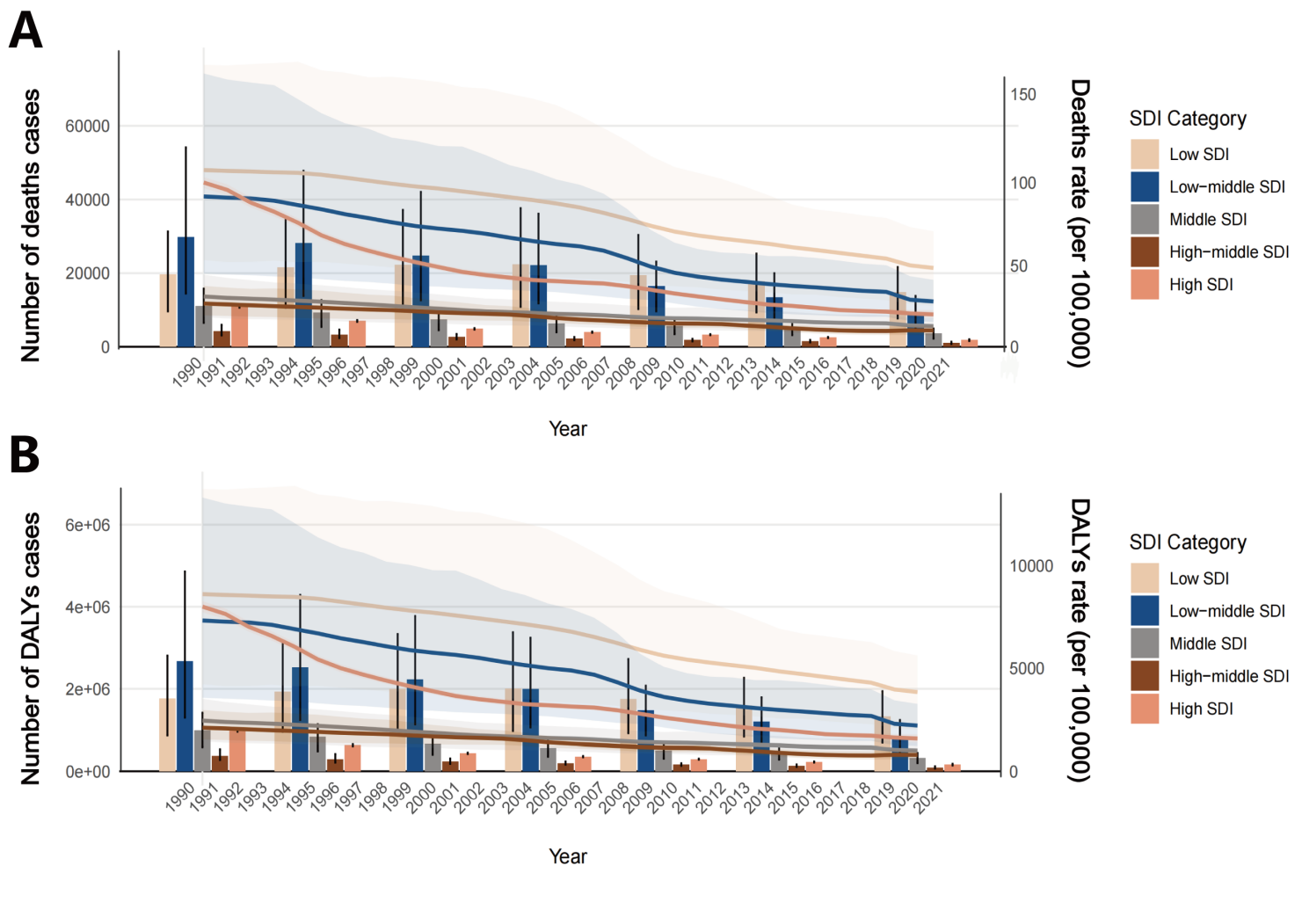
**

**Supplementary Figure 5. The association between SIDS (A) mortality and (B) DALYs rates and SDI in GBD regions**

**
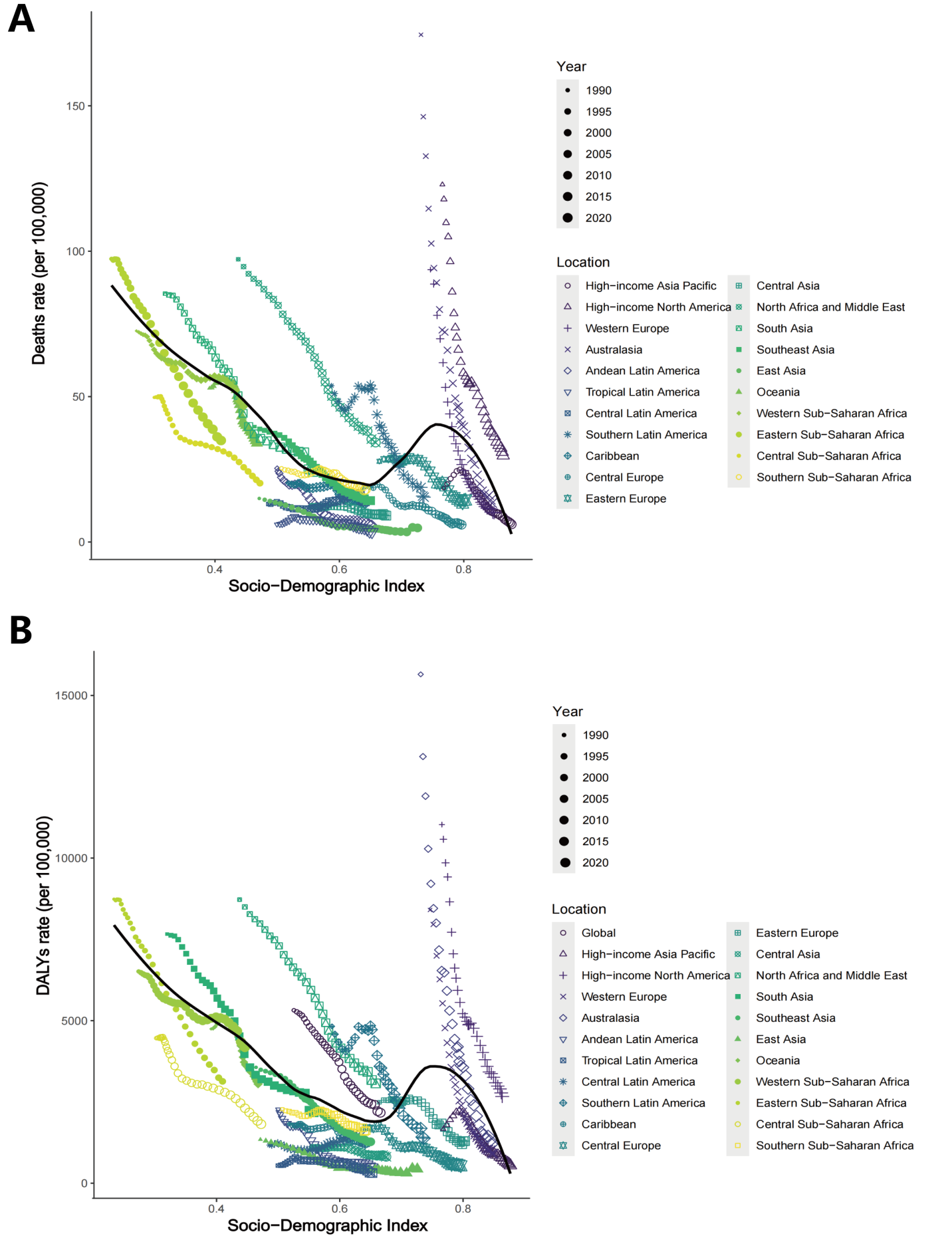
**

**Supplementary Figure 6. Global number and rates of (A) deaths and (B) DALYs of SIDS by health system grouping levels from 1990 to 2021**

**
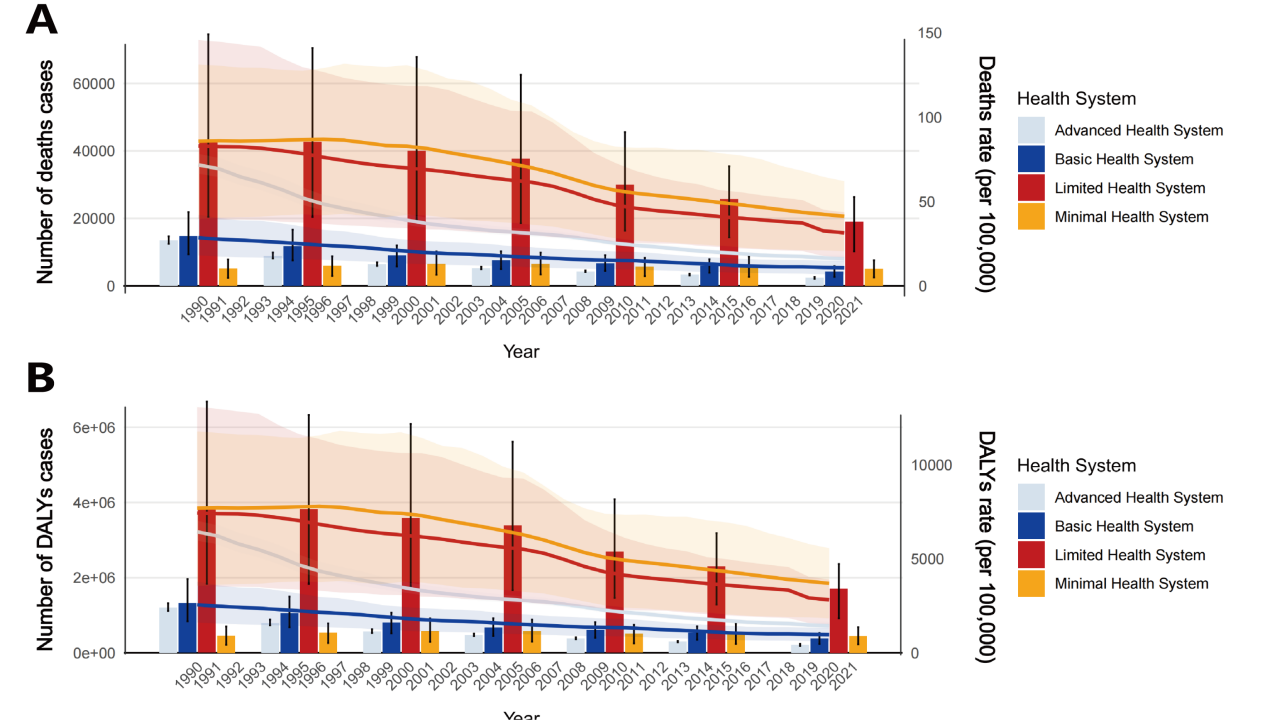
**

**Supplementary Figure 7. Future forecasts of SIDS mortality and DALYs rates for males in the 1-5 month age group in (A) Low SDI regions, (B) Western Sub-Saharan Africa, and (C) Minimal health system grouping levles from 2021 to 2030 by ARIMA model**

**
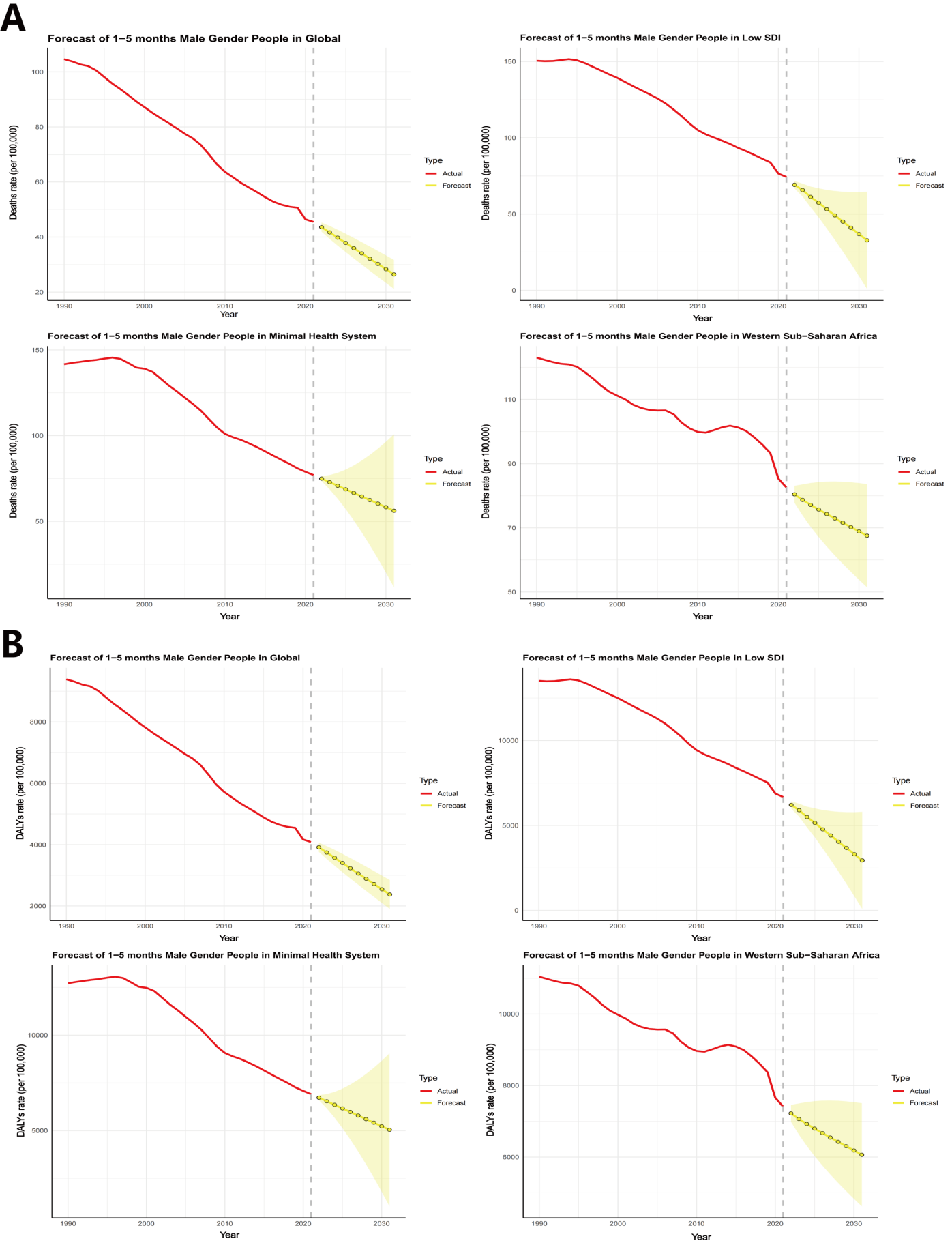
**
